# Supplementary material for: Syntheses and reactivities of strained fused-ring metallaaromatics containing planar eleven-carbon chains
Source: Nat Commun. 2024 May 23;15:4378. doi: 10.1038/s41467-024-48835-8 (PMC11116401; doi:10.1038/s41467-024-48835-8)
Supplement: Supplementary file 1 — Supplementary Information [file 41467_2024_48835_MOESM1_ESM.pdf]

Supplementary Information

Syntheses and Reactivities of Strained Fused-Ring Metallaaromatics Containing Planar Eleven-Carbon Chains

Binbin Xu[1,3], Wei Mao[1,3], Zhengyu Lu[1,3], Yuanting Cai[1], Dafa Chen[1,*] & Haiping Xia[1,2,*]

[1]Shenzhen Grubbs Institute and Guangdong Provincial Key Laboratory of Catalysis, Department of Chemistry, Southern University of Science and Technology, Shenzhen 518055, China

[2]Southern University of Science and Technology Guangming Advanced Research Institute, Shenzhen 518055, China

[3]These authors contributed equally to this work

*email: xiahp@sustech.edu.cn; chendf@sustech.edu.cn

Table of Contents

1. General information **S2**

2. Synthesis and characterization **S3**

3. Proposed mechanism **S21**

4. Resonance structures of 4 **S22**

5. NMR spectra **S23**

6. HRMS spectra **S51**

7. Crystallographic details **S62**

8. Theoretical calculations **S76**

9. Photothermal performance **S84**

10. References **S85**

1. General information

All syntheses were carried out under an inert atmosphere (nitrogen or argon) using standard Schlenk techniques unless otherwise stated. Compound **1** was synthesized according to the published literatures[1]. The other reagents and solvents were used as purchased from commercial sources without further purification. Column chromatography was performed on silica gel (200–300 mesh) in air. NMR spectra was collected on the Brucker AVIII-400 (400 MHz) or Brucker AVIII-600 spectrometer (600 MHz). ^{1}H and $^{13}C\{^{1}H\}$ NMR chemical shifts (δ) are relative to tetramethylsilane, and $^{31}P\{^{1}H\}$ NMR chemical shifts are relative to 85% H_3PO_4. Two-dimensional and one-dimensional NMR spectra are abbreviated as HSQC (heteronuclear single quantum coherence), HMBC (heteronuclear multiple bond coherence). The absolute values of the coupling constants are given in hertz (Hz). Multiplicities are abbreviated as s (singlet), d (doublet), t (triplet), q (quartet), m (multiplet) and br (broad). High resolution mass spectra (HRMS) experiments were recorded on a Thermo Scientific Q Exactive instrument. UV-Vis-NIR absorption spectra were recorded on a UV3600 spectrometer (Shimadzu, Japan).

2. Synthesis and characterization

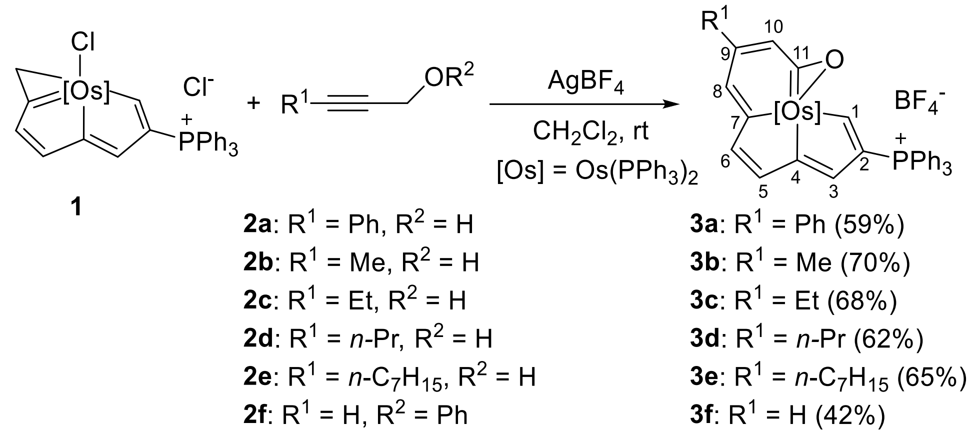

Synthesis and characterization of complex 3a: To a solution of complex **1** (200 mg, 0.17 mmol) and $AgBF_4$ (102 mg, 0.52 mmol) in dichloromethane (5 mL) was added 3-phenyl-2-propyn-1-ol (108 μL, 0.87 mmol). The reaction mixture was stirred at room temperature for 24 hours to give a green solution. Then the solution was evaporated under vacuum to a volume of approximately 2 mL. The residue was purified by column chromatography (SiO_2, 200-300 mesh, elute: dichloromethane/acetone = 20/1) to give **3a** as a green solid. Yield: 130 mg, 59%. ^{1}H NMR (600 MHz, $CDCl_3$, ppm) δ: 12.29 (d, 1H, *J*(PH) = 23.3 Hz, C1*H*), 7.83 (d, 1H, *J*(PH) = 4.4 Hz, C5*H*), 7.70 (d, 1H, *J*(PH) = 3.8 Hz, C3*H*), 7.52 (m, 1H, C6*H*), 7.19 (d, 1H, *J*(PH) = 3.4 Hz, C8*H*), 5.19 (s, 1H, C10*H*), and 7.75-6.71 (50H, other aromatic protons). ^{31}P{^{1}H} NMR (162 MHz, $CDCl_3$, ppm) δ: 12.55 (t, *J*(PP) = 6.0 Hz, C*P*Ph$_3$), and -9.39 (d, *J*(PP) = 6.0 Hz, Os*P*Ph$_3$). ^{13}C{^{1}H} NMR (151 MHz, $CDCl_3$, plus ^{13}C-DEPT 135, ^{1}H-^{13}C HSQC and ^{1}H-^{13}C HMBC, ppm) δ: 210.96 (t, *J*(PC) = 5.4 Hz, *C*7), 208.39 (m, *C*1), 203.99 (m, *C*11), 191.92 (dt, *J*(PC) = 23.5 Hz, *J*(PC) = 3.0 Hz, *C*4), 159.58 (s, *C*6), 151.72 (s, *C*5), 141.91 (s, *C*9), 131.15 (d, *J*(PC) = 24.5 Hz, *C*3), 123.65 (s, *C*8), 120.35 (d, *J*(PC) = 87.6 Hz, *C*2), 81.31 (s, *C*10), and 163.29-125.03 (other aromatic carbon atoms). HRMS (ESI): (m/z) calcd for **3a** $[C_{71}H_{56}OOsP_3]^+$ 1209.3153, found 1209.3129.

Synthesis and characterization of complex 3b: To a solution of complex **1** (200 mg, 0.17 mmol) and $AgBF_4$ (102 mg, 0. 52 mmol) in dichloromethane (5 mL) was added 2-butyn-1-ol (65 μL, 0.87 mmol). The reaction mixture was stirred at room temperature

for 24 hours to give a green solution. Then the solution was evaporated under vacuum to a volume of approximately 2 mL. The residue was purified by column chromatography (SiO_2, 200-300 mesh, elute: dichloromethane/acetone = 20/1) to give **3b** as a green solid. Yield: 146 mg, 70%. ^{1}H NMR (600 MHz, $CDCl_3$, ppm) δ: 12.26 (d, 1H, *J*(PH) = 22.4 Hz, C1*H*), 7.79 (m, 1H, C5*H*), 7.63 (m, 1H, C3*H*), 7.43 (m, 1H, C6*H*), 6.96 (s, 1H, C8*H*), 4.73 (s, 1H, C10*H*), 1.76 (s, 3H, CH_3), and 7.48-6.78 (45H, other aromatic protons). ^{31}P{^{1}H} NMR (243 MHz, $CDCl_3$, ppm) δ: 12.31 (s, C*P*Ph_3), and -9.45 (s, Os*P*Ph_3). ^{13}C{^{1}H} NMR (151 MHz, $CDCl_3$, plus ^{13}C-DEPT 135, ^{1}H-^{13}C HSQC and ^{1}H-^{13}C HMBC, ppm) δ: 212.08 (t, *J*(PC) = 5.3 Hz, *C*7), 207.94 (m, *C*1), 203.47 (m, *C*11), 191.18 (m, *C*4), 165.99 (s, *C*9), 158.06 (s, *C*6), 151.57 (s, *C*5), 130.54 (d, *J*(PC) = 24.7 Hz, *C*3), 125.33 (s, *C*8), 120.44 (d, *J*(PC) = 87.1 Hz, *C*2), 83.71 (s, *C*10), 25.28 (s, *C*H_3), and 134.38-126.43 (other aromatic carbon atoms). HRMS (ESI): (m/z) calcd for **3b** $[C_{66}H_{54}OOsP_3]^+$ 1147.2997, found 1147.2977.

Synthesis and characterization of complex 3c: To a solution of complex **1** (200 mg, 0.17 mmol) and $AgBF_4$ (102 mg, 0. 52 mmol) in dichloromethane (5 mL) was added 2-pentyn-1-ol (80 μL, 0.87 mmol). The reaction mixture was stirred at room temperature for 24 hours to give a green solution. Then the solution was evaporated under vacuum to a volume of approximately 2 mL. The residue was purified by column chromatography (SiO_2, 200-300 mesh, elute: dichloromethane/acetone = 20/1) to give **3c** as a green solid. Yield: 144 mg, 68%. ^{1}H NMR (600 MHz, $CDCl_3$, ppm) δ: 12.23 (d, *J*(PH) = 23.2 Hz, C1*H*), 7.74 (d, 1H, *J*(PH) = 4.9 Hz, C5*H*), 7.58 (d, 1H, *J*(PH) = 4.0 Hz, C3*H*), 7.44 (m, 1H, C6*H*), 6.89 (s, 1H, C8*H*), 4.91 (s, 1H, C10*H*), 2.13 (q, 2H, *J*(HH) = 7.6 Hz, C$H_2$$CH_3$), 1.10 (t, 3H, *J*(HH) = 7.6 Hz, CH_2CH_3, and 7.81-6.77 (45H, other aromatic protons). ^{31}P{^{1}H} NMR (162 MHz, $CDCl_3$, ppm) δ: 12.22 (t, *J*(PP) = 5.5 Hz, C*P*Ph_3), and -9.46 (d, *J*(PP) = 5.5 Hz, Os*P*Ph_3). ^{13}C{^{1}H} NMR (151 MHz, $CDCl_3$, plus ^{13}C-DEPT 135, ^{1}H-^{13}C HSQC and ^{1}H-^{13}C HMBC, ppm) δ: 212.20 (t, *J*(PC) = 5.3 Hz, *C*7), 207.79 (m, *C*1), 202.29 (m, *C*11), 191.22 (dt, *J*(PC) = 23.4 Hz, *J*(PC) = 4.2 Hz, *C*4), 171.44 (s, *C*9), 158.22 (s, *C*6), 151.32 (s, *C*5), 130.29 (d, *J*(PC) = 24.6 Hz, *C*3), 124.51 (s, *C*8), 120.44 (d, *J*(PC) = 86.9 Hz, *C*2), 82.88 (s, *C*10), 31.83 (s, *C*$H_2$$CH_3$),

11.90 (s, $CH_2\mathit{C}H_3$), and 134.38-126.39 (other aromatic carbon atoms). HRMS (ESI): (m/z) calcd for **3c** $[C_{67}H_{56}OOsP_3]^+$ 1161.3153, found 1161.3143.

Synthesis and characterization of complex 3d: To a solution of complex **1** (200 mg, 0.17 mmol) and $AgBF_4$ (102 mg, 0. 52 mmol) in dichloromethane (5 mL) was added 2-hexyn-1-ol (96 μL, 0.87 mmol). The reaction mixture was stirred at room temperature for 24 hours to give a green solution. Then the solution was evaporated under vacuum to a volume of approximately 2 mL. The residue was purified by column chromatography (SiO_2, 200-300 mesh, elute: dichloromethane/acetone = 20/1) to give **3d** as a green solid. Yield: 133 mg, 62%. ^{1}H NMR (400 MHz, $CDCl_3$, ppm) δ: 12.21 (d, *J*(PH) = 24.0 Hz, C1*H*), 7.74 (d, 1H, *J*(PH) = 4.9 Hz, C5*H*), 7.60 (d, 1H, *J*(PH) = 3.7 Hz, C3*H*), 7.42 (m, 1H, C6*H*), 6.90 (s, 1H, C8*H*), 4.94 (s, 1H, C10*H*), 2.06 (t, 2H, *J*(HH) = 7.7 Hz, $C\mathit{H}_2CH_2CH_3$), 1.53 (m, 2H, $CH_2C\mathit{H}_2CH_3$), 1.00 (t, 3H, *J*(HH) = 7.3 Hz, $CH_2CH_2C\mathit{H}_3$), and 7.81-6.75 (45H, other aromatic protons). ^{31}P{^{1}H} NMR (162 MHz, $CDCl_3$, ppm) δ: 12.17 (s, C*P*Ph_3), and -9.32 (s, Os*P*Ph_3). ^{13}C{^{1}H} NMR (151 MHz, $CDCl_3$, plus ^{13}C-DEPT 135, ^{1}H-^{13}C HSQC and ^{1}H-^{13}C HMBC, ppm) δ: 211.85 (t, *J*(PC) = 5.2 Hz, *C*7), 207.79 (m, *C*1), 202.46 (m, *C*11), 191.10 (dt, *J*(PC) = 25.0 Hz, *J*(PC) = 3.2 Hz, *C*4), 170.18 (s, *C*9), 158.26 (s, *C*6), 151.29 (s, *C*5), 130.32 (d, *J*(PC) = 24.9 Hz, *C*3), 124.94 (s, *C*8), 120.41 (d, *J*(PC) = 87.4 Hz, *C*2), 83.43 (s, *C*10), 41.61 (s, $\mathit{C}H_2CH_2CH_3$), 21.43 (s, $CH_2\mathit{C}H_2CH_3$), 14.75 (s, $CH_2CH_2\mathit{C}H_3$), and 134.38-126.43 (other aromatic carbon atoms). HRMS (ESI): (m/z) calcd for **3d** $[C_{68}H_{58}OOsP_3]^+$ 1175.3310, found 1175.3295.

Synthesis and characterization of complex 3e: To a solution of complex **1** (200 mg, 0.17 mmol) and $AgBF_4$ (102 mg, 0. 52 mmol) in dichloromethane (5 mL) was added 2-decyn-1-ol (157 μL, 0.87 mmol). The reaction mixture was stirred at room temperature for 24 hours to give a green solution. Then the solution was evaporated under vacuum to a volume of approximately 2 mL. The residue was purified by column chromatography (SiO_2, 200-300 mesh, elute: dichloromethane/acetone = 20/1) to give **3e** as a green solid. Yield: 145 mg, 65%. ^{1}H NMR (600 MHz, $CDCl_3$, ppm) δ: 12.21 (d, 1H, *J*(PH) = 23.3 Hz, C1*H*), 7.72 (d, 1H, *J*(PH) = 4.7 Hz, C5*H*), 7.57 (d, 1H, *J*(PH) =

3.7 Hz, C3*H*), 7.41 (m, 1H, C6*H*), 6.89 (s, 1H, C8*H*), 4.93 (s, 1H, C10*H*), 2.08-0.87 (m, 15H, C_7H_{15}), and 7.68-6.76 (45H, other aromatic protons). $^{31}P\{^1H\}$ NMR (243 MHz, $CDCl_3$, ppm) δ: 12.16 (s, C*P*Ph_3), and -9.40 (s, Os*P*Ph_3). $^{13}C\{^1H\}$ NMR (151 MHz, $CDCl_3$, plus ^{13}C-DEPT 135, ^{1}H-^{13}C HSQC and ^{1}H-^{13}C HMBC, ppm) δ: 211.88 (t, *J*(PC) = 5.3 Hz, *C*7), 207.81 (m, *C*1), 202.43 (m, *C*11), 191.21 (dt, *J*(PC) = 25.1 Hz, *J*(PC) = 3.9 Hz, *C*4), 170.49 (s, *C*9), 158.24 (s, *C*6), 151.32 (s, *C*5), 130.35 (d, *J*(PC) = 24.8 Hz, *C*3), 124.90 (s, *C*8), 120.44 (d, *J*(PC) = 87.2 Hz, *C*2), 83.41 (s, *C*10), 39.45-14.16 (m, C_7H_{15}), and 134.40-127.11 (other aromatic carbon atoms). HRMS (ESI): (m/z) calcd for **3e** $[C_{72}H_{66}OOsP_3]^+$ 1231.3936, found 1231.3922.

Synthesis and characterization of complex 3f: To a solution of complex **1** (200 mg, 0.17 mmol) and $AgBF_4$ (102 mg, 0. 52 mmol) in wet dichloromethane (5 mL) was added phenyl propargyl ether (22 μL, 0.17 mmol). The reaction mixture was stirred at room temperature for 24 hours to give a green solution. Then the solution was evaporated under vacuum to a volume of approximately 2 mL. The residue was purified by column chromatography (SiO_2, 200-300 mesh, elute: dichloromethane/acetone = 20/1) to give **3f** as a green solid. Yield: 87 mg, 42%. ^{1}H NMR (600 MHz, $CDCl_3$, ppm) δ: 12.36 (d, 1H, *J*(PH) = 23.0 Hz, C1*H*), 7.88 (d, 1H, *J*(PH) = 4.8 Hz, C5*H*), 7.78 (d, 1H, *J*(PH) = 3.6 Hz, C3*H*), 7.53 (m, 1H, C9*H*), 7.52 (m, 1H, C6*H*), 6.98 (d, 1H, *J*(PH) = 4.7 Hz, C8*H*), 4.93 (d, 1H, *J*(HH) = 7.9 Hz, C10*H*), and 6.40-7.86 (45H, other aromatic protons). $^{31}P\{^1H\}$ NMR (243 MHz, $CDCl_3$, ppm) δ: 12.31 (s, C*P*Ph_3), and -10.62 (s, Os*P*Ph_3). $^{13}C\{^1H\}$ NMR (151 MHz, $CDCl_3$, plus ^{13}C-DEPT 135, ^{1}H-^{13}C HSQC and ^{1}H-^{13}C HMBC, ppm) δ: 211.47 (t, *J*(PC) = 5.3 Hz, *C*7), 207.59 (m, *C*1), 203.69 (m, *C*11), 191.90 (dt, *J*(PC) = 25.1 Hz, *J*(PC) = 2.9 Hz, *C*4), 158.98 (s, *C*6), 152.23 (s, *C*9), 151.88 (s, *C*5), 130.80 (d, *J*(PC) = 24.8 Hz, *C*3), 122.80 (s, *C*8), 120.33 (d, *J*(PC) = 87.2 Hz, *C*2), 81.21 (s, *C*10), and 134.48-114.87 (other aromatic carbon atoms). HRMS (ESI): (m/z) calcd for **3f** $[C_{65}H_{52}OOsP_3]^+$ 1133.2840, found 1133.2814.

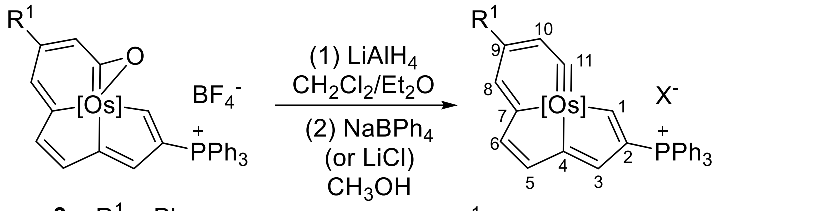

Synthesis and characterization of complex 4a: Under Ar atmosphere, **3a** (200 mg, 0.15 mmol) and $LiAlH_4$ (23 mg, 0.62 mmol) were added step by step to a mixed solution (5 mL) of dichloromethane and ethyl ether (v/v = 1:1). The reaction mixture was stirred at room temperature for 5 mins to give a brown solution, which was then filtered under an argon atmosphere and the filtrate was evaporated in vacuo. Then the residue was dissolved in methanol followed by the addition of a methanol solution of $NaBPh_4$ (132 mg, 0.39 mmol). After a few seconds, the mixture was filtered under Ar, and the precipitate was washed with methanol. The solid was dissolved in dichloromethane, then *n*-hexane was added to the solution. The precipitate was collected by filtration, washed with *n*-hexane and dried under vacuum to give **4a** a green solid. Yield: 172 mg, 71%. ^{1}H NMR (400 MHz, $CDCl_3$, ppm) δ: 11.37 (d, *J*(PH) = 23.5 Hz, 1H, C1*H*), 8.26 (m, 1H, C6*H*), 8.19 (s, 1H, C5*H*), 8.05 (s, 1H, C3*H*), 6.76 (m, 1H, C8*H*), 4.49 (m, 1H, C10*H*), and 7.68-6.54 (70H, other aromatic protons). ^{31}P{^{1}H} NMR (162 MHz, $CDCl_3$, ppm) δ: 12.79 (t, *J*(PP) = 7.2 Hz, C*P*Ph_3), and 1.51 (d, *J*(PP) = 7.2 Hz, Os*P*Ph_3). ^{13}C{^{1}H} NMR (101 MHz, $CDCl_3$, plus ^{13}C-DEPT 135, ^{1}H-^{13}C HSQC and ^{1}H-^{13}C HMBC, ppm) δ: 335.33 (m, *C*11), 218.85 (m, *C*7), 212.44 (m, *C*1), 202.33 (m, *C*4), 165.09-163.63 (BPh_4, αC), 159.09 (s, *C*5), 156.41 (s, *C*6), 140.65 (s, *C*9), 128.93 (d, *J*(PC) = 25.4 Hz, *C*3), 126.93 (s, *C*8), 120.62 (d, *J*(PC) = 86.4 Hz, *C*2),115.84 (s, *C*10), and 159.97-120.19 (other aromatic carbon atoms). HRMS (ESI): (m/z) calcd for **4a** $[C_{71}H_{56}OsP_3]^+$ 1193.3204, found 1193.3199.

Synthesis and characterization of complex 4b-BPh4: Under Ar atmosphere, **3b** (200 mg, 0.16 mmol) and $LiAlH_4$ (25 mg, 0.65 mmol) were added step by step to a mixed solution (5 mL) of dichloromethane and ethyl ether (v/v = 1:1). The reaction mixture was stirred at room temperature for 5 mins to give a brown solution, which was then

filtered under an argon atmosphere and evaporated in vacuo. Then the residue was dissolved in methanol followed by the addition of a methanol solution of $NaBPh_4$ (139 mg, 0.41 mmol). After a few seconds, the mixture was filtered under Ar, and the precipitate was washed with methanol. The solid was dissolved in dichloromethane, then *n*-hexane was added to the solution. The precipitate was collected by filtration, washed with *n*-hexane and dried under vacuum to give **4b-BPh₄** a green solid. Yield: 174 mg, 75%. ^{1}H NMR (400 MHz, $CDCl_3$, ppm) δ: 11.25 (d, *J*(PH) = 24.5 Hz, 1H, C1*H*), 8.14 (m, 1H, C6*H*), 8.03 (m, 1H, C5*H*), 7.94 (d, 1H, *J*(PH) = 2.8 Hz, C3*H*), 6.92 (s, 1H, C8*H*), 3.95 (m, 1H, C10*H*), 1.66 (s, 3H, CH_3), and 7.60-6.82 (65H, other aromatic protons). ^{31}P{^{1}H} NMR (243 MHz, $CDCl_3$, ppm) δ: 12.74 (t, *J*(PP) = 7.8 Hz, C*P*Ph₃), and 1.44 (d, *J*(PP) = 7.8 Hz, Os*P*Ph₃). ^{13}C{^{1}H} NMR (101 MHz, $CDCl_3$, plus ^{13}C-DEPT 135, ^{1}H-^{13}C HSQC and ^{1}H-^{13}C HMBC, ppm) δ: 334.78 (t, *J*(PC) = 16.2 Hz, *C*11), 219.69 (m, *C*7), 212.22 (m, *C*1), 201.83 (dt, *J*(PC) = 26.9 Hz, *C*4), 165.10-163.62 (BPh_4, αC), 163.18 (t, *J*(PC) = 2.6 Hz, *C*9), 157.63 (s, *C*5), 156.42 (s, *C*6), 128.70 (d, *J*(PC) = 21.6 Hz, *C*3), 128.01 (s, *C*8), 120.33 (d, *J*(PC) = 87.2 Hz, *C*2), 118.37 (s, *C*10), 23.63 (s, *C*H₃), and 136.40-121.59 (other aromatic carbon atoms). HRMS (ESI): (m/z) calcd for **4b-BPh₄** $[C_{66}H_{54}OsP_3]^+$ 1131.3048, found 1131.3026.

Synthesis and characterization of complex 4b-Cl: Under Ar atmosphere, **3b** (200 mg, 0.16 mmol) and $LiAlH_4$ (25 mg, 0.65 mmol) were added step by step to a mixed solution (5 mL) of dichloromethane and ethyl ether (v/v = 1:1). The reaction mixture was stirred at room temperature for 5 mins to give a brown solution, which was then filtered under an argon atmosphere and evaporated in vacuo. Then the residue was dissolved in methanol followed by the addition of a methanol solution of LiCl (17 mg, 0.40 mmol). After a few seconds, the mixture solution was evaporated in vacuo. Then the residue was dissolved in dichloromethane, after which *n*-hexane was added. The precipitate was collected by filtration, washed with *n*-hexane and dried under vacuum to give **4b-Cl** a green solid. Yield: 131 mg, 70%. ^{1}H NMR (600 MHz, $CDCl_3$, ppm) δ: 11.25 (dt, *J*(PH) = 24.6 Hz, *J*(PH) = 2.6 Hz, 1H, C1*H*), 8.16 (d, *J*(PH) = 4.8 Hz, 1H, C6*H*), 8.04 (m, 1H, C5*H*), 7.96 (d, *J*(PH) = 2.8 Hz, 1H, C3*H*), 6.93 (s, 1H, C8*H*), 3.96 (d, *J*(PH) =

2.5 Hz, 1H, C10*H*), 1.67 (s, 3H, CH_3), and 7.82-6.78 (45H, other aromatic protons). ^{31}P{^{1}H} NMR (243 MHz, $CDCl_3$, ppm) δ: 12.61 (t, *J*(PP) = 8.5 Hz, C*P*Ph_3), and 1.76 (d, *J*(PP) = 8.5 Hz, Os*P*Ph_3). ^{13}C{^{1}H} NMR (150.9 MHz, CD_2Cl_2, plus ^{13}C-DEPT 135, ^{1}H-^{13}C HSQC and ^{1}H-^{13}C HMBC, ppm) δ: 334.77 (t, *J*(PC) = 16.0 Hz, *C*11), 219.65 (m, *C*7), 212.27 (m, *C*1), 201.70 (dt, *J*(PC) = 26.8 Hz, *J*(PC) = 3.4 Hz, *C*4), 163.18 (s, *C*9), 157.60 (s, *C*5), 156.40 (s, *C*6), 128.64 (d, *J*(PC) = 26.5 Hz, *C*3), 128.00 (s, *C*8), 120.73 (d, *J*(PC) = 86.2 Hz, *C*2), 118.39 (s, *C*10), 23.60 (s, *C*H_3), and 134.43-120.44 (other aromatic carbon atoms). HRMS (ESI): (m/z) calcd for **4b-Cl** $[C_{66}H_{54}OsP_3]^+$ 1131.3048, found 1131.3044.

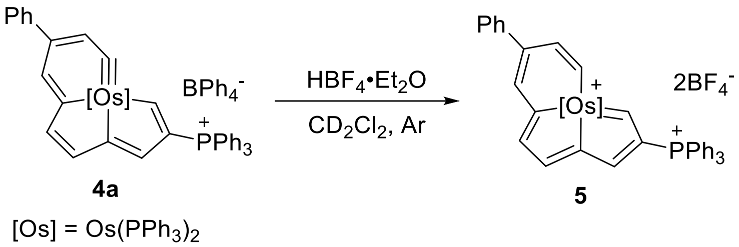

Synthesis and characterization of complex 5: Under an argon atmosphere, to a dichloromethane solution (0.5 mL) of **4a** (20 mg, 0.013 mmol) was added $HBF_4•Et_2O$ (7 mg, 0.04 mmol). The reaction mixture was stirred at room temperature for 5 mins to give a green solution. $^{31}P\{^{1}H\}$ NMR (243 MHz, CD_2Cl_2, ppm) δ: 16.97 (t, *J*(PP) = 5.7 Hz, C*P*Ph3), and 0.19 (d, *J*(PP) = 5.7 Hz, Os*P*Ph3). HRMS (ESI): (m/z) calcd for **5** $[C_{71}H_{57}OsP_3]^{2+}$ 597.1639, found 597.1662.

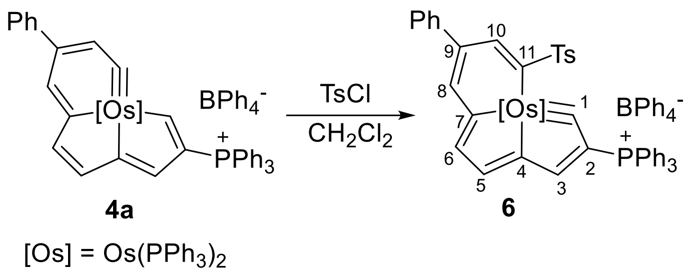

Synthesis and characterization of complex 6: Under an argon atmosphere, to a dichloromethane solution (5 mL) of **4a** (200 mg, 0.13 mmol) was added tosyl chloride (126 mg, 0.66 mmol). The reaction mixture was stirred at room temperature for 12 hours to give a green solution. Then the solution was filtered through a Celite pad, and evaporated under vacuum to a volume of approximately 2 mL. The residue was purified by column chromatography (SiO_2, 200-300 mesh, elute: dichloromethane) to give **6** as a green solid. Yield: 130 mg, 60%. ^{1}H NMR (600 MHz, $CDCl_3$, ppm) δ: 7.63 (s, 1H, C5*H*), 7.58 (s, 1H, C8*H*), 7.50 (s, 1H, C6*H*), 7.21 (s, 1H, C3*H*), 6.15 (s, 1H, C10*H*), 2.35 (s, 3H, CH_3), and 6.68-7.67 (74H, other aromatic proton). ^{31}P{^{1}H} NMR (243 MHz, $CDCl_3$, ppm) δ: 6.09 (t, *J*(PP) = 6.6 Hz, C*P*Ph_3), -4.24 (d, *J*(PP) = 6.6 Hz, Os*P*Ph_3). ^{13}C{^{1}H} NMR (151 MHz, $CDCl_3$, plus ^{13}C-DEPT 135, ^{1}H-^{13}C HSQC and ^{1}H-^{13}C HMBC, ppm) δ: 323.41 (td, *J*(PC) = 15.9 Hz, *J*(PC) = 10.6 Hz, *C*1), 222.91 (t, *J* = 10.4 Hz, *C*7), 201.91 (t, *J*(PC) = 12.0 Hz, *C*11), 189.31 (d, *J*(PC) = 29.1 Hz, *C*4), 164.79-163.81 (BPh_4, αC), 160.64 (s, *C*6), 159.21 (s, *C*5), 143.05 (m, *C*3), 142.72 (s, *C*9), 135.83 (s, *C*8), 134.85 (s, *C*10), 121.18 (d, *J*(PC) = 90.0 Hz, *C*2), 21.48 (s, *C*H_3), and 151.09-122.88 (other aromatic carbon atoms). HRMS (ESI): (m/z) calcd for **6** $[C_{78}H_{62}O_2OsP_3S]^+$ 1347.3293, found 1347.3295.

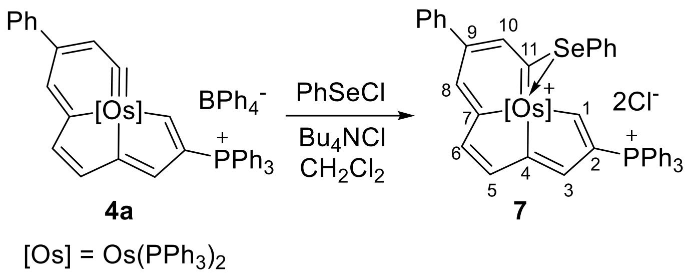

Synthesis and characterization of complex 7: Under an argon atmosphere, to a dichloromethane solution (5 mL) of **4a** (200 mg, 0.13 mmol) was added PhSeCl (127 mg, 0.66 mmol) and TBACl (110mg, 0.39 mmol). The reaction mixture was stirred at room temperature for 5 hours to give a green solution. Then the solution was evaporated under vacuum to a volume of approximately 2 mL. The residue was purified by column chromatography (SiO_2, 200-300 mesh, elute: dichloromethane/methanol = 10/1) to give **7** as a green solid. Yield: 118 mg, 64%. ^{1}H NMR (600 MHz, CD_2Cl_2, ppm) δ: 16.05 (d, *J*(PH) = 17.4 Hz, 1H, C1*H*), 8.40 (s, 1H, C3*H*), 8.22 (s, 1H, C10*H*), 8.06 (s, 1H, C8*H*), 7.84 (s, 1H, C5*H*), 7.66 (s, 1H, C6*H*), 8.13-6.72 (55H, other aromatic protons). ^{31}P{^{1}H} NMR (243 MHz, CD_2Cl_2, ppm) δ: 11.44 (t, *J*(PP) = 5.4 Hz, C*P*Ph$_3$), -9.30 (dd, *J*(PP) = 226.0 Hz, *J*(PP) = 5.4 Hz, Os*P*Ph$_3$), and -14.39 (dd, *J*(PP) = 226.0 Hz, *J*(PP) = 5.4 Hz, Os*P*Ph$_3$). ^{13}C{^{1}H} NMR (151 MHz, CD_2Cl_2, plus ^{13}C-DEPT 135, ^{1}H-^{13}C HSQC and ^{1}H-^{13}C HMBC, ppm) δ: 215.84 (t, *J*(PC) = 7.2 Hz, *C*7), 213.79 (t, *J*(PC) = 7.1 Hz, *C*11), 207.82 (t, *J*(PC) = 9.3 Hz, *C*1), 194.60 (m, *C*4), 163.57 (s, *C*5), 162.48 (s, *C*6), 142.63 (d, *J*(PC) = 23.2 Hz, *C*3), 139.02 (s, *C*9), 136.22 (m, *C*2), 131.09 (s, *C*8), 130.00 (s, *C*10), and 156.01-118.70 (other aromatic carbon atoms). HRMS (ESI): (m/z) calcd for **7** [$C_{77}H_{61}OsP_3Se$]$^{2+}$ 675.1378, found 675.1385.

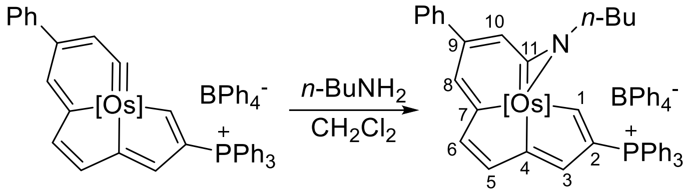

4a **8**

[Os] = $Os(PPh_3)_2$

Synthesis and characterization of complex 8: To a dichloromethane solution (5 mL) of **4a** (200 mg, 0.13 mmol) was added *n*-butylamine (48 mg, 0.66 mmol) in air. The reaction mixture was stirred at room temperature for 12 hours to give a blue solution. Then the solution was evaporated under vacuum to a volume of approximately 2 mL. The residue was purified by column chromatography (SiO_2, 200-300 mesh, elute: dichloromethane) to give **8** as a blue solid. Yield: 160 mg, 78%. ^{1}H NMR (600 MHz, $CDCl_3$, ppm) δ: 12.09 (d, *J*(PH) = 23.1 Hz, 1H, C1*H*), 7.65 (s, 1H, C3*H*), 7.27 (s, 1H, C5*H*), 7.21 (s, 1H, C8*H*), 7.08(s, 1H, C6*H*), 5.12 (s, 1H, C10*H*), 4.06 (m, 2H, $CH_2CH_2CH_2CH_3$), 1.12 (m, 2H, $CH_2CH_2CH_2CH_3$), 1.03 (m, 2H, $CH_2CH_2CH_2CH_3$), 0.74 (t, *J*(HH) = 7.1 Hz, 3H, $CH_2CH_2CH_2CH_3$), and 7.67-6.77 (50H, other aromatic protons). ^{31}P{^{1}H} NMR (243 MHz, $CDCl_3$, ppm) δ: 11.11 (s, C*P*Ph_3), and -5.30 (d, *J*(PP) = 3.5 Hz, Os*P*Ph_3). ^{13}C{^{1}H} NMR (101 MHz, $CDCl_3$, plus ^{13}C-DEPT 135, ^{1}H-^{13}C HSQC and ^{1}H-^{13}C HMBC, ppm) δ: 201.09 (m, *C*1), 196.57 (dt, *J*(PC) = 10.0 Hz, *J*(PC) = 4.8 Hz, *C*4), 194.35 (m, *C*7), 165.08-163.60 (BPh_4, αC), 158.83 (s, *C*6), 156.55 (t, *J*(PC) = 6.5 Hz, *C*11), 148.36 (s, *C*5), 143.50 (s, *C*9), 131.46 (d, *J*(PC) = 10.0 Hz, *C*3), 123.80 (s, *C*8), 121.06 (d, *J*(PC) = 87.0 Hz, *C*2), 84.70 (s, *C*10), 46.33 (s, $CH_2CH_2CH_2CH_3$), 31.81 (s, $CH_2CH_2CH_2CH_3$), 20.66 (s, $CH_2CH_2CH_2CH_3$), 13.81 (s, $CH_2CH_2CH_2CH_3$), and 136.40-121.64 (other aromatic carbon atoms). HRMS (ESI): (m/z) calcd for **8** $[C_{75}H_{65}NOsP_3]^+$ 1264.3940, found 1264.3937.

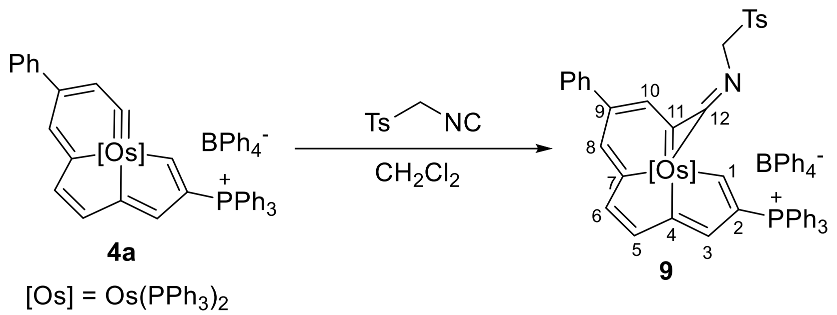

Synthesis and characterization of complex 9: Under an argon atmosphere, to a dichloromethane solution (5 mL) of **4a** (200 mg, 0.13 mmol) was added tosylmethyl isocyanide (129.16 mg, 0.66 mmol). The reaction mixture was stirred at 50 °C for 3 days to give a yellow solution. Then the solution was evaporated under vacuum to a volume of approximately 2 mL. The residue was purified by column chromatography (SiO_2, 200-300 mesh, elute: dichloromethane/acetone = 50/1) to give **9** as a yellow solid. Yield: 95 mg, 43%. ^{1}H NMR (600 MHz, CD_2Cl_2, ppm) *δ*: 12.63 (d, *J*(PH) = 19.2 Hz, 1H, C1*H*), 8.70 (s, 1H, C3*H*), 8.18 (s, 1H, C6*H*), 8.16 (s, 1H, C5*H*), 8.03 (s, 1H, C8*H*), 7.69 (br s, 1H, C10*H*), 4.78 (s, 2H, CH_2), 2.54 (s, 3H, CH_3), and 7.87-6.55 (74H, other aromatic protons). ^{31}P{^{1}H} NMR (243 MHz, CD_2Cl_2, ppm) *δ*: 11.82 (s, C*P*Ph_3), and -9.14 (s, Os*P*Ph_3). ^{13}C{^{1}H} NMR (151 MHz, CD_2Cl_2, plus ^{13}C-DEPT 135, ^{1}H-^{13}C HSQC and ^{1}H-^{13}C HMBC, ppm) *δ*: 234.03 (m, *C*7), 207.61 (t, *J*(PC) = 10.2 Hz, *C*1), 204.24 (m, *C*4), 183.07 (m, *C*11), 178.59 (m, *C*12), 165.62 (s, *C*5), 165.26 (s, *C*6), 164.60-163.62 (BPh_4, αC), 144.53 (s, *C*9), 138.65 (d, *J*(PC) = 25.2 Hz, *C*3), 129.06 (s, *C*8), 120.35 (d, *J*(PC) = 87.3 Hz, *C*2), 113.25 (s, *C*10), 81.25 (s, *C*H_2), 22.72 (s, *C*H_3), and 159.14-121.76 (other aromatic carbon atoms). HRMS (ESI): (m/z) calcd for **9** $[C_{80}H_{65}NO_2OsP_3S]^+$ 1388.3558, found 1388.3566.

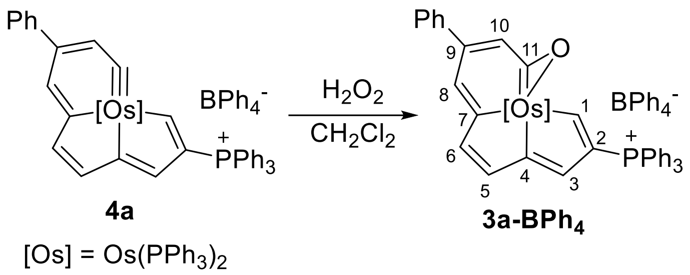

Synthesis and characterization of complex 3a-BPh4: To a dichloromethane solution (5 mL) of **4a** (200 mg, 0.13 mmol) was added H_2O_2 (30 % aq.) (74 mg, 0.65 mmol) in air. The reaction mixture was stirred at room temperature for 5 mins to give a green solution. Then the solution was evaporated under vacuum to a volume of approximately 2 mL. The residue was purified by column chromatography (SiO_2, 200-300 mesh, elute: dichloromethane) to give **3a-BPh4** as a green solid. Yield: 175 mg, 88%. ^{1}H NMR (600 MHz, $CDCl_3$, ppm) δ: 12.26 (d, 1H, *J*(PH) = 22.8 Hz, C1*H*), 7.78 (d, 1H, *J*(PH) = 4.7 Hz, C5*H*), 7.64 (d, 1H, *J*(PH) = 3.6 Hz, C3*H*), 7.52 (m, 1H, C6*H*), 7.20 (m, 1H, C8*H*), 5.19 (s, 1H, C10*H*), and 7.62-6.68 (70H, other aromatic proton). ^{31}P{^{1}H} NMR (243 MHz, $CDCl_3$, ppm) δ: 12.53 (s, C*P*Ph3), and -9.37 (s, Os*P*Ph3). ^{13}C{^{1}H} NMR (151 MHz, $CDCl_3$, plus ^{13}C-DEPT 135, ^{1}H-^{13}C HSQC and ^{1}H-^{13}C HMBC, ppm) δ: 211.03 (t, *J*(PC) = 5.2 Hz, *C*7), 208.43 (m, *C*1), 203.89 (m, *C*11), 191.96 (dt, *J*(PC) = 23.4 Hz, *J*(PC) = 3.2 Hz, *C*4), 164.86-163.88 (BPh_4, αC), 159.67 (s, *C*6), 151.60 (s, *C*5), 141.85 (s, *C*9), 130.90 (d, *J*(PC) = 24.6 Hz, *C*3), 120.31 (d, *J*(PC) = 87.7 Hz, *C*2), 81.37 (s, *C*10), and 163.47-121.58 (other aromatic carbon atoms). HRMS (ESI): (m/z) calcd for **3a-BPh4** $[C_{71}H_{56}OOsP_3]^+$ 1209.3153, found 1209.3149.

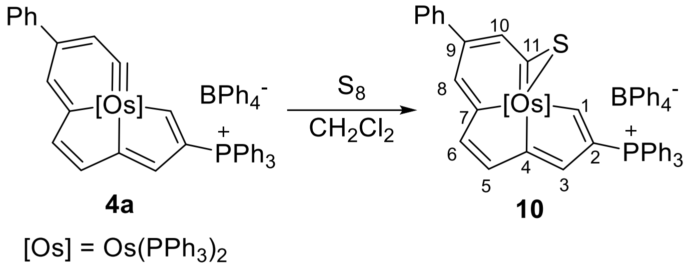

Synthesis and characterization of complex 10: Under an argon atmosphere, to a dichloromethane solution (5 mL) of **4a** (200 mg, 0.13 mmol) was added S_8 (339.4 mg, 1.32 mmol). The reaction mixture was stirred at room temperature for 12 hours to give a green solution. Then the solution was evaporated under vacuum to a volume of approximately 2 mL. The residue was purified by column chromatography (SiO_2, 200-300 mesh, elute: dichloromethane) to give **10** as a green solid. Yield: 155 mg, 77%. ^{1}H NMR (600 MHz, $CDCl_3$, ppm) δ: 13.12 (d, *J*(PH) = 20.9 Hz, 1H, C1*H*), 8.17 (s, 1H, C3*H*), 8.05 (s, 1H, C5*H*), 7.68 (s, 1H, C6*H*), 6.93 (s, 1H, C8*H*), 6.72 (s, 1H, C10*H*), and 7.70-6.63 (70H, other aromatic protons). ^{31}P{^{1}H} NMR (243 MHz, $CDCl_3$, ppm) δ: 11.84 (s, C*P*Ph$_3$), and -17.32 (s, Os*P*Ph$_3$). ^{13}C{^{1}H} NMR (151 MHz, $CDCl_3$, plus ^{13}C-DEPT 135, ^{1}H-^{13}C HSQC and ^{1}H-^{13}C HMBC, ppm) δ: 217.31(t, *J*(PC) = 5.0 Hz, *C*11), 207.55 (t, *J*(PC) = 7.4 Hz, *C*7), 198.82(dt, *J*(PC) = 25.2 Hz, *J*(PC) = 4.0 Hz, *C*4), 197.49 (t, *J*(PC) = 11.2 Hz, *C*1), 164.87-163.89 (BPh_4, αC), 163.39 (s, C6), 152.37 (s, *C*5), 142.40 (s, *C*9), 132.34 (d, *J*(PC) = 24.8 Hz, *C*3), 131.27 (d, *J*(PC) = 64.6 Hz, *C*2), 128.98 (s, *C*8), 112.26 (s, *C*10), and 153.51-120.00 (other aromatic carbon atoms). HRMS (ESI): (m/z) calcd for **10** $[C_{71}H_{56}OsP_3S]^+$ 1225.2925, found 1225.2935.

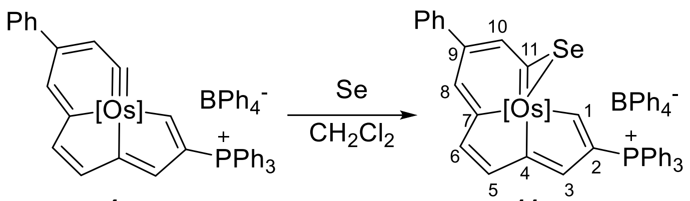

4a **11**

[Os] = $Os(PPh_3)_2$

Synthesis and characterization of complex 11: Under an argon atmosphere, to a dichloromethane solution (5 mL) of **4a** (200 mg, 0.13 mmol) was added Se (104 mg, 1.32 mmol). The reaction mixture was stirred at 40 °C for 24 hours to give a green solution. Then the solution was evaporated under vacuum to a volume of approximately 2 mL. The residue was purified by column chromatography (SiO_2, 200-300 mesh, elute: dichloromethane) to give **11** as a green solid. Yield: 153 mg, 74%. ^{1}H NMR (600 MHz, $CDCl_3$, ppm) δ: 13.58 (d, *J*(PH) = 20.7 Hz, 1H, C1*H*), 8.15 (s, 1H, C3*H*), 8.05 (d, *J*(PH) = 4.4 Hz, 1H, C5*H*), 7.60 (s, 1H, C6*H*), 6.91 (br, 1H, C8*H*), 6.82 (s, 1H, C10*H*), and 7.76–6.63 (70H, other aromatic protons). ^{31}P{^{1}H} NMR (243 MHz, $CDCl_3$, ppm) δ: 11.90 (s, C*P*Ph_3), and -21.20 (s, Os*P*Ph_3). ^{13}C{^{1}H} NMR (151 MHz, $CDCl_3$, plus ^{13}C-DEPT 135, ^{1}H-^{13}C HSQC and ^{1}H-^{13}C HMBC, ppm) δ: 232.43 (t, *J* (PC) = 6.1 Hz, *C*11), 206.82 (t, *J* (PC) = 7.5 Hz, *C*7), 199.11 (dt, *J* (PC) = 25.4 Hz, *J* (PC) = 3.5 Hz, *C*4), 196.73 (m, *C*1), 164.89-163.91 (BPh_4, αC), 163.48 (s, *C*6), 152.71 (s, *C*5), 151.12 (s, *C*9), 132.06 (d, *J* (PC) = 64.9 Hz, *C*2), 131.70 (d, *J*(PC) = 24.1 Hz, *C*3), 129.67 (s , *C*8), 122.20 (s, *C*10), and 142.08-119.95 (other aromatic carbon atoms). HRMS (ESI): (m/z) calcd for **11** $[C_{71}H_{56}OsP_3Se]^+$ 1273.2369, found 1273.2402.

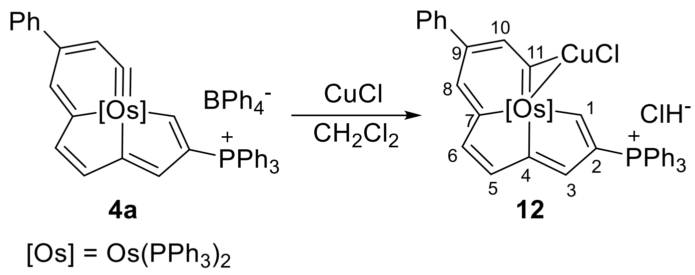

Synthesis and characterization of complex 12: Under an argon atmosphere, to a dichloromethane solution (5 mL) of **4a** (200 mg, 0.13 mmol) was added CuCl (65 mg, 0.66 mmol). The reaction mixture was stirred at room temperature for 5 hours to give a yellow solution. Then the solution was evaporated under vacuum to a volume of approximately 2 mL. The residue was purified by column chromatography (SiO_2, 200-300 mesh, elute: dichloromethane/methanol = 10/1) to give **12** as a yellow solid. Yield: 98 mg, 57%. ^{1}H NMR (600 MHz, $CDCl_3$, ppm) δ: 13.93 (d, J(PH) = 21.9 Hz, 1H, C1H), 7.99 (s, 1H, C3H), 7.55 (s, 1H, C5H), 7.38 (s, 1H, C6H), 6.90 (s, 1H, C8H), 6.18 (s, 1H, C10H), and 7.79-6.89 (70H, other aromatic protons). ^{31}P{^{1}H} NMR (243 MHz, $CDCl_3$, ppm) δ: 12.26 (s, CPPh$_3$), and -6.37 (s, OsPPh$_3$). ^{13}C{^{1}H} NMR (151 MHz, $CDCl_3$, plus ^{13}C-DEPT 135, ^{1}H-^{13}C HSQC and ^{1}H-^{13}C HMBC, ppm) δ: 312.60 (m, C11), 217.93 (m, C7), 212.32 (m, C1), 196.89 (m, C4), 159.47 (s, C5), 158.52(s, C6), 140.87 (s, C9), 131.10 (d, J(PC) = 23.2 Hz, C3), 127.13 (s, C8), 123.46 (s, C10), 120.26 (d, J(PC) = 86.8 Hz, C2), and 161.37-127.14 (other aromatic carbon atoms). HRMS (ESI): (m/z) calcd for **12** [$C_{71}H_{56}ClCuOsP_3$]$^+$ 1291.2189, found 1291.2164.

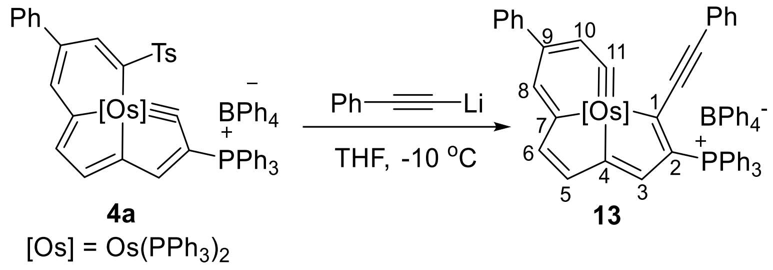

Synthesis and characterization of complex 13: Under an argon atmosphere, to a THF solution (5 mL) of **4a** (200 mg, 0.13 mmol) was added 300 μL lithium phenylacetylide (1.0 M in THF, 0.3 mmol). The reaction mixture was stirred at -10 °C for 10 mins to give a red solution. The reaction was quenched with saturated aqueous NH_4Cl (2 mL), and then warmed to room temperature. The resulting mixture was extracted with DCM (3 × 3 mL), Then the solution was concentrated to dryness. The residue was purified by column chromatography (SiO_2, 200-300 mesh, elute: dichloromethane) to give **13** as a red solid. Yield: 137 mg, 71%. ^{1}H NMR (600 MHz, $CDCl_3$, ppm) δ: 7.93 (br s, 1H, C5*H*), 7.58 (br s, 1H, C6*H*), 7.56 (s, 1H, C3*H*), 6.34 (s, 1H, C8*H*), 4.92 (s, 1H, C10*H*), and 7.39-6.53 (75H, other aromatic protons). $^{31}P\{^{1}H\}$ NMR (243 MHz, $CDCl_3$, ppm) δ: 8.65 (s, C*P*Ph_3), and -5.26 (s, Os*P*Ph_3). $^{13}C\{^{1}H\}$ NMR (151 MHz, $CDCl_3$, plus ^{13}C-DEPT 135, ^{1}H-^{13}C HSQC and ^{1}H-^{13}C HMBC, ppm) δ: 330.19 (t, *J*(PC) = 15.5 Hz, *C*11), 220.13 (s, *C*7), 203.83 (d, *J*(PC) = 26.2 Hz, *C*4), 188.10 (s, *C*1), 164.95-163.97 (BPh_4, αC), 158.80 (s, *C*6), 157.57 (s, *C*5), 139.98 (s, *C*9), 138.45 (m, *C*12), 135.58 (d, *J*(PC) = 25.0 Hz, *C*3), 125.67 (s, *C*8), 120.93 (d, *J*(PC) = 85.6 Hz, *C*2), 114.94 (s, *C*10), 104.54 (d, *J*(PC) = 13.2 Hz, *C*13), and 160.86-121.63 (other aromatic carbon atoms). HRMS (ESI): (m/z) calcd for **13** $[C_{79}H_{60}OsP_3]^+$ 1293.3518, found 1293.3523.

^{18}O-Labeling experiments

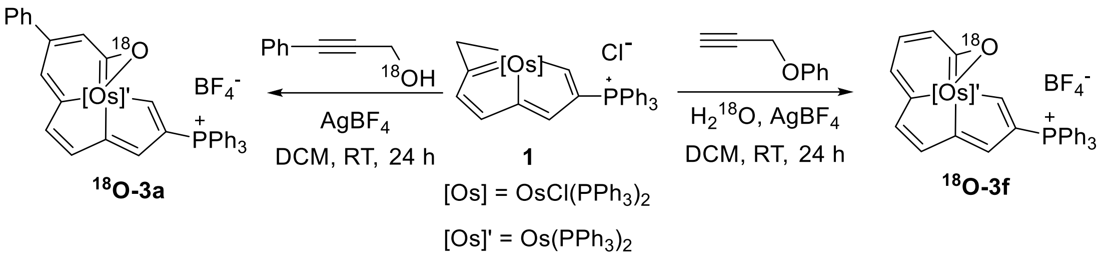

Supplementary Figure 1. ^{18}O-Labeling experiments for **^{18}O-3a** and **^{18}O-3f**.

Synthesis and characterization of complex ^{18}O-3a: To a solution of complex **1** (20 mg, 0.017 mmol) and $AgBF_4$ (10.2 mg, 0.052 mmol) in dichloromethane (5 mL) was added 3-phenyl-2-propyn-1-ol-^{18}O (10.8 μL, 0.087 mmol). The reaction mixture was stirred at room temperature for 24 hours to give a green solution. Then the solution was evaporated under vacuum to a volume of approximately 2 mL. The residue was purified by column chromatography (SiO_2, 200-300 mesh, elute: dichloromethane/acetone = 20/1) to give **^{18}O-3a** as a green solid. Yield: 11.0 mg, 50%. HRMS (ESI): (m/z) calcd for **^{18}O-3a** $[C_{71}H_{56}{}^{18}OOsP_3]^+$ 1211.3196, found 1211.3214.

Synthesis and characterization of complex ^{18}O-3f: To a solution of complex **1** (20 mg, 0.017 mmol) and $AgBF_4$ (10.2 mg, 0.052 mmol) in dichloromethane (5 mL) was added phenyl propargyl ether (2 μL, 0.017 mmol) and $H_2{}^{18}O$ (4.6 μL, 0.26 mmol). The reaction mixture was stirred at room temperature for 24 hours to give a green solution. Then the solution was evaporated under vacuum to a volume of approximately 2 mL. The residue was purified by column chromatography (SiO_2, 200-300 mesh, elute: dichloromethane/acetone = 20/1) to give **^{18}O-3f** as a green solid. Yield: 6 mg, 29%. HRMS (ESI): (m/z) calcd for **^{18}O-3f** $[C_{65}H_{52}{}^{18}OOsP_3]^+$ 1135.2883, found 1135.2885.

3. Proposed mechanism

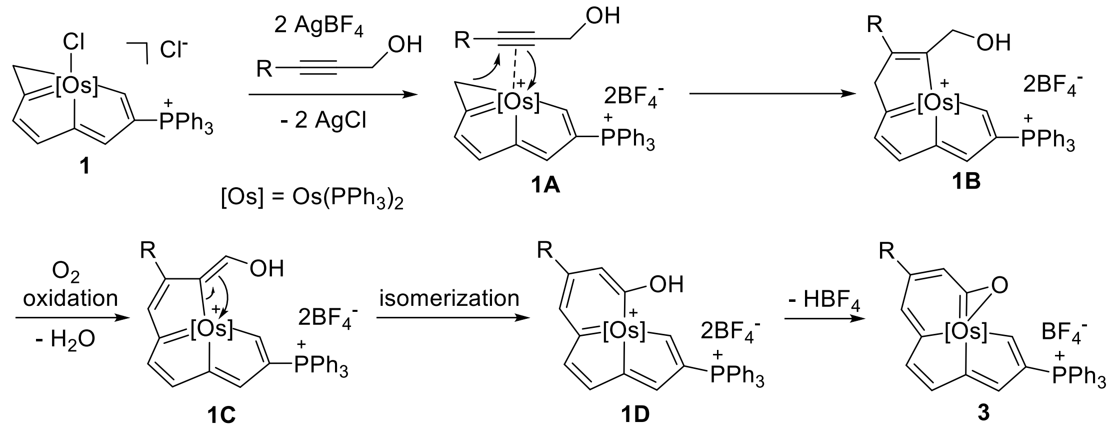

Supplementary Figure 2. Proposed mechanism of **1** with propargylic alcohols.

Initially, $AgBF_4$ removes the Cl ligand and Cl^- anion to generate an open site, which is then occupied by propargylic alcohol to produce intermediate **1A**. Afterwards, a formal [3+2] cycloaddition occurs, affording intermediate **1B**, which is subsequently oxidized by O_2 to form **1C**. **1C** undergoes isomerization to give **1D** driven by aromatization, which losses one molecule of HBF_4 to produce the final product. Notably, propargyl aldehydes were found to be unreactive with complex **1**, indicating the oxidation step must occur after the coordination step of the alkynyl to osmium.

4. Resonance structures of 4

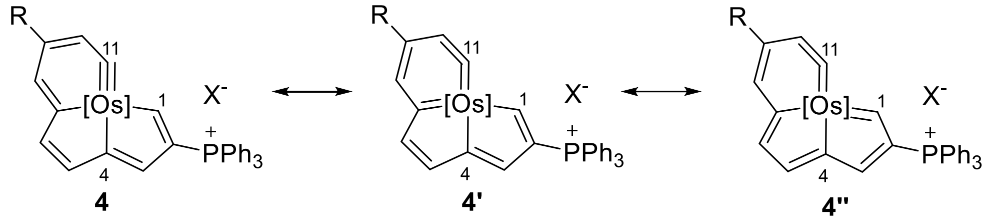

Supplementary Figure 3. Resonance structures of **4**.

5. NMR spectra

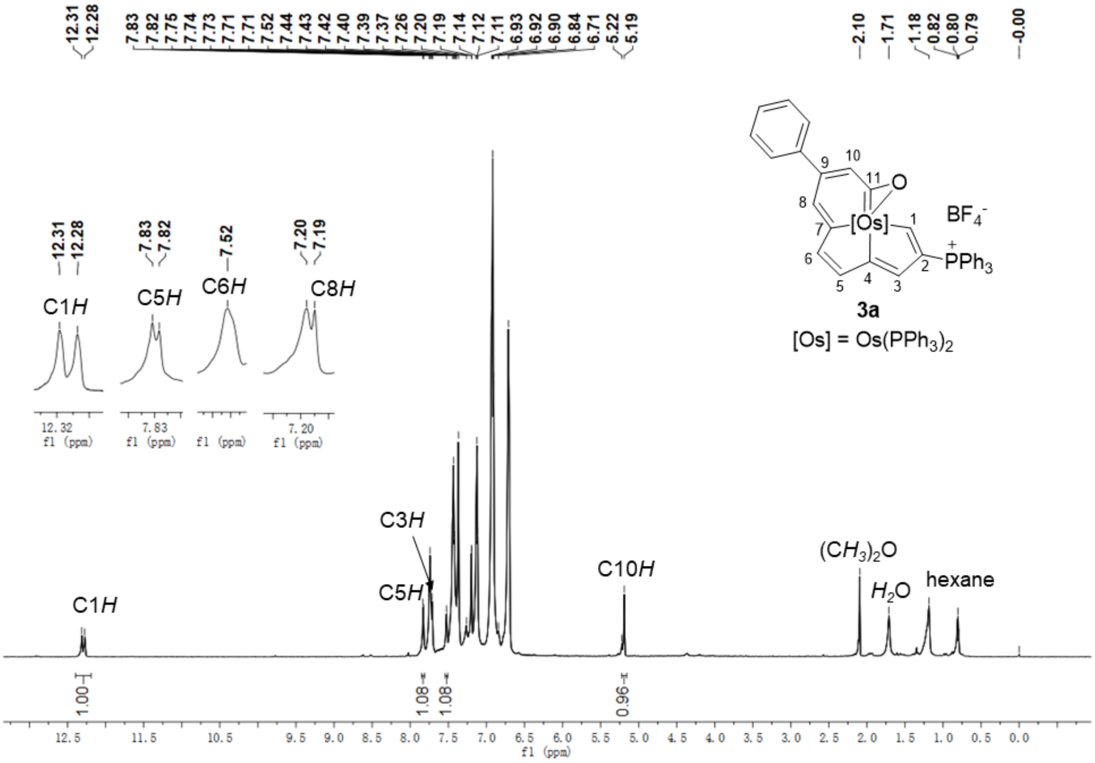

Supplementary Figure 4. The ^{1}H NMR (600 MHz, $CDCl_3$) spectrum for **3a**.

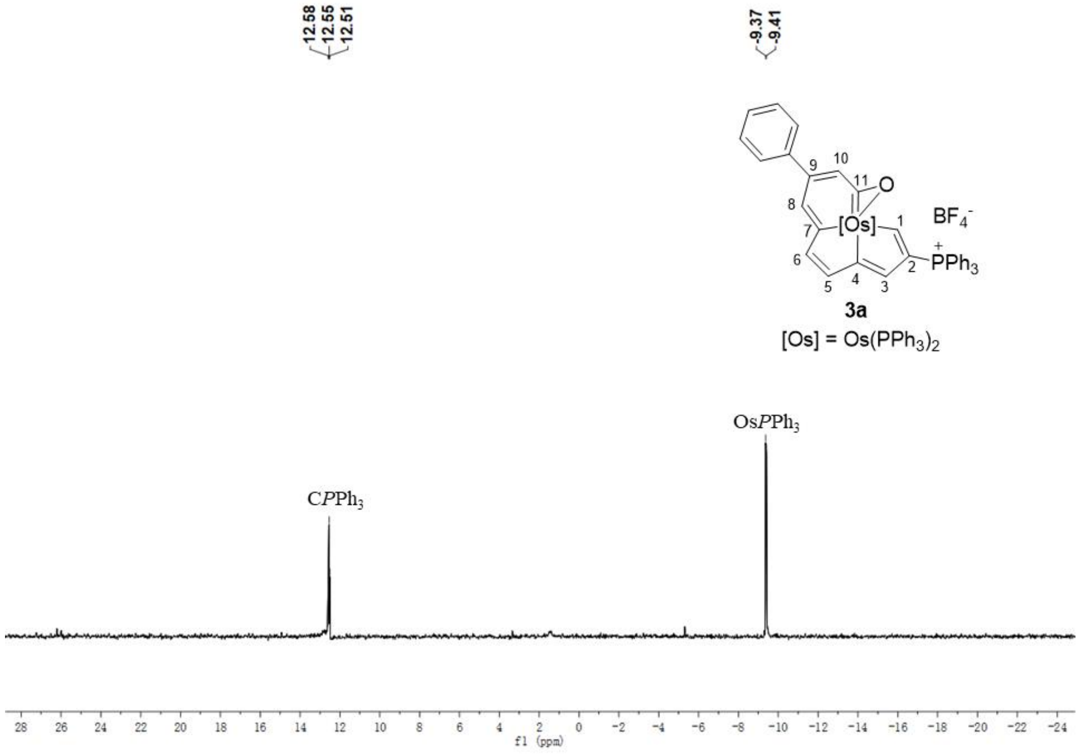

Supplementary Figure 5. The $^{31}P\{^{1}H\}$ NMR (243 MHz, $CDCl_3$) spectrum for **3a**.

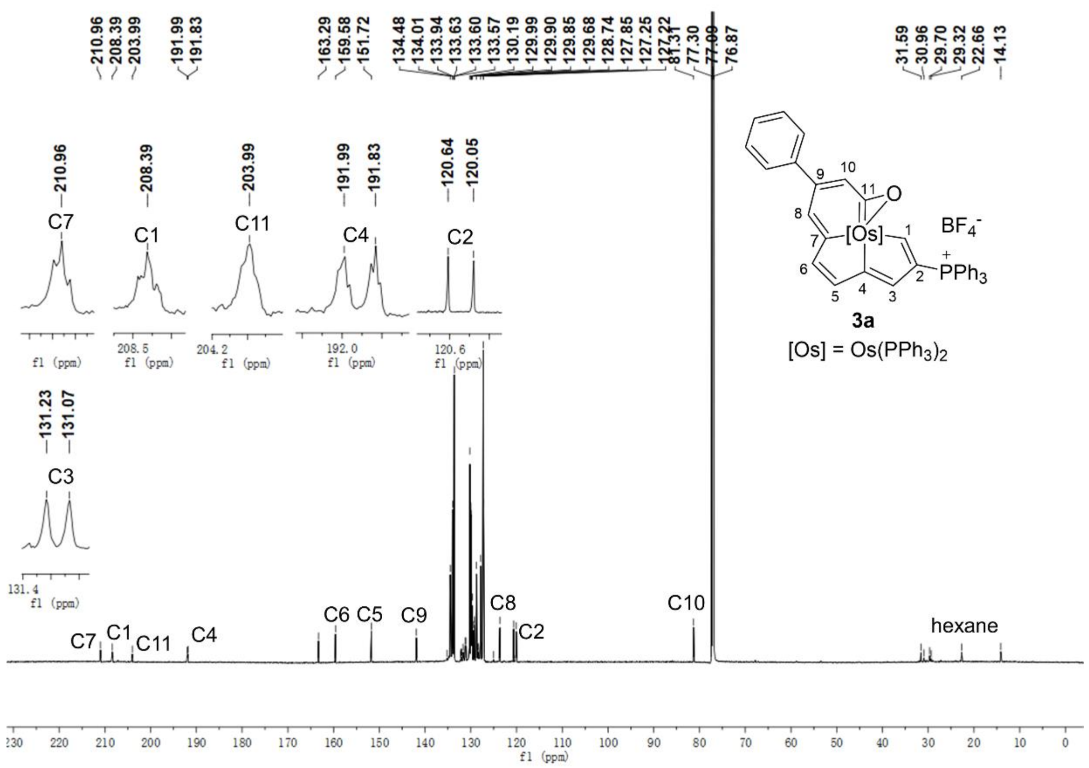

Supplementary Figure 6. The $^{13}C\{^{1}H\}$ NMR (151 MHz, $CDCl_3$) spectrum for **3a**.

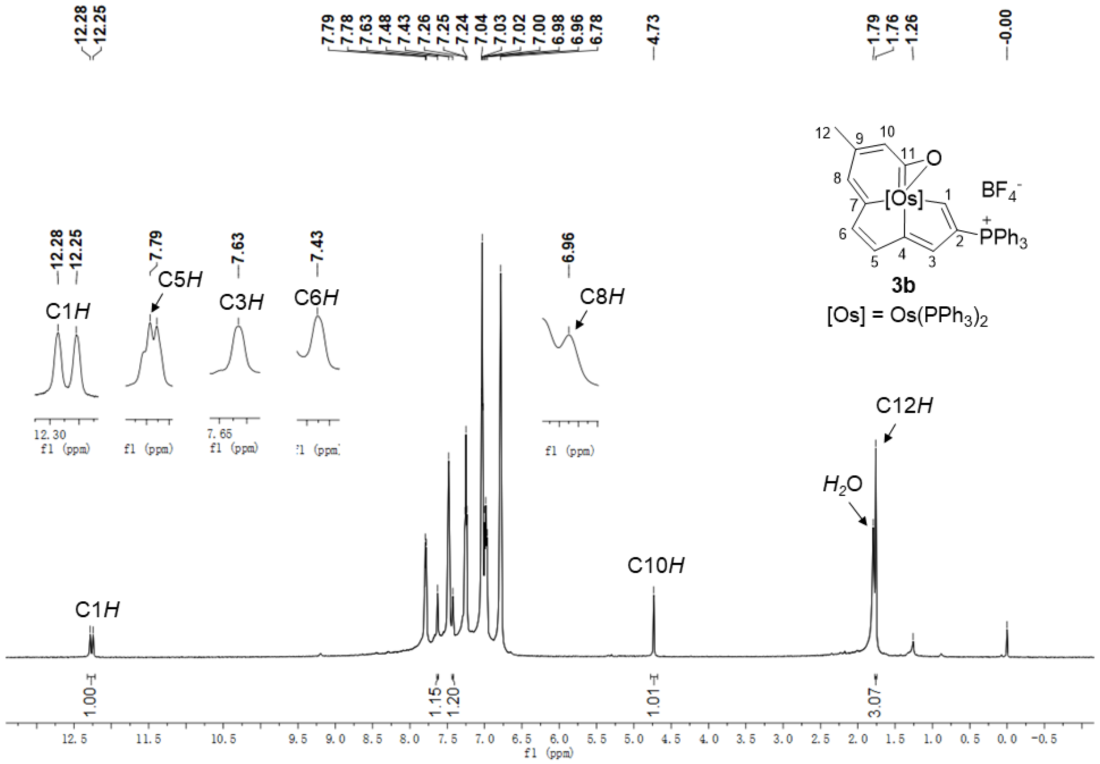

Supplementary Figure 7. The ^{1}H NMR (600 MHz, CDCl$_3$) spectrum for **3b**.

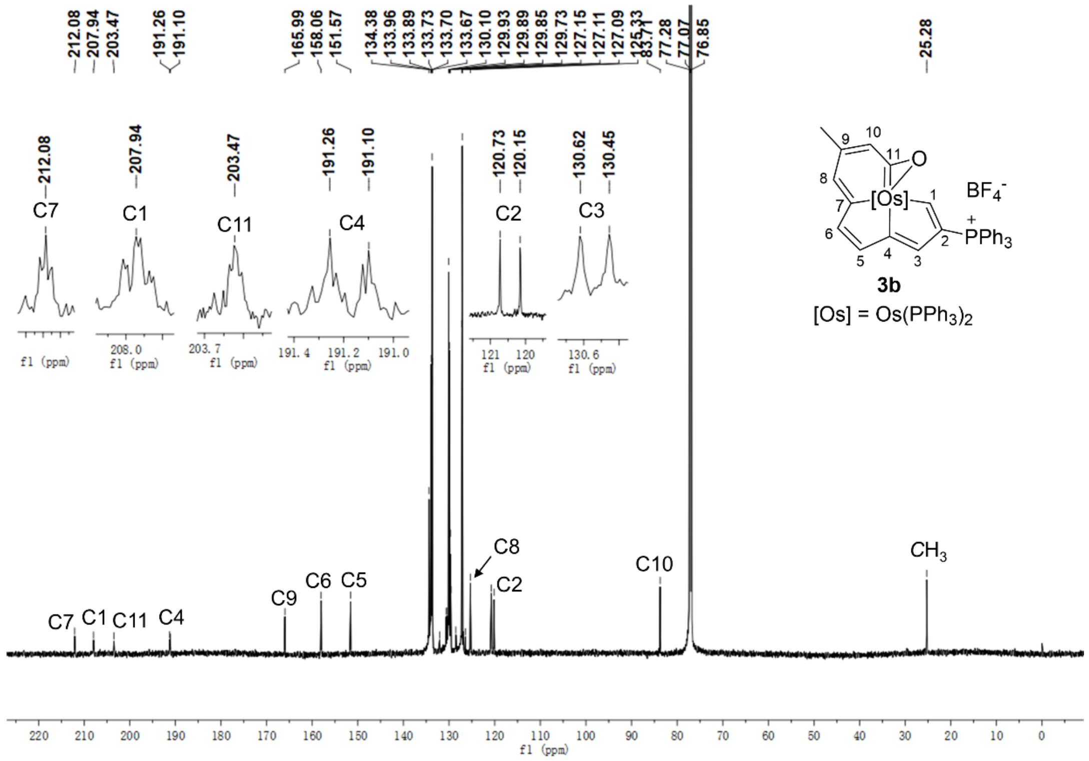

Supplementary Figure 8. The ^{31}P{^{1}H} NMR (243 MHz, CDCl$_3$) spectrum for **3b**.

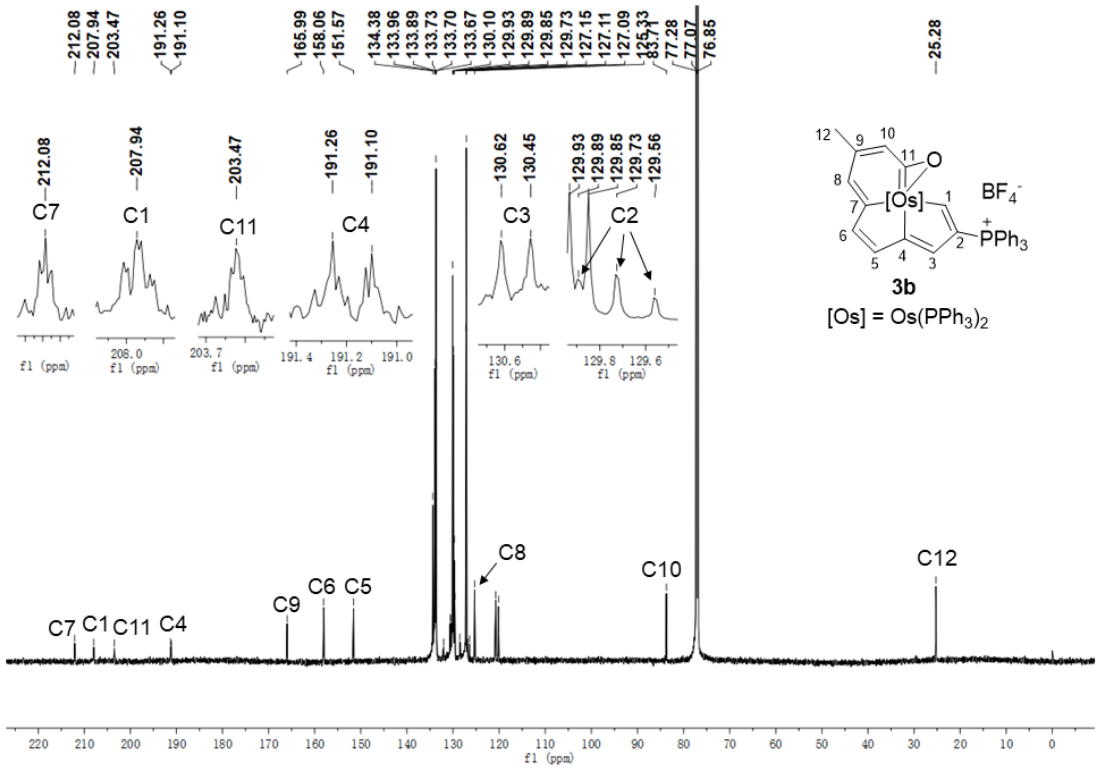

Supplementary Figure 9. The $^{13}C\{^1H\}$ NMR (151 MHz, $CDCl_3$) spectrum for **3b**.

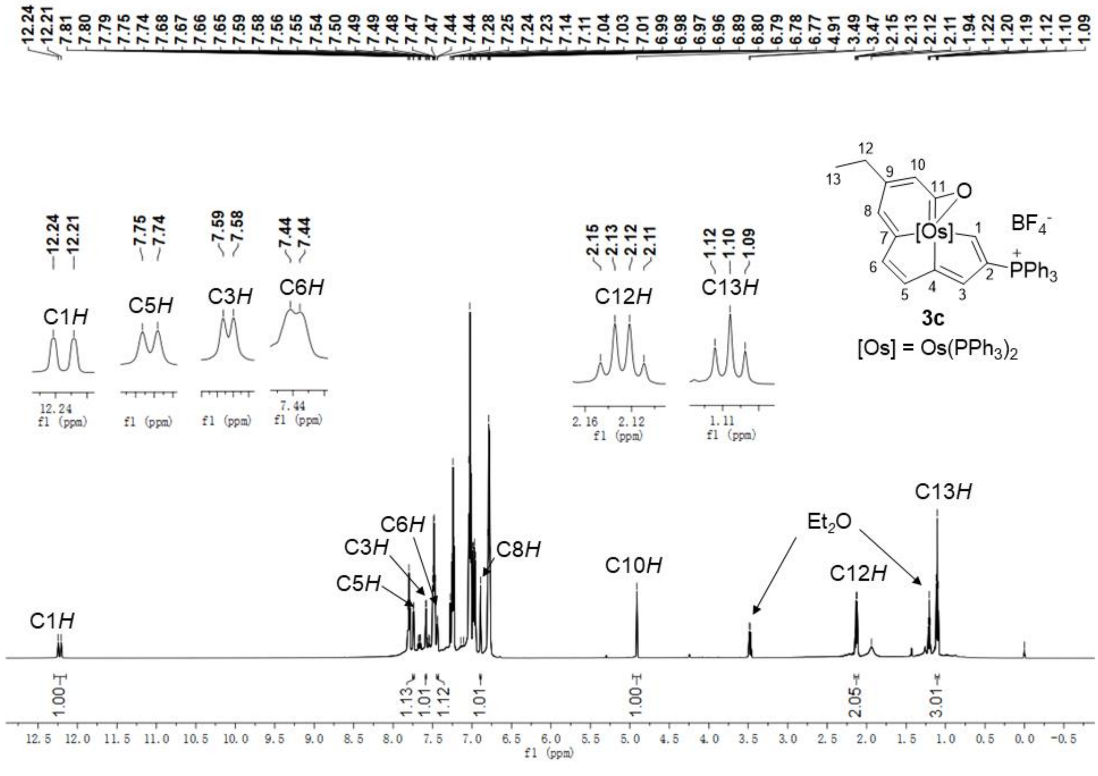

Supplementary Figure 10. The ^1H NMR (600 MHz, $CDCl_3$) spectrum for **3c**.

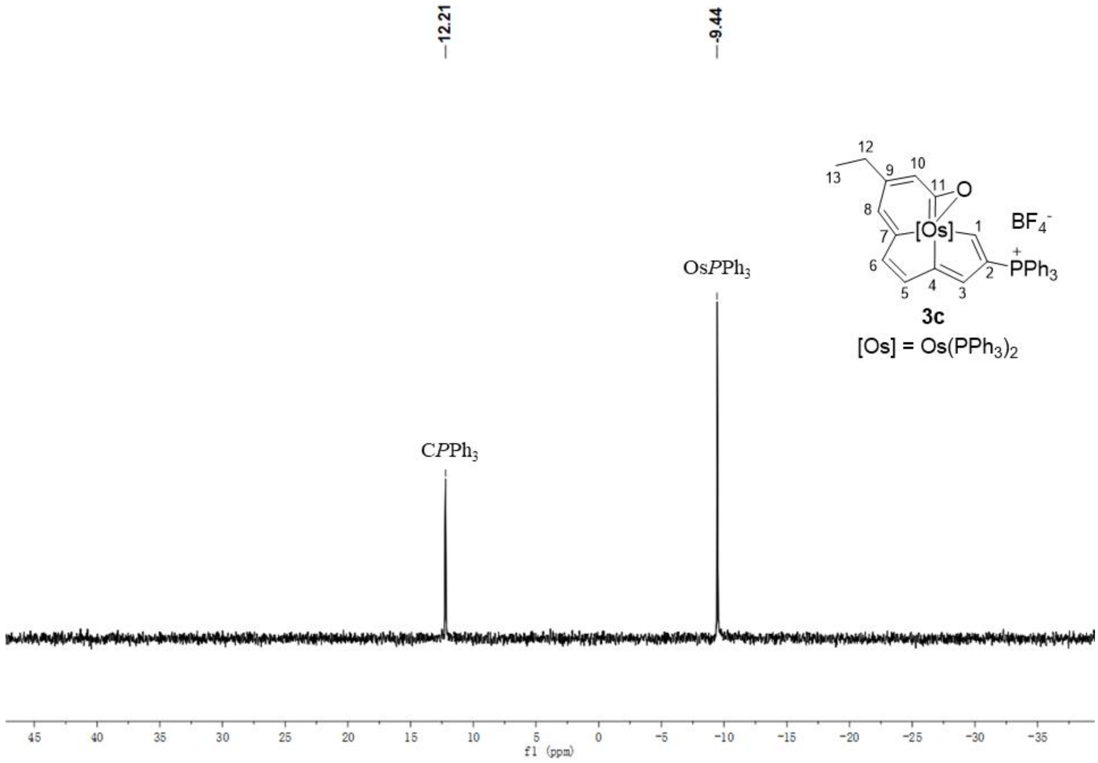

Supplementary Figure 11. The ^{31}P{^{1}H} NMR (162 MHz, $CDCl_3$) spectrum for **3c**.

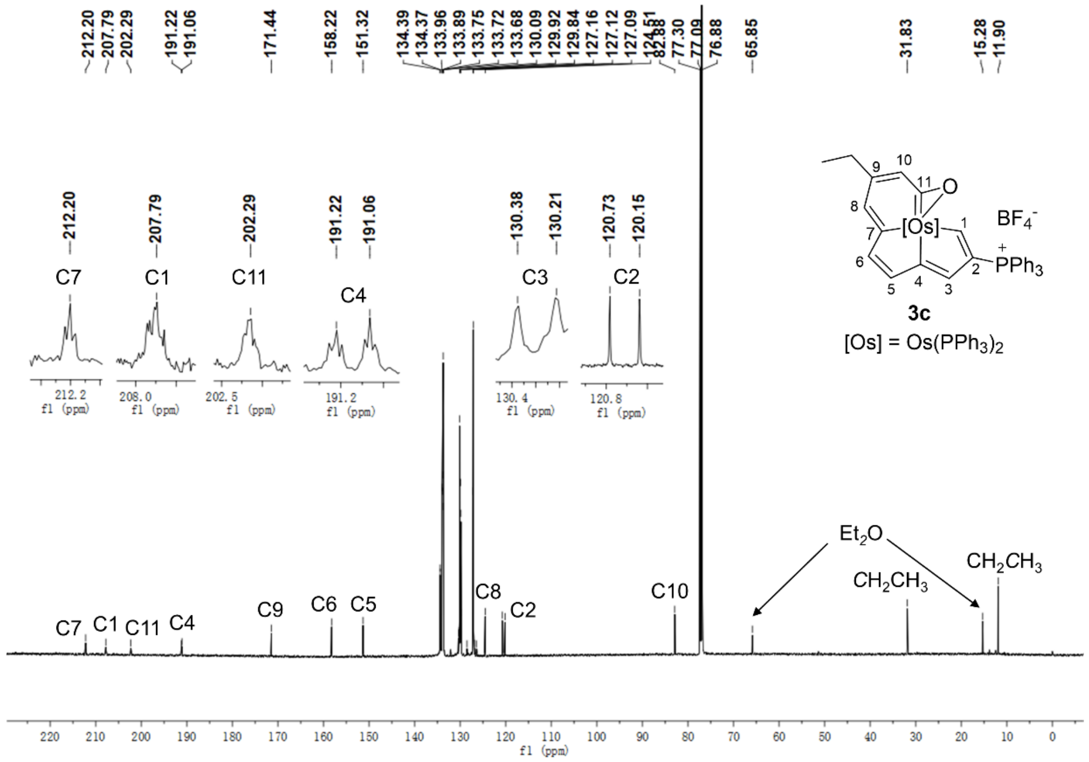

Supplementary Figure 12. The ^{13}C{^{1}H} NMR (151 MHz, $CDCl_3$) spectrum for **3c**.

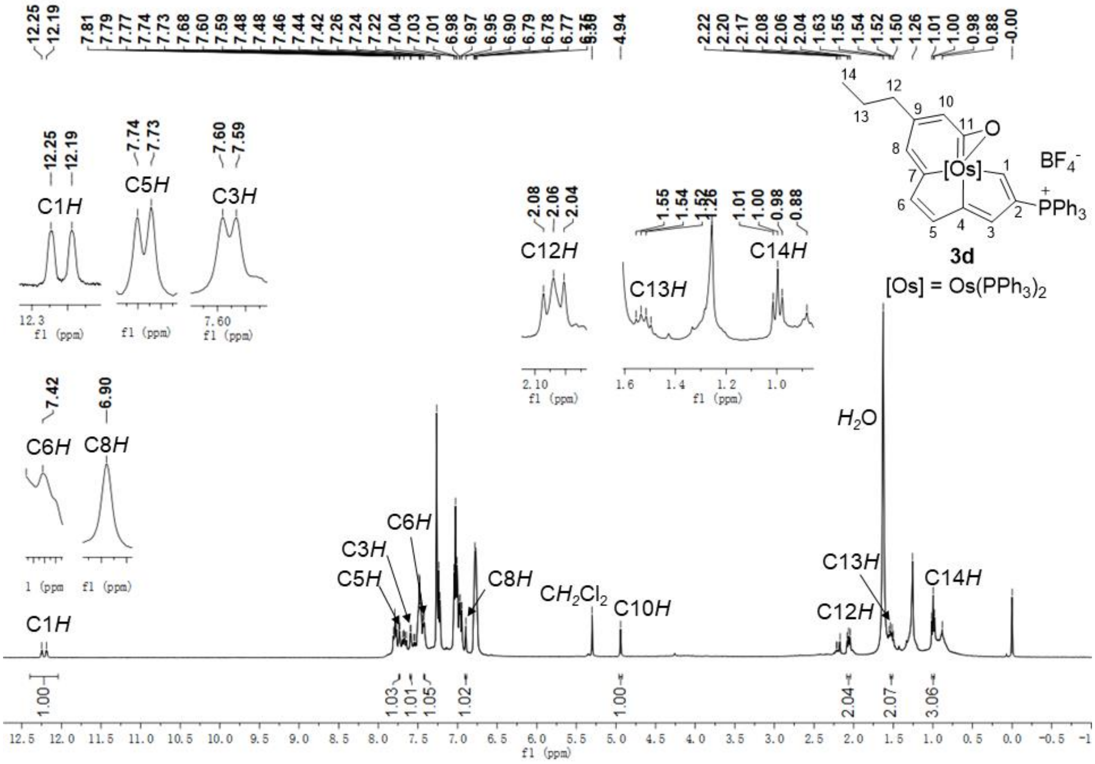

Supplementary Figure 13. The ^{1}H NMR (400 MHz, $CDCl_3$) spectrum for **3d**.

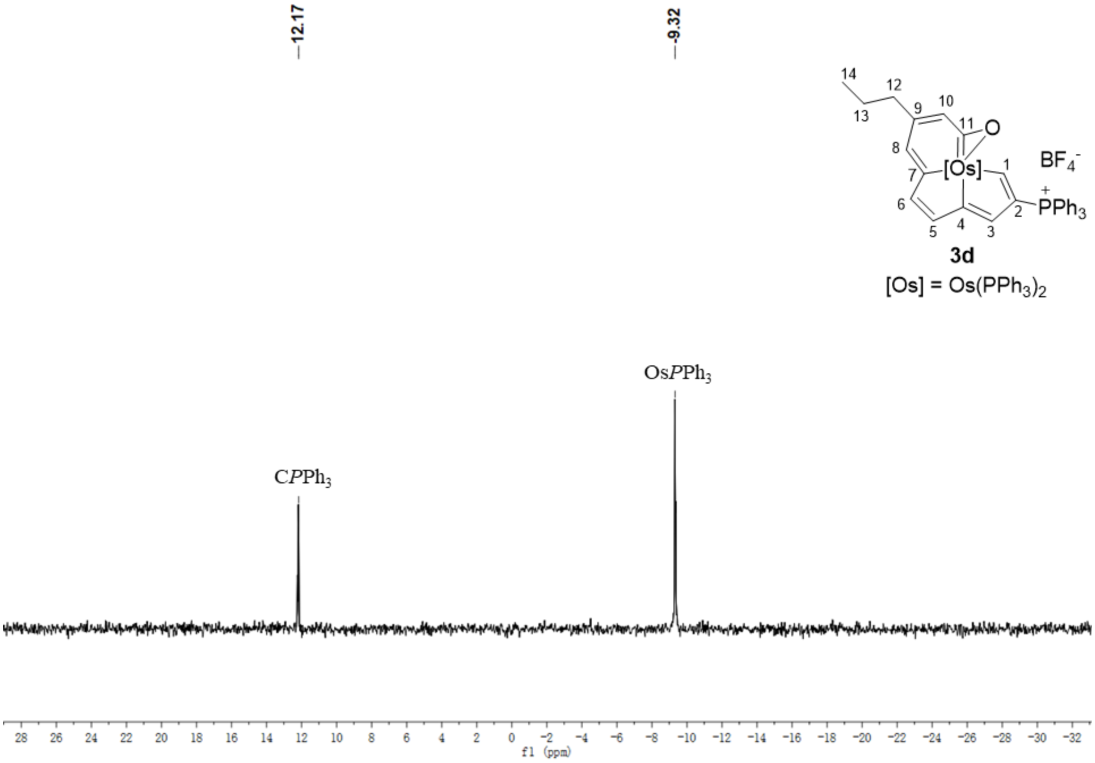

Supplementary Figure 14. The $^{31}P\{^{1}H\}$ NMR (162 MHz, $CDCl_3$) spectrum for **3d**.

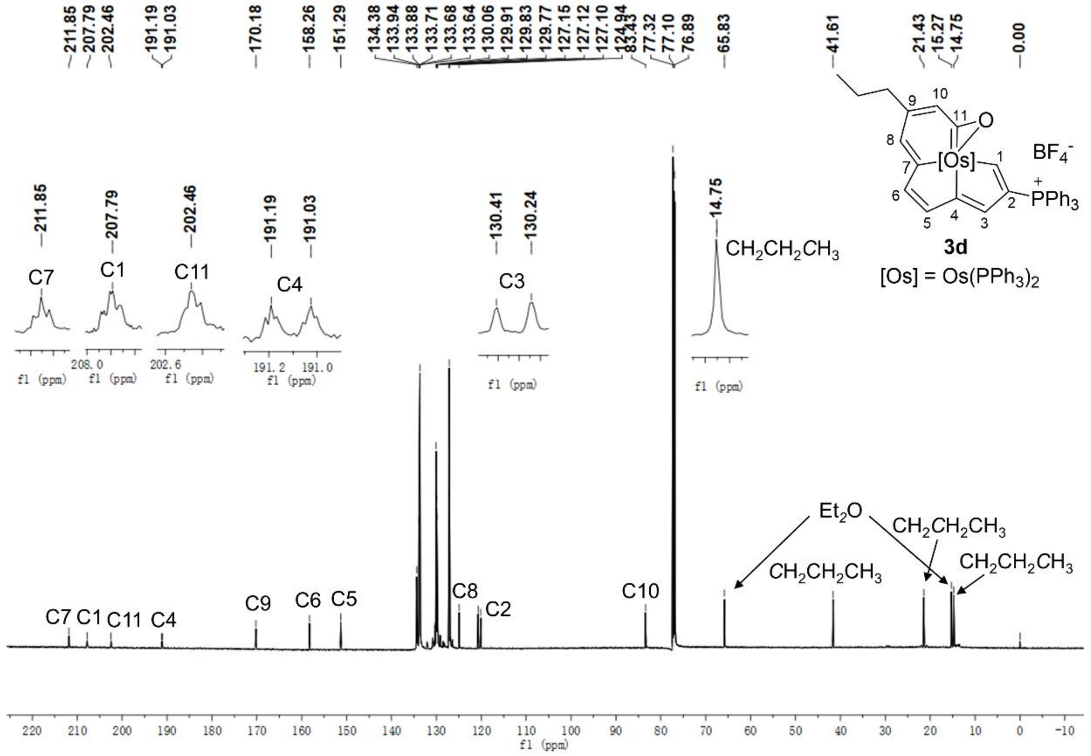

Supplementary Figure 15. The $^{13}C\{^{1}H\}$ NMR (151 MHz, $CDCl_3$) spectrum for **3d**.

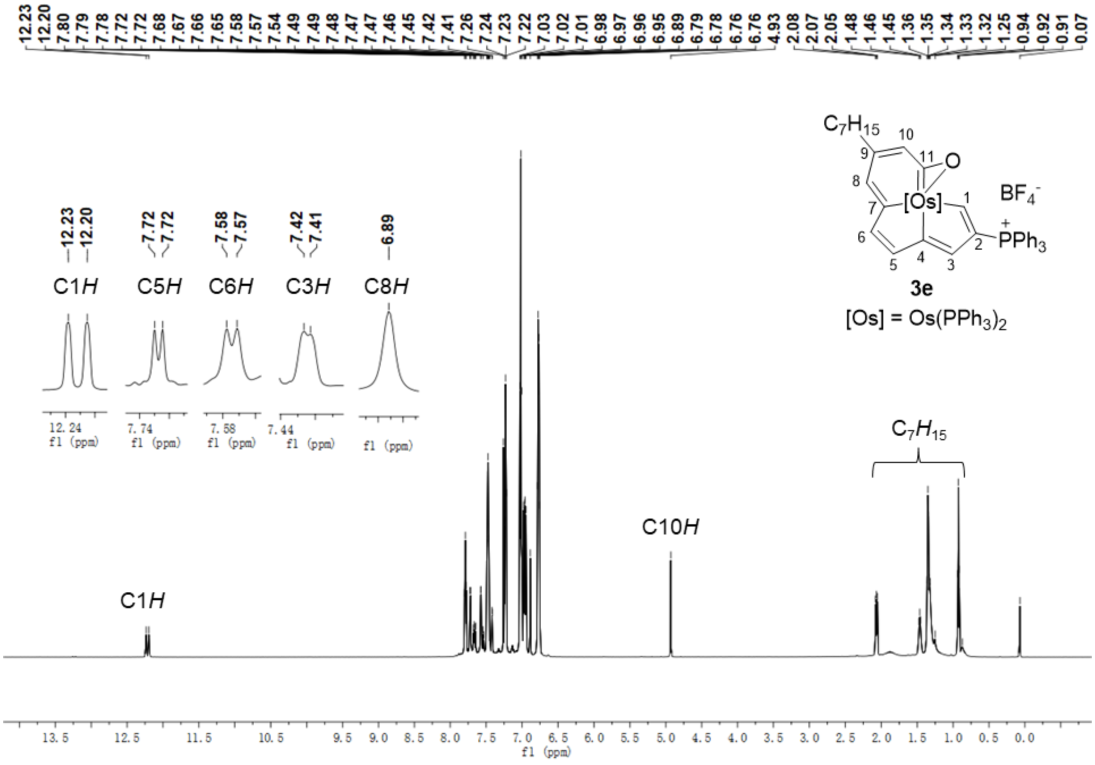

Supplementary Figure 16. The ^{1}H NMR (600 MHz, $CDCl_3$) spectrum for **3e**.

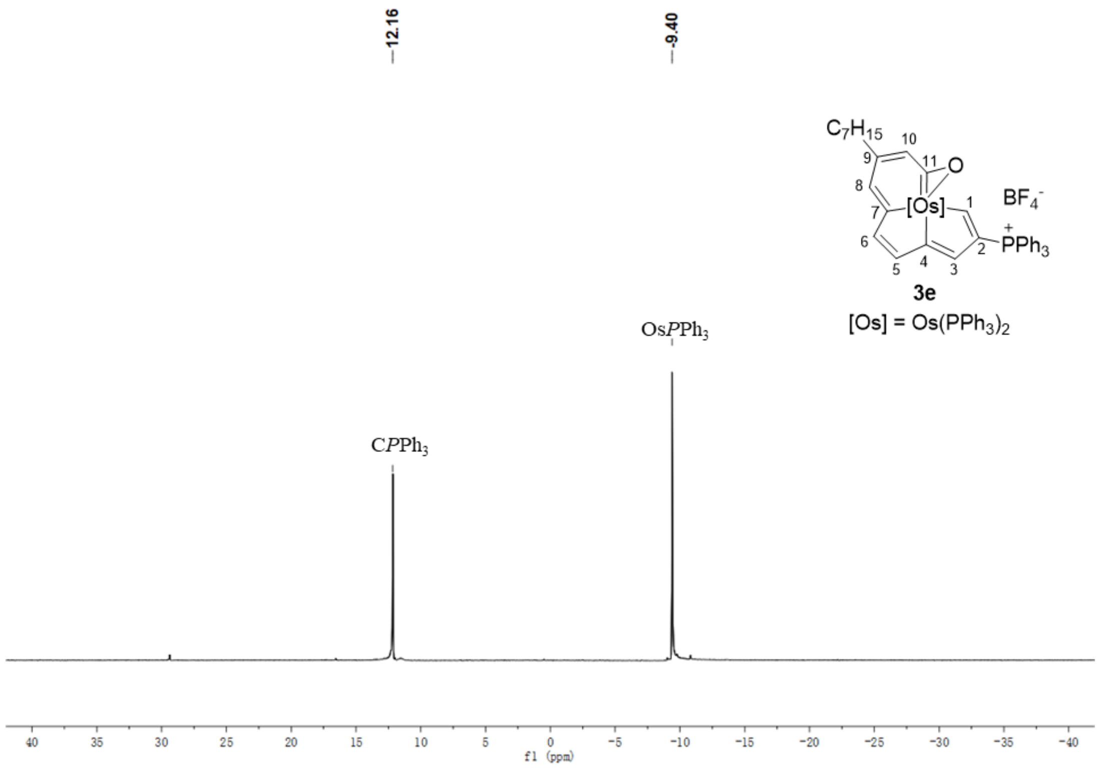

Supplementary Figure 17. The $^{31}P\{^1H\}$ NMR (243 MHz, $CDCl_3$) spectrum for **3e**.

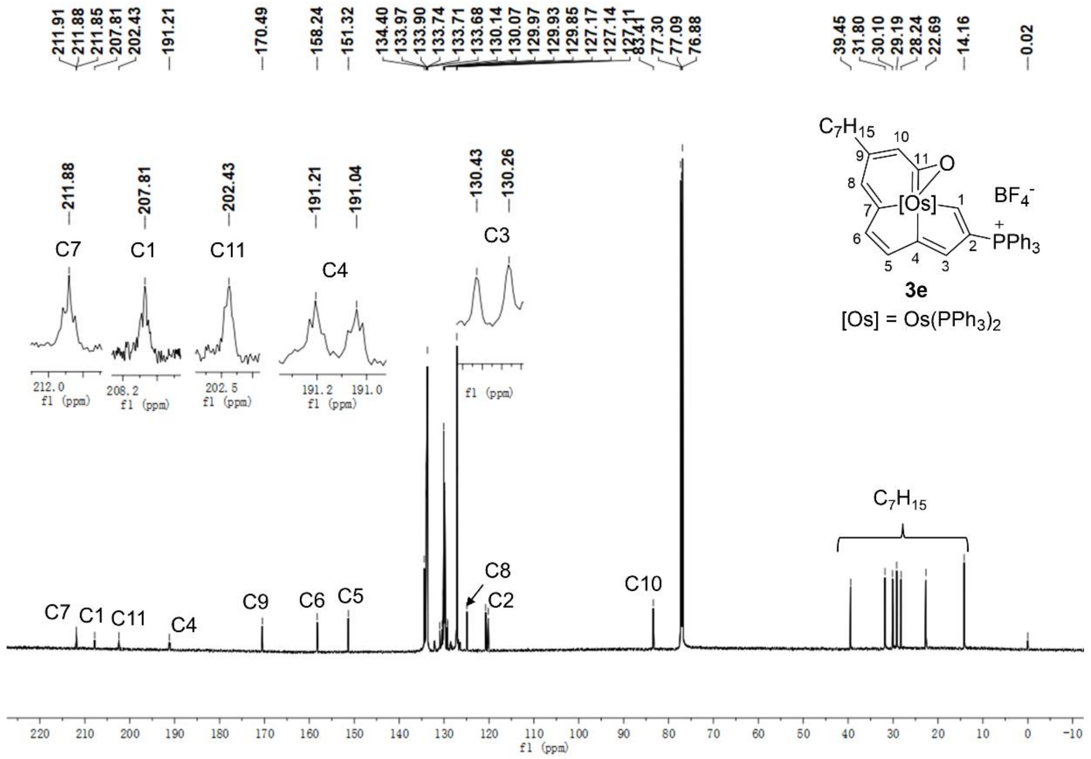

Supplementary Figure 18. The $^{13}C\{^1H\}$ NMR (151 MHz, $CDCl_3$) spectrum for **3e**.

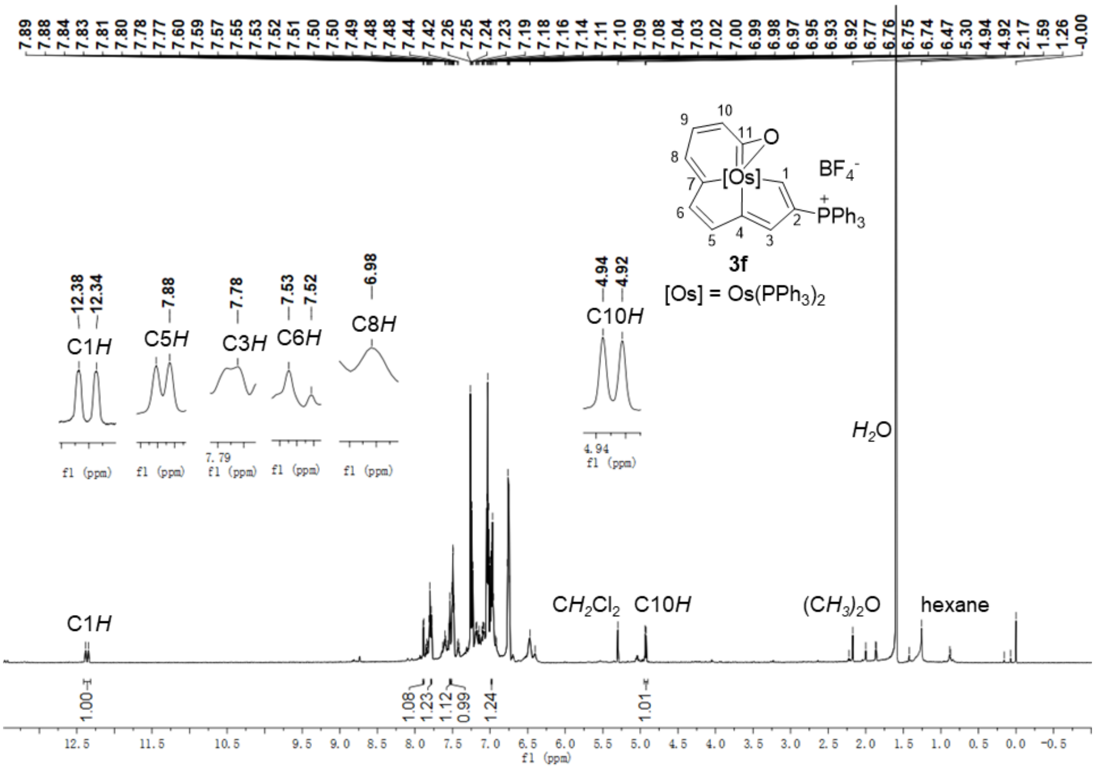

Supplementary Figure 19. The ^{1}H NMR (600 MHz, $CDCl_3$) spectrum for **3f**.

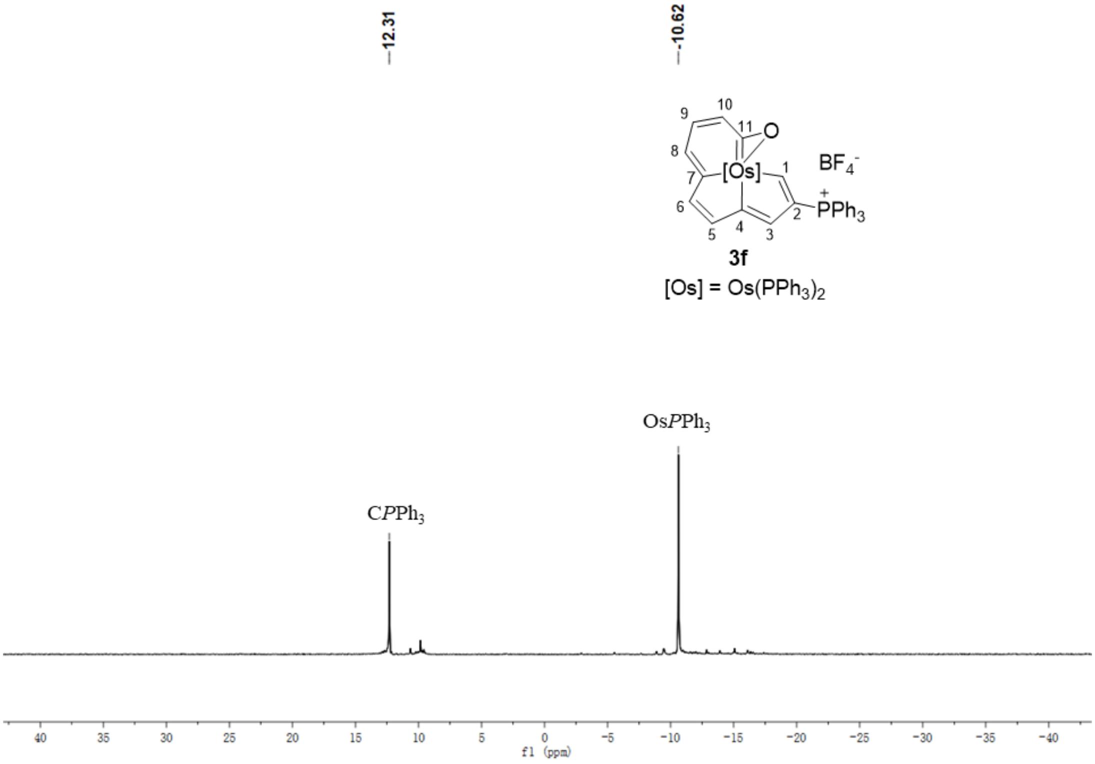

Supplementary Figure 20. The ^{31}P{^{1}H} NMR (243 MHz, $CDCl_3$) spectrum for **3f**.

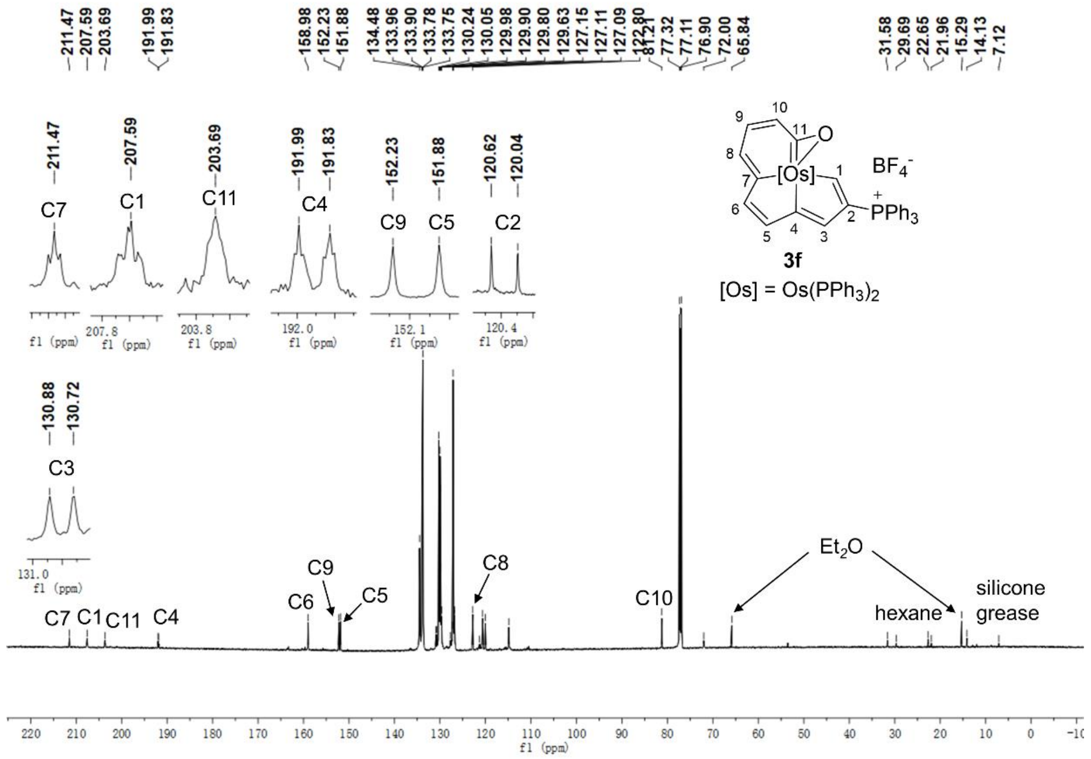

Supplementary Figure 21. The $^{13}C\{^{1}H\}$ NMR (151 MHz, $CDCl_3$) spectrum for **3f**.

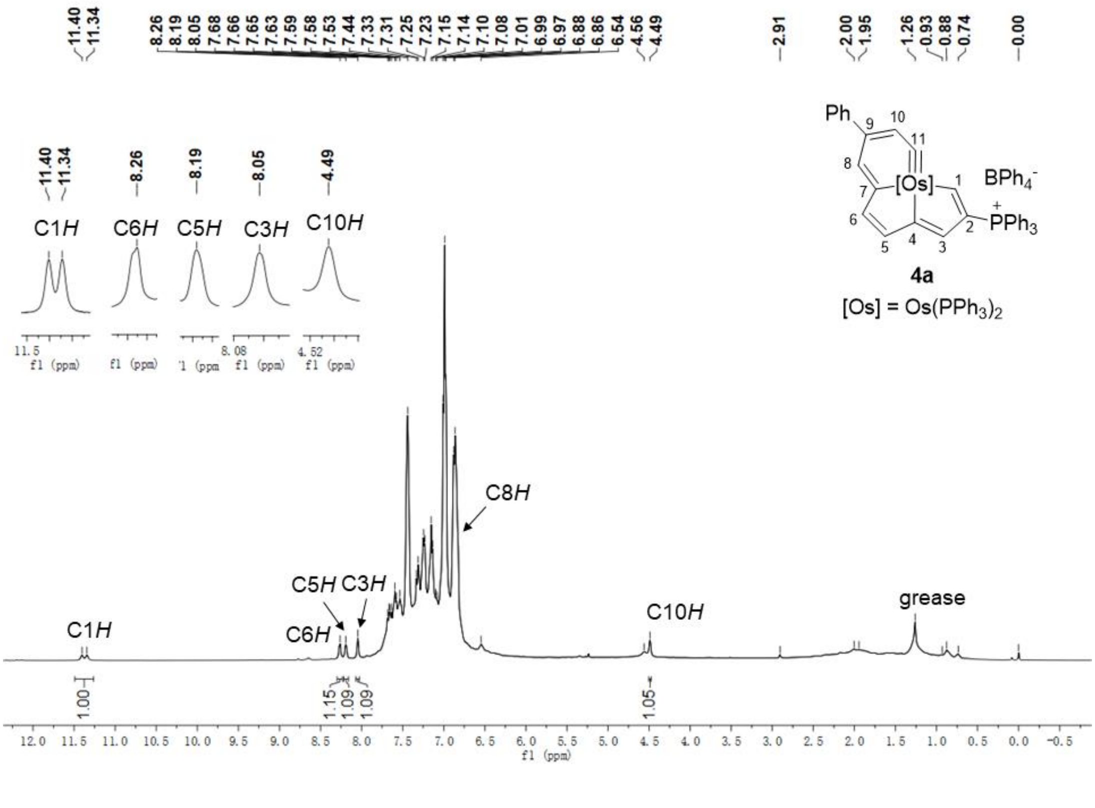

Supplementary Figure 22. The ^{1}H NMR (400 MHz, $CDCl_3$) spectrum for **4a**.

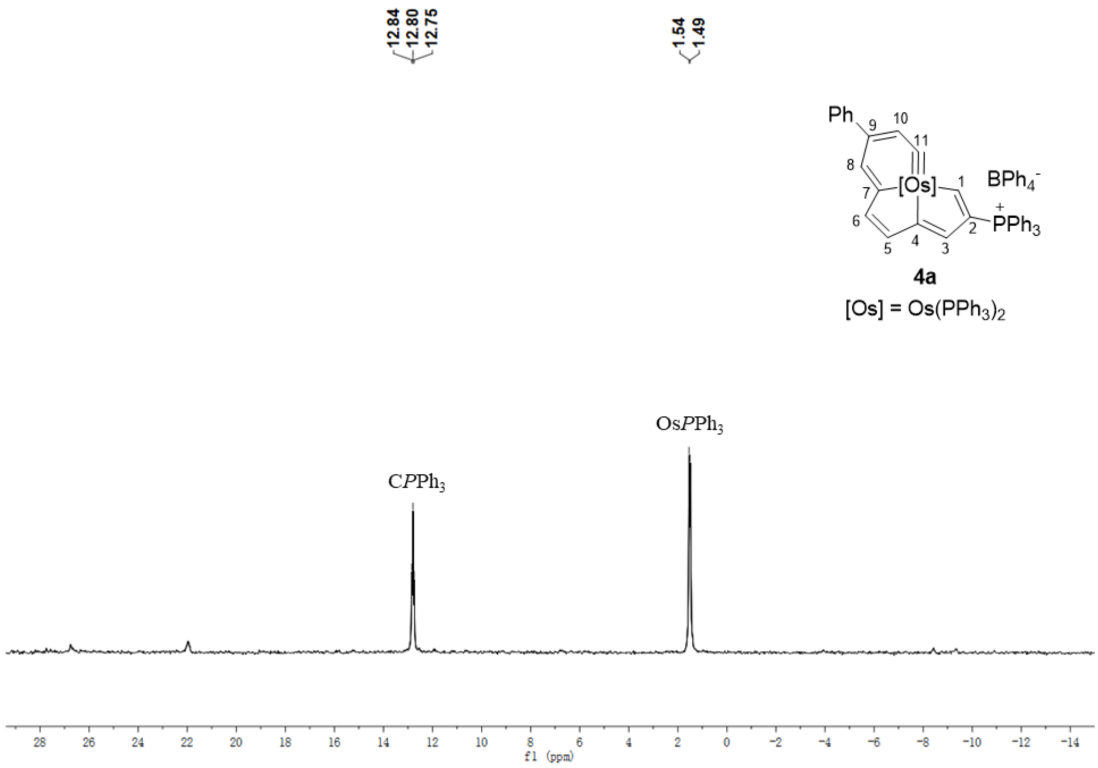

Supplementary Figure 23. The $^{31}P\{^1H\}$ NMR (162 MHz, $CDCl_3$) spectrum for **4a**.

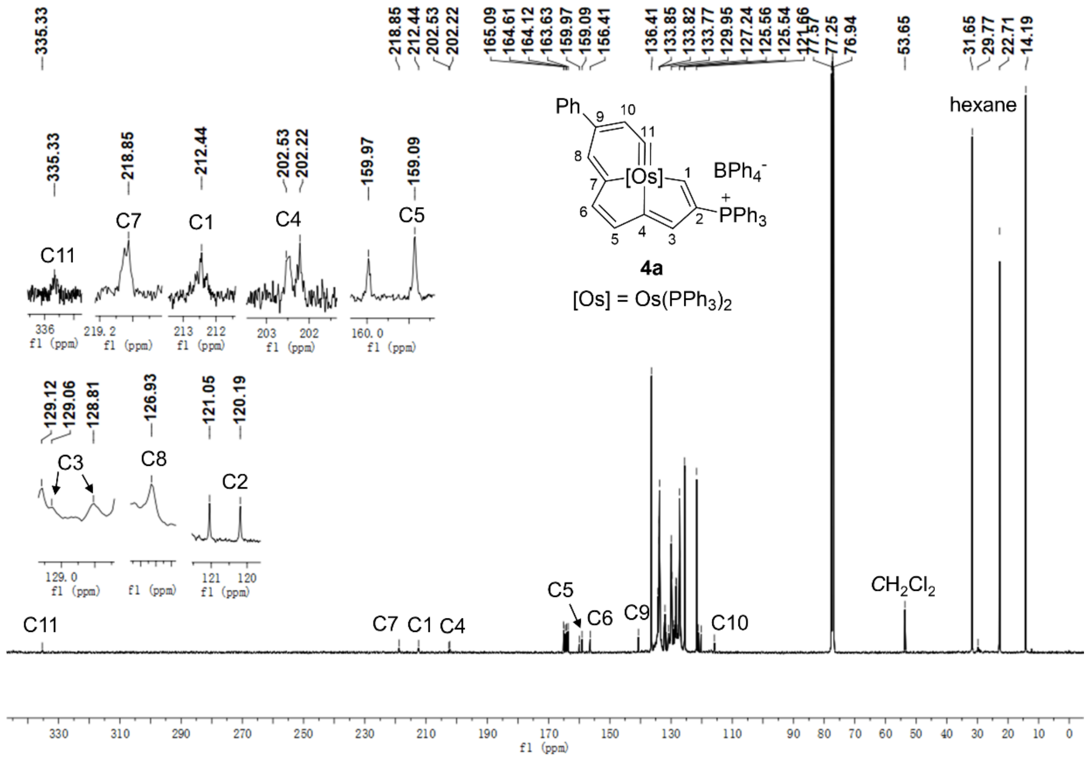

Supplementary Figure 24. The $^{13}C\{^1H\}$ NMR (101 MHz, $CDCl_3$) spectrum for **4a**.

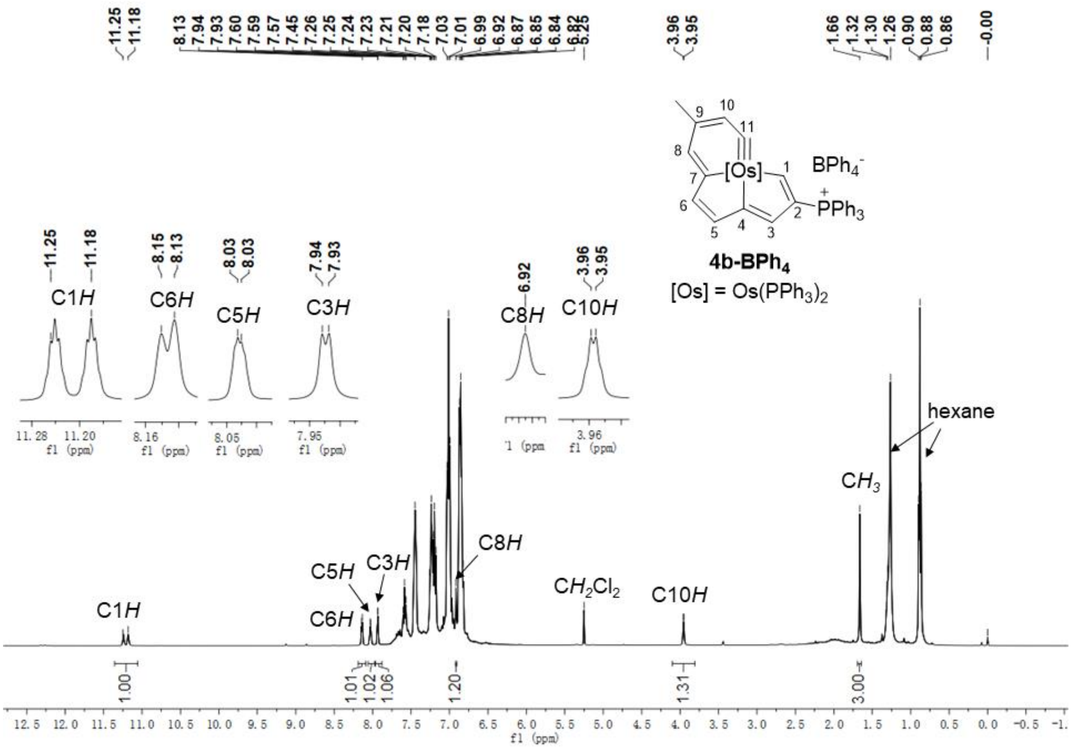

Supplementary Figure 25. The ^{1}H NMR (400 MHz, $CDCl_3$) spectrum for **4b-BPh4**.

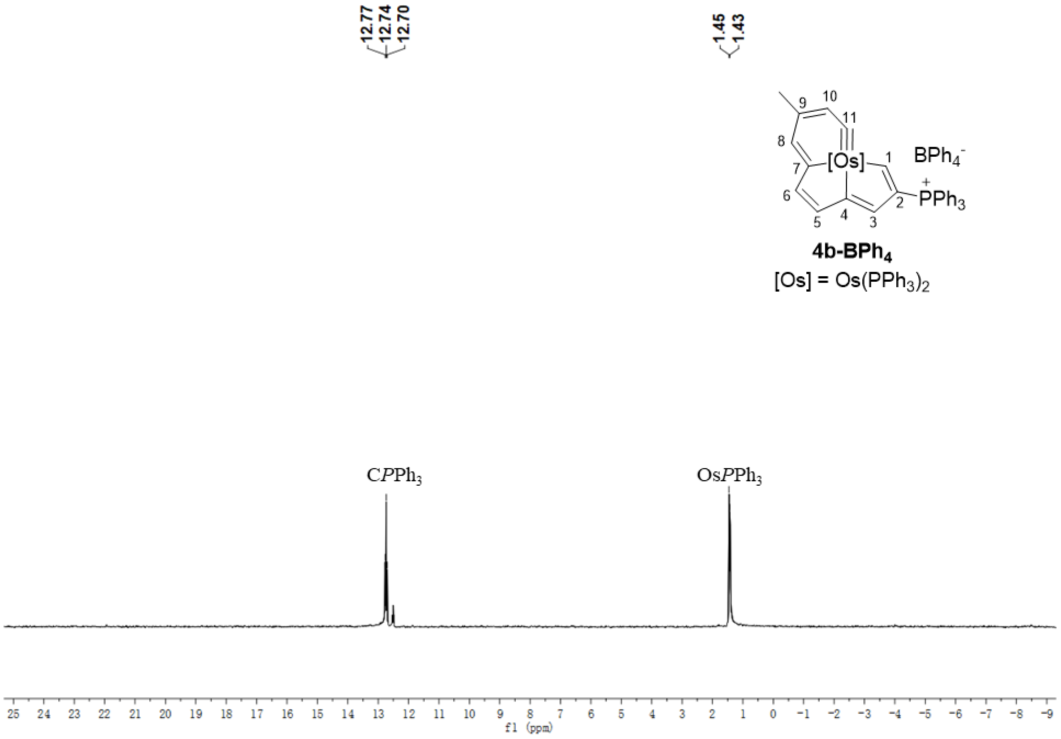

Supplementary Figure 26. The $^{31}P\{^1H\}$ NMR (243 MHz, $CDCl_3$) spectrum for **4b-BPh4**.

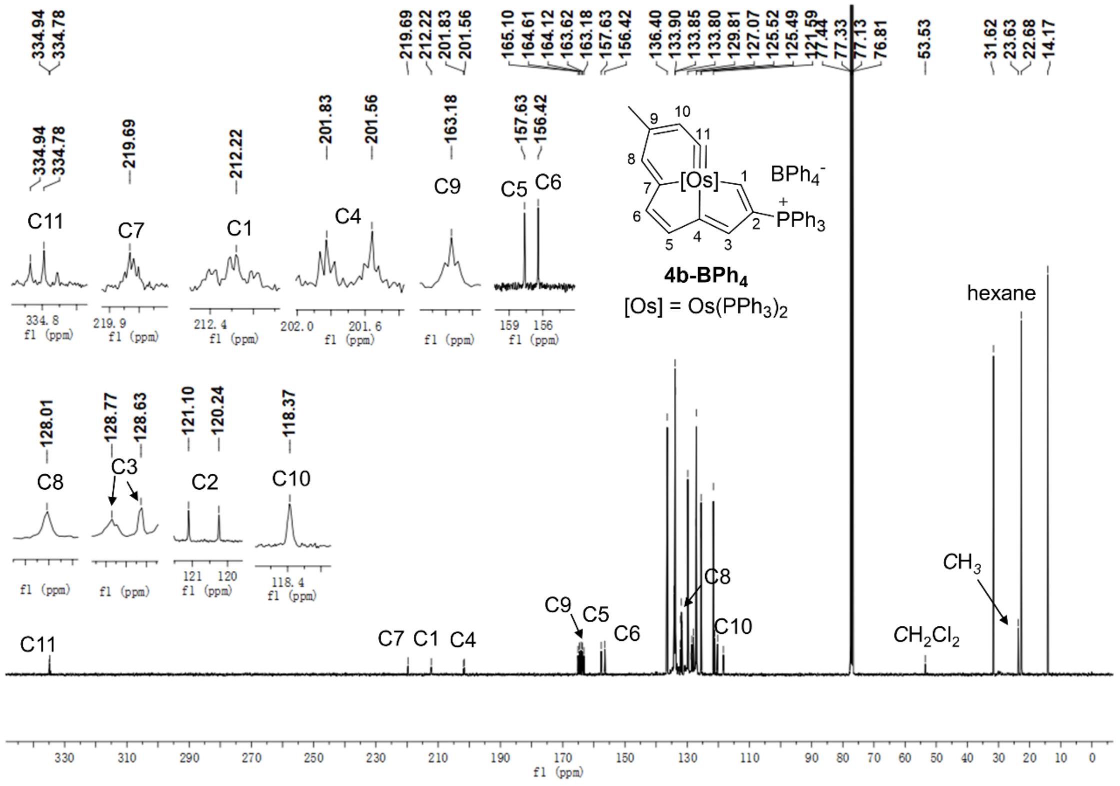

Supplementary Figure 27. The $^{13}C\{^1H\}$ NMR (101 MHz, $CDCl_3$) spectrum for **4b-BPh₄**.

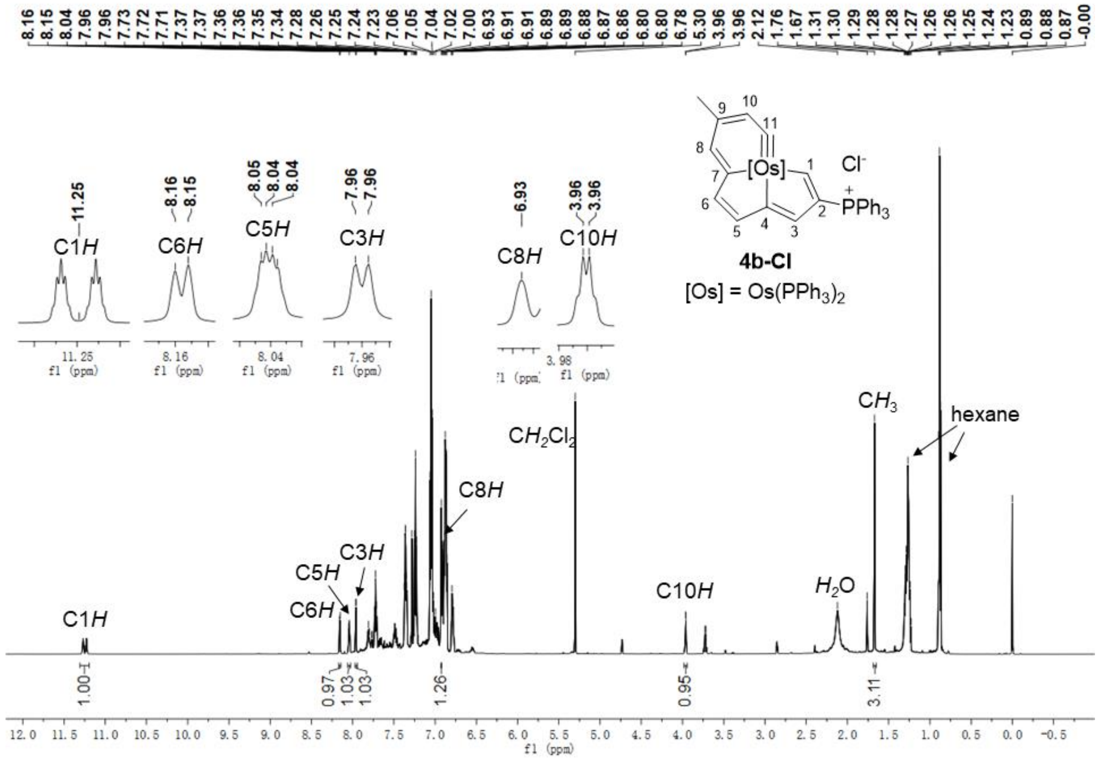

Supplementary Figure 28. The ^1H NMR (600 MHz, $CDCl_3$) spectrum for **4b-Cl**.

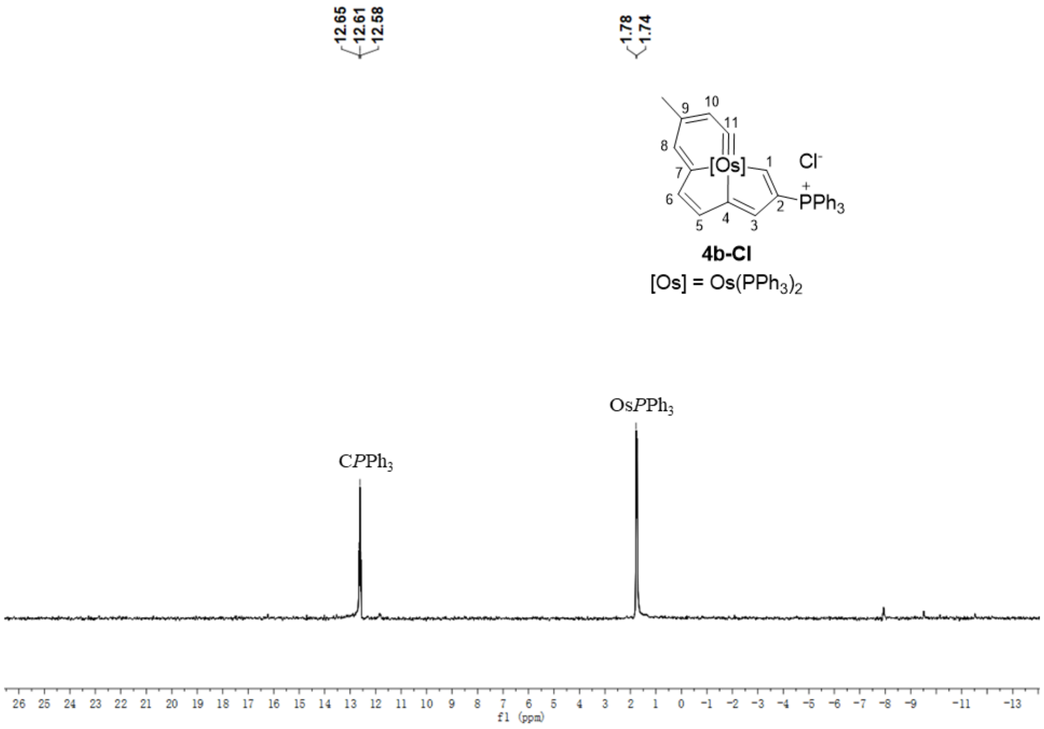

Supplementary Figure 29. The $^{31}P\{^{1}H\}$ NMR (243 MHz, $CDCl_3$) spectrum for **4b-Cl**.

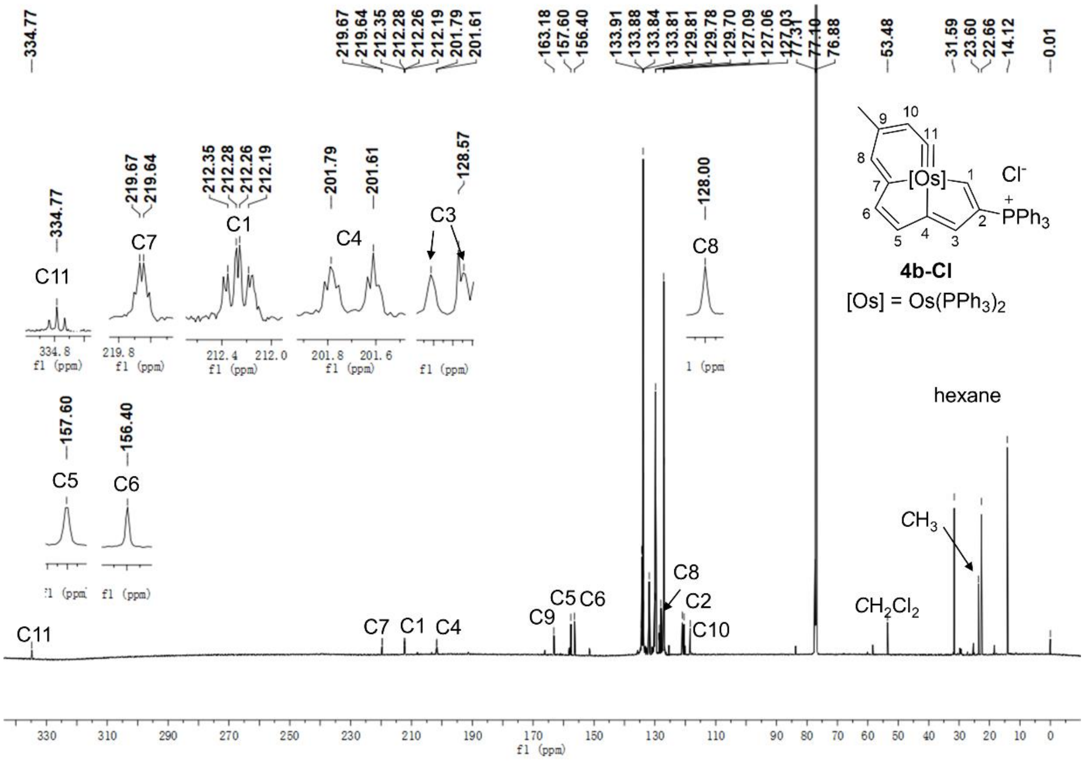

Supplementary Figure 30. The $^{13}C\{^{1}H\}$ NMR (151 MHz, $CDCl_3$) spectrum for **4b-Cl**.

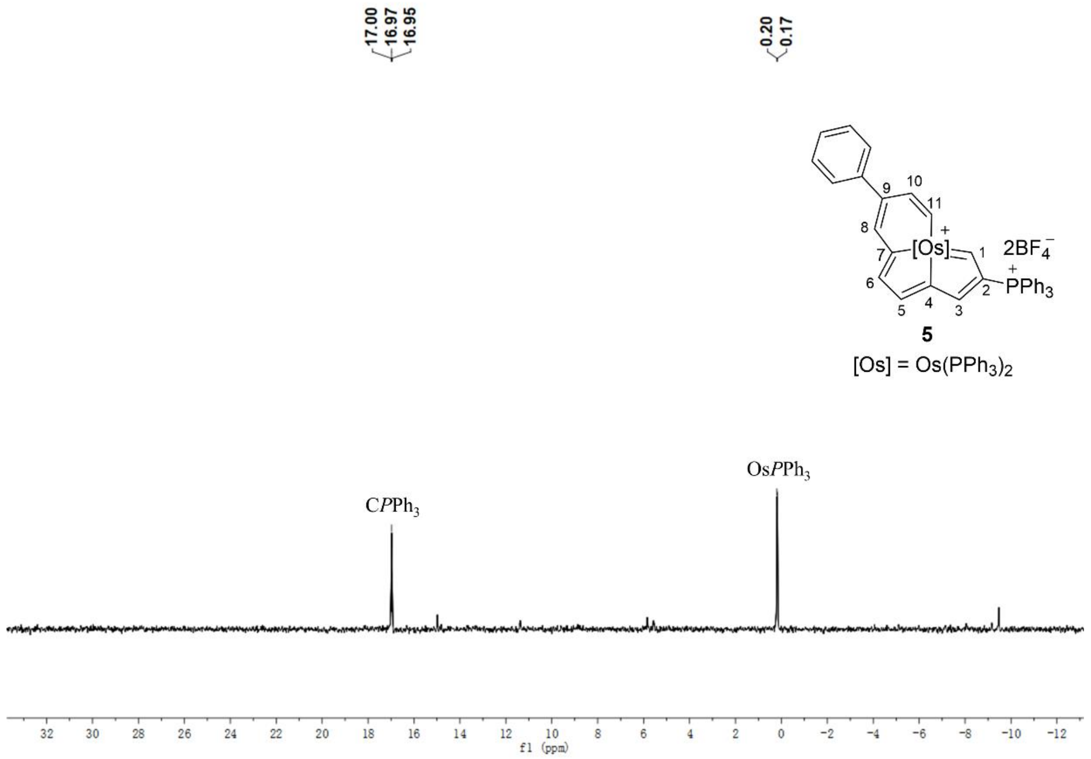

Supplementary Figure 31. The $^{31}P\{^{1}H\}$ NMR (243 MHz, CD_2Cl_2) spectrum for **5**.

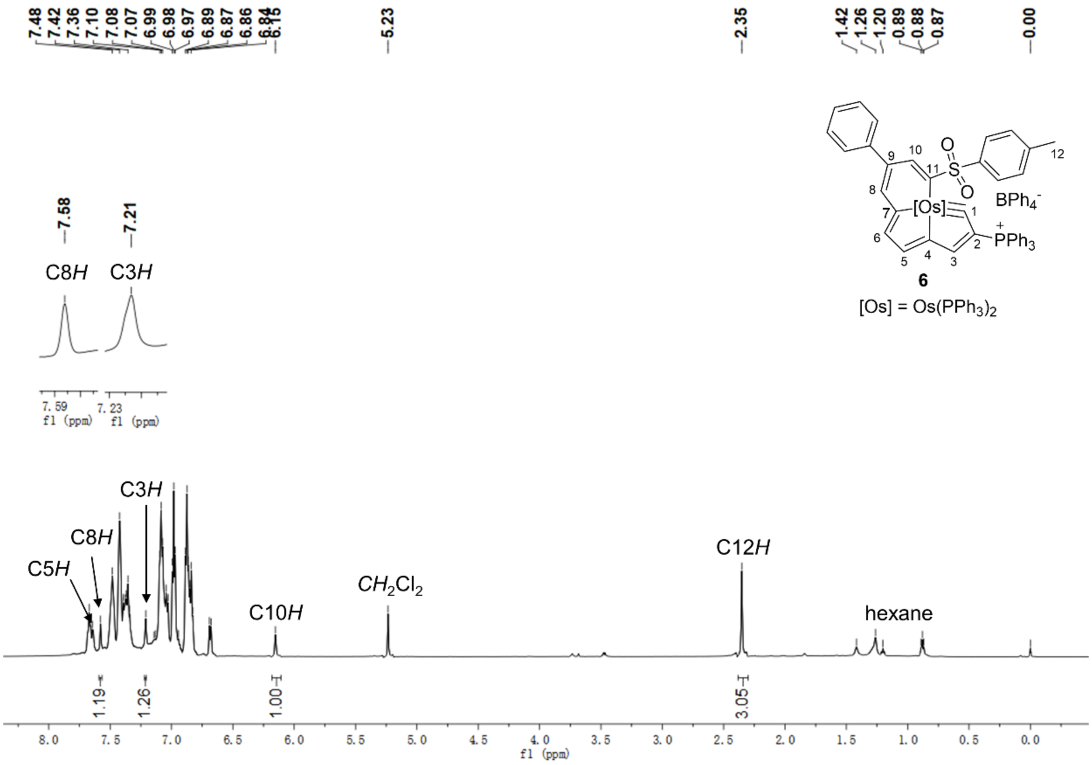

Supplementary Figure 32. The ^{1}H NMR (600 MHz, $CDCl_3$) spectrum for **6**.

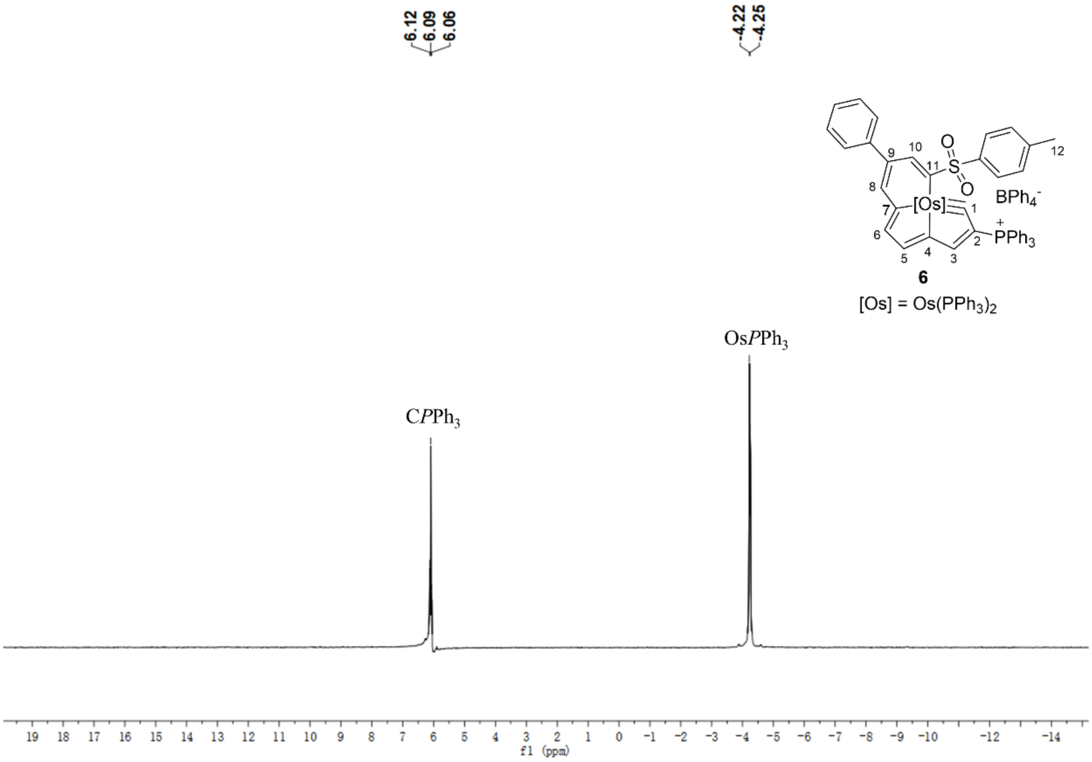

Supplementary Figure 33. The $^{31}P\{^1H\}$ NMR (243 MHz, $CDCl_3$) spectrum for **6**.

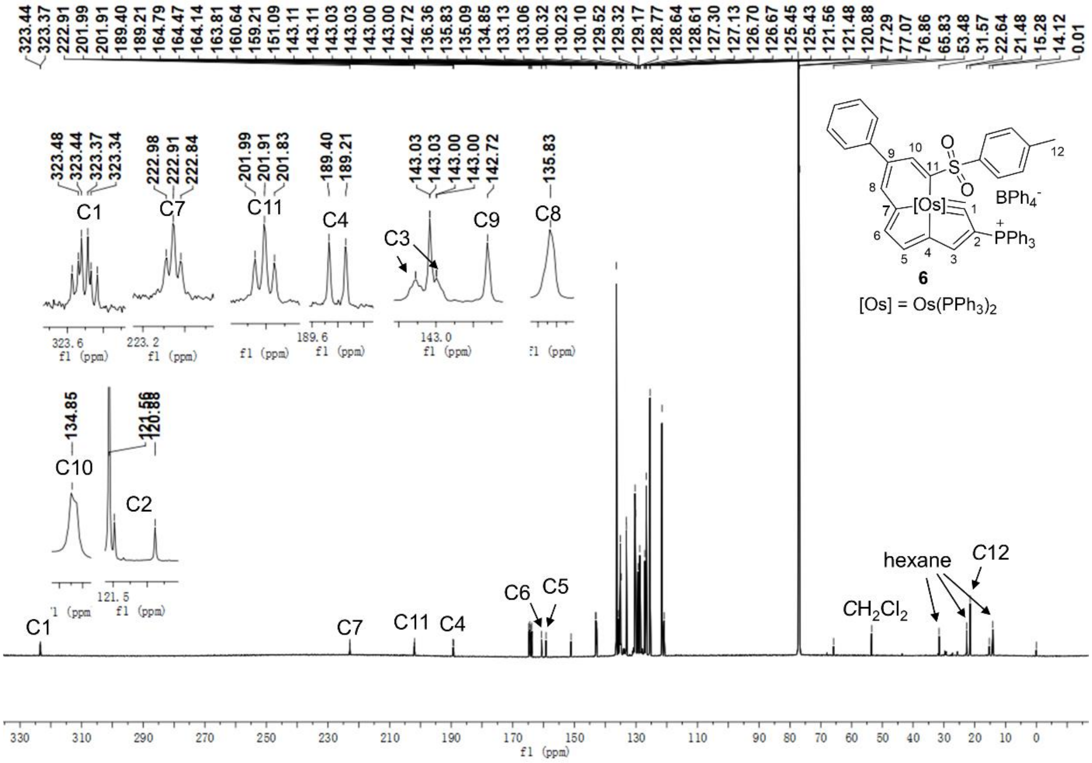

Supplementary Figure 34. The $^{13}C\{^1H\}$ NMR (151 MHz, $CDCl_3$) spectrum for **6**.

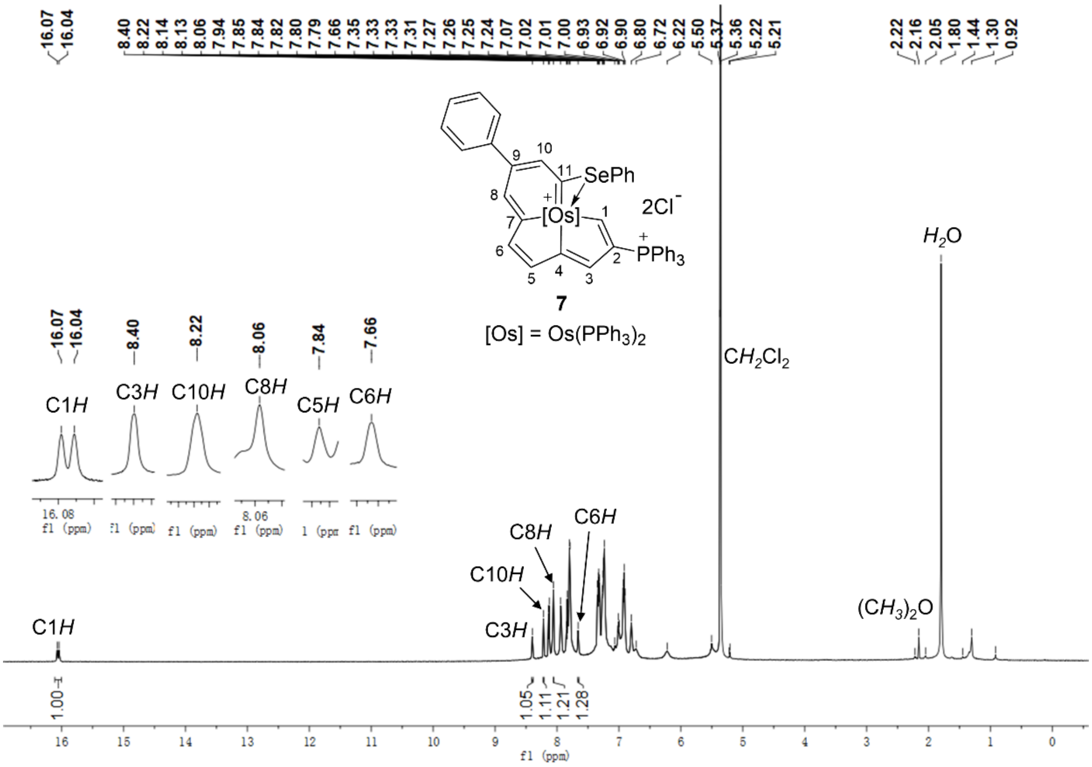

Supplementary Figure 35. The ^{1}H NMR (600 MHz, CD_2Cl_2) spectrum for **7**.

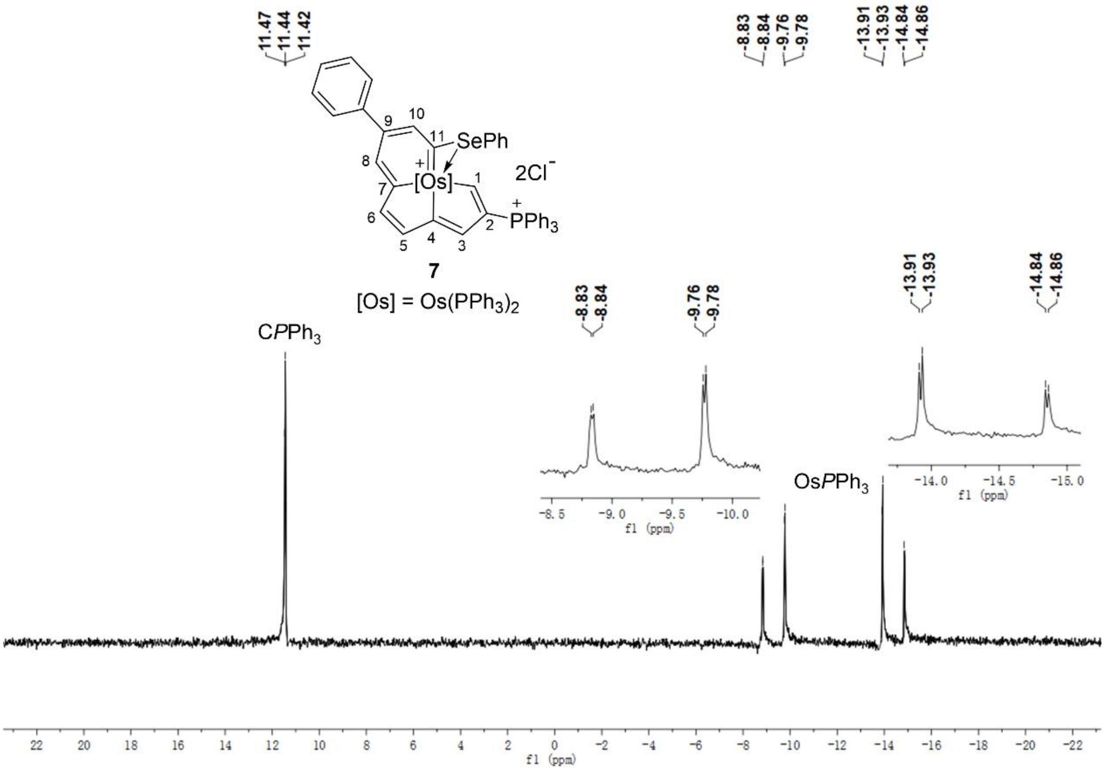

Supplementary Figure 36. The $^{31}P\{^{1}H\}$ NMR (243 MHz, CD_2Cl_2) spectrum for **7**.

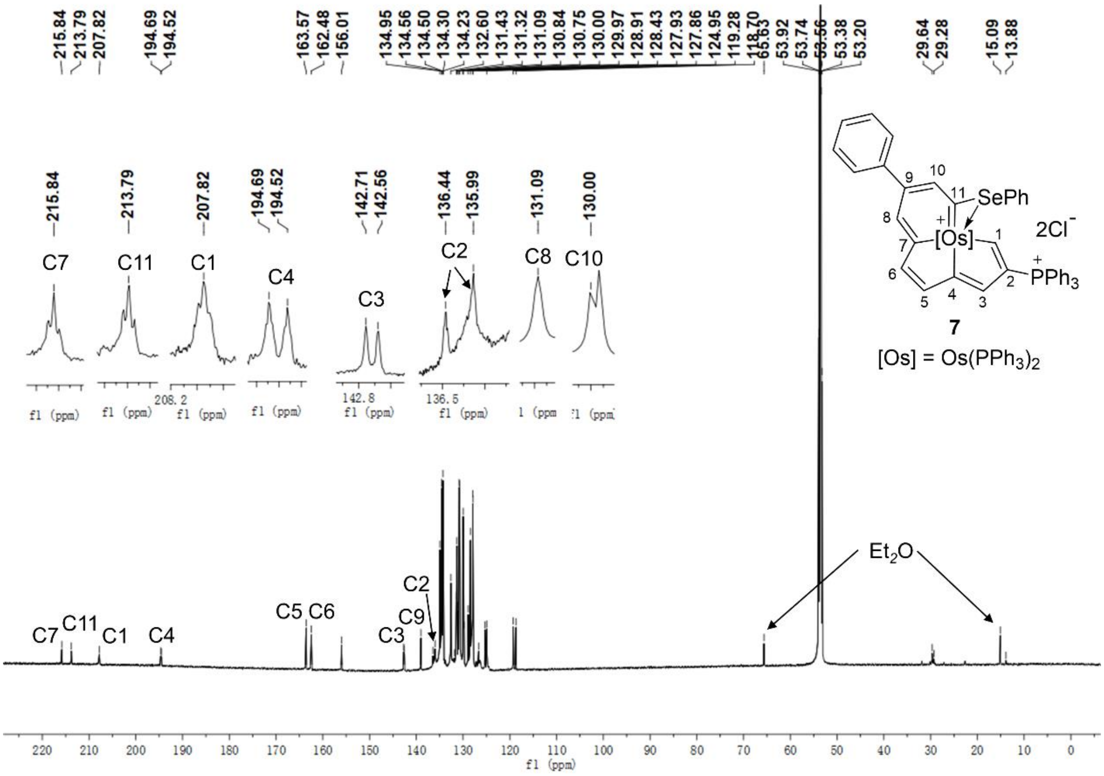

Supplementary Figure 37. The $^{13}C\{^{1}H\}$ NMR (151 MHz, CD_2Cl_2) spectrum for **7**.

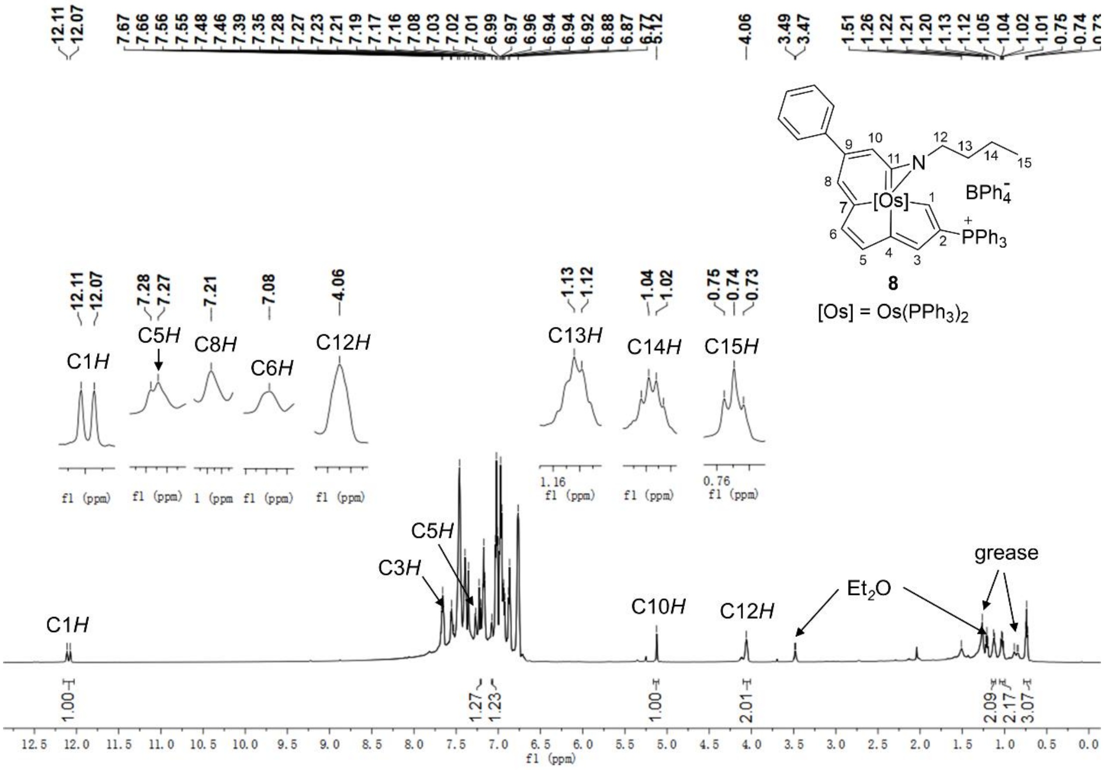

Supplementary Figure 38. The ^{1}H NMR (600 MHz, $CDCl_3$) spectrum for **8**.

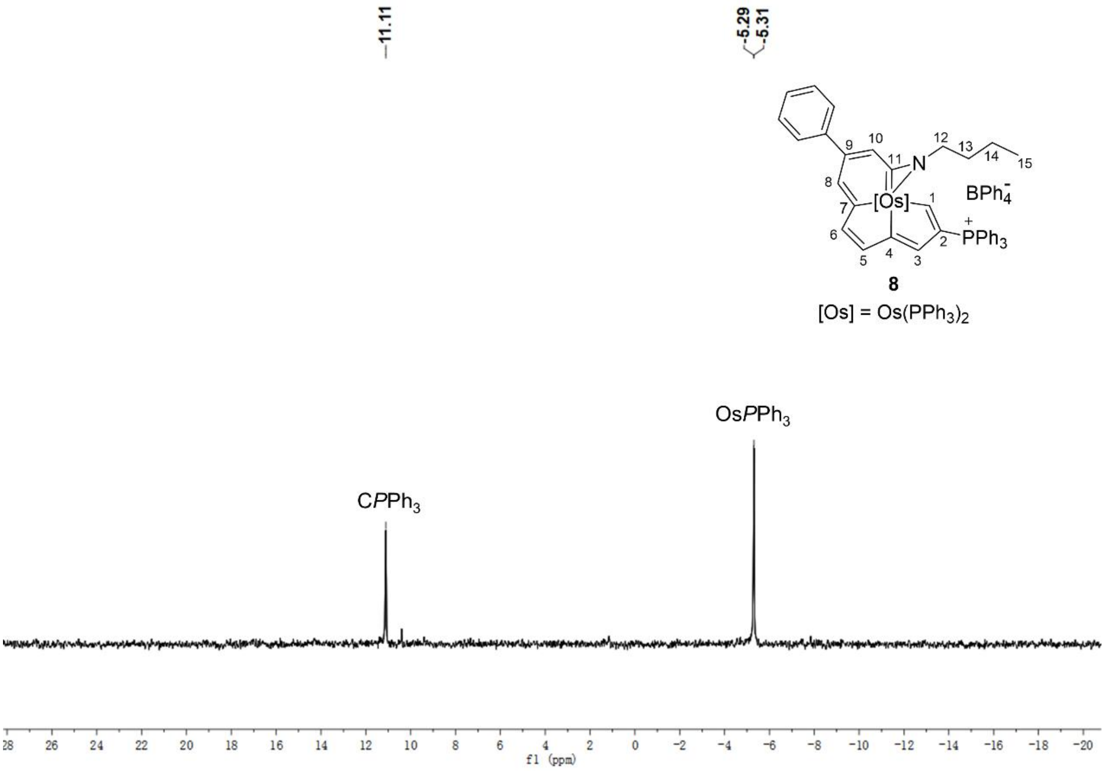

Supplementary Figure 39. The $^{31}P\{^{1}H\}$ NMR (243 MHz, $CDCl_3$) spectrum for **8**.

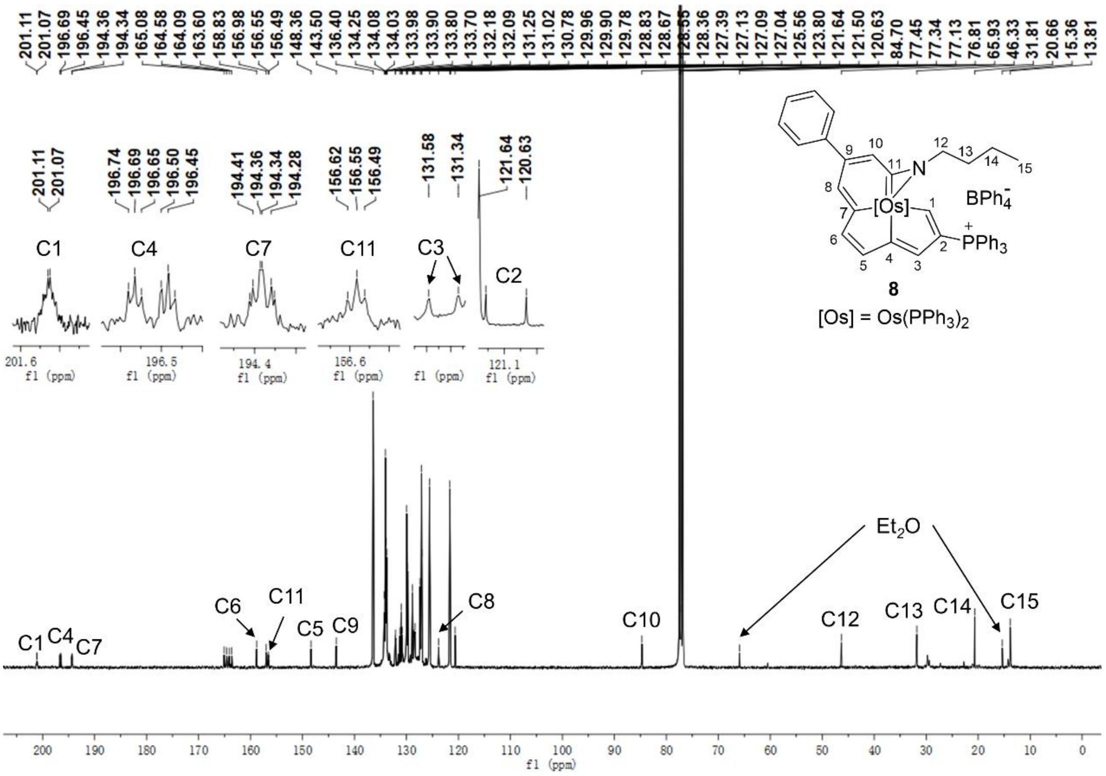

Supplementary Figure 40. The $^{13}C\{^{1}H\}$ NMR (101 MHz, $CDCl_3$) spectrum for **8**.

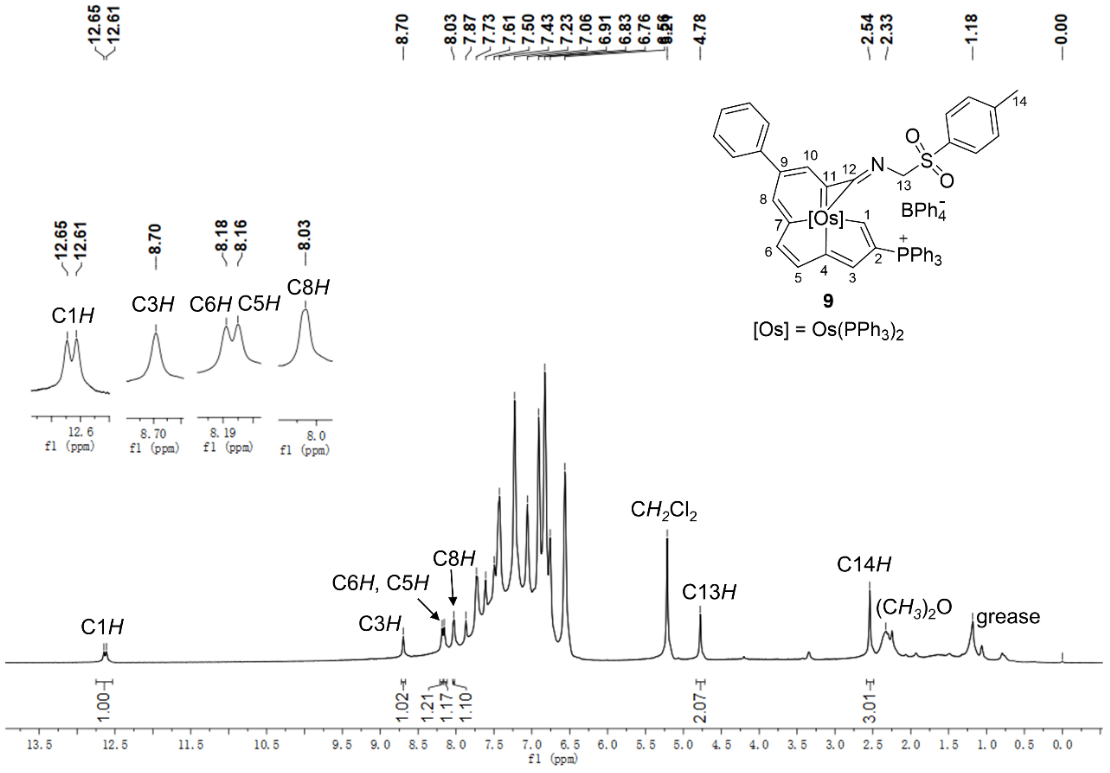

Supplementary Figure 41. The ^{1}H NMR (600 MHz, CD_2Cl_2) spectrum for **9**.

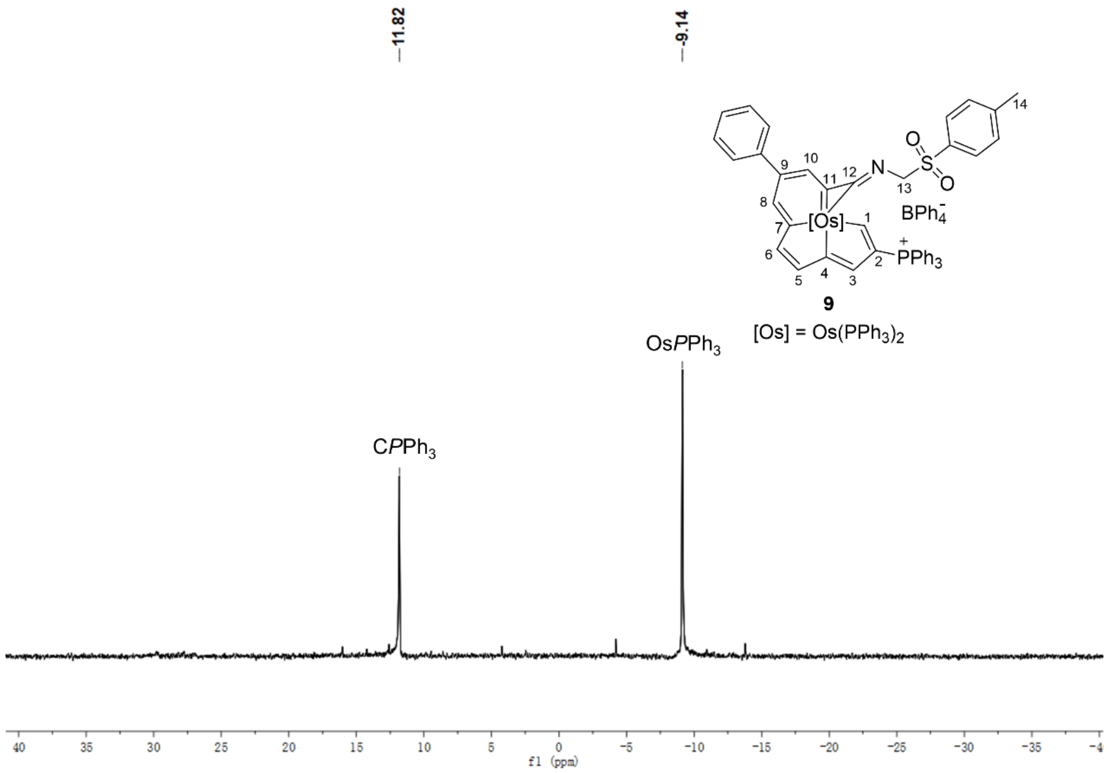

Supplementary Figure 42. The ^{31}P{^{1}H} NMR (243 MHz, CD_2Cl_2) spectrum for **9**.

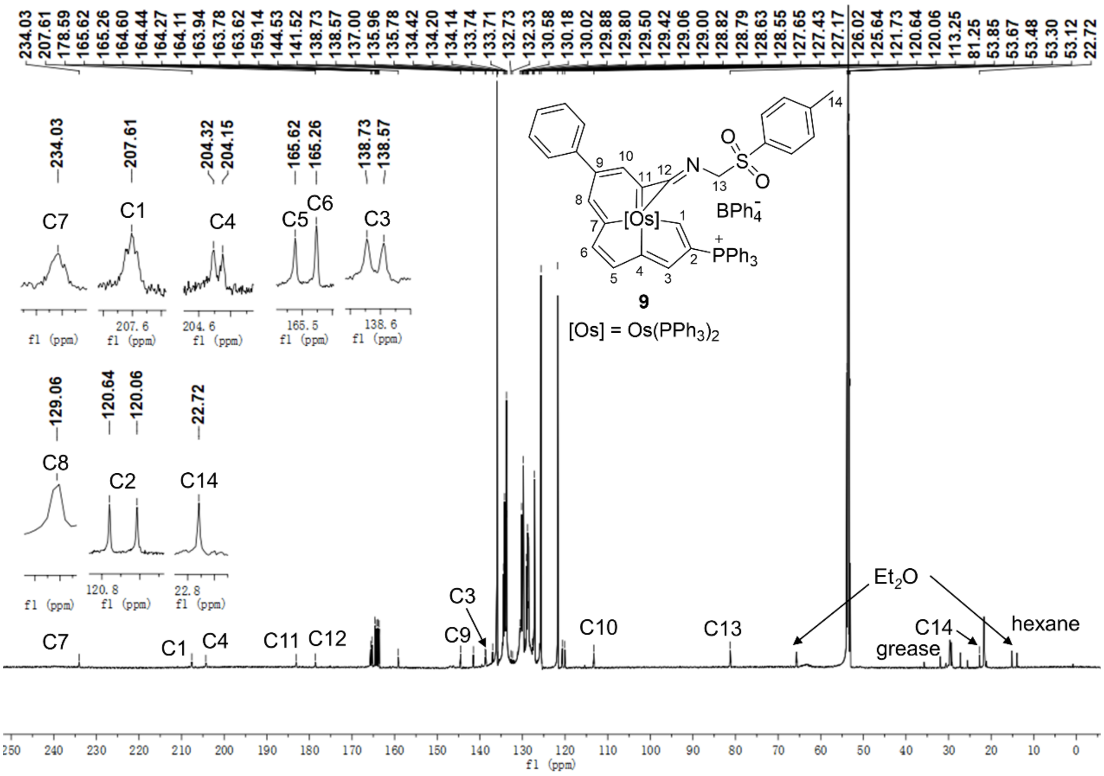

Supplementary Figure 43. The $^{13}C\{^1H\}$ NMR (151 MHz, CD_2Cl_2) spectrum for **9**.

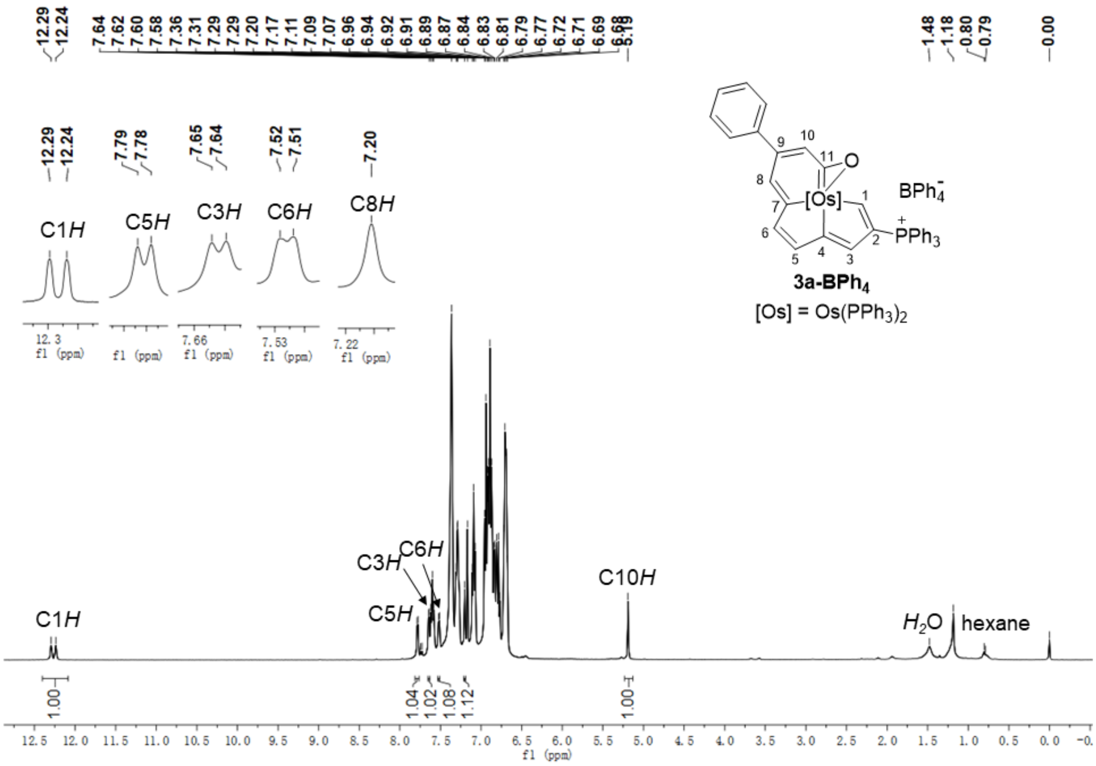

Supplementary Figure 44. The ^1H NMR (600 MHz, $CDCl_3$) spectrum for **3a-BPh4**.

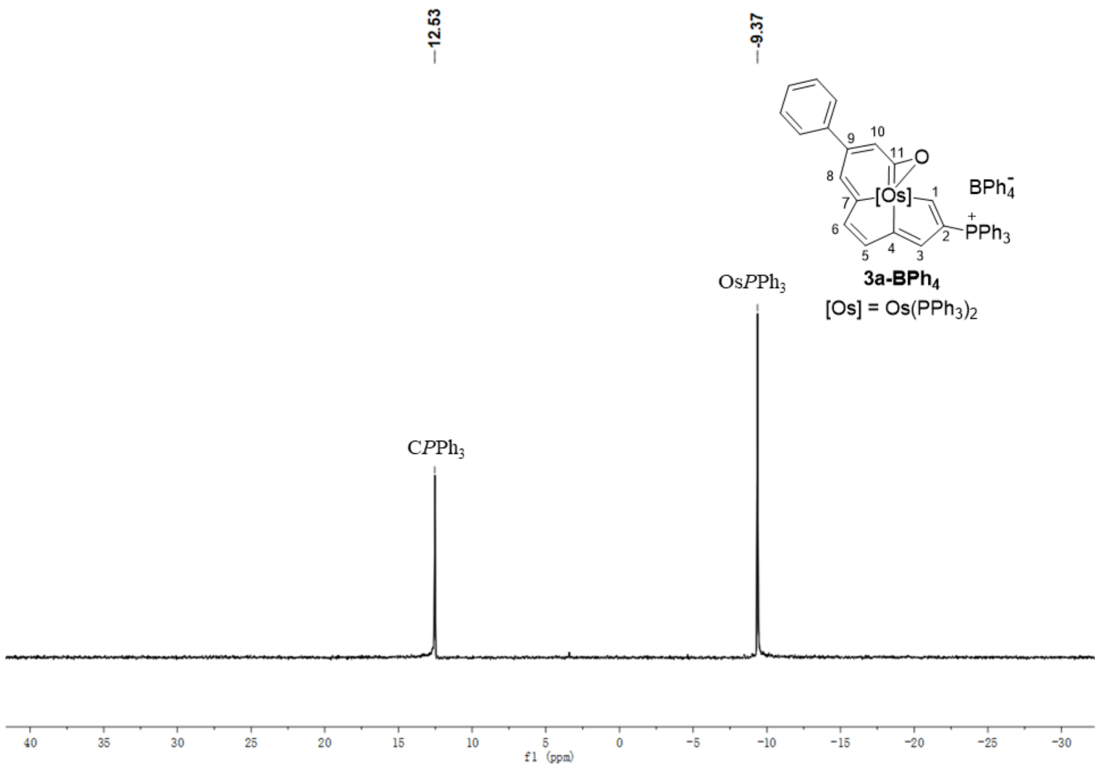

Supplementary Figure 45. The $^{31}P\{^{1}H\}$ NMR (243 MHz, $CDCl_3$) spectrum for **3a-BPh$_4$**.

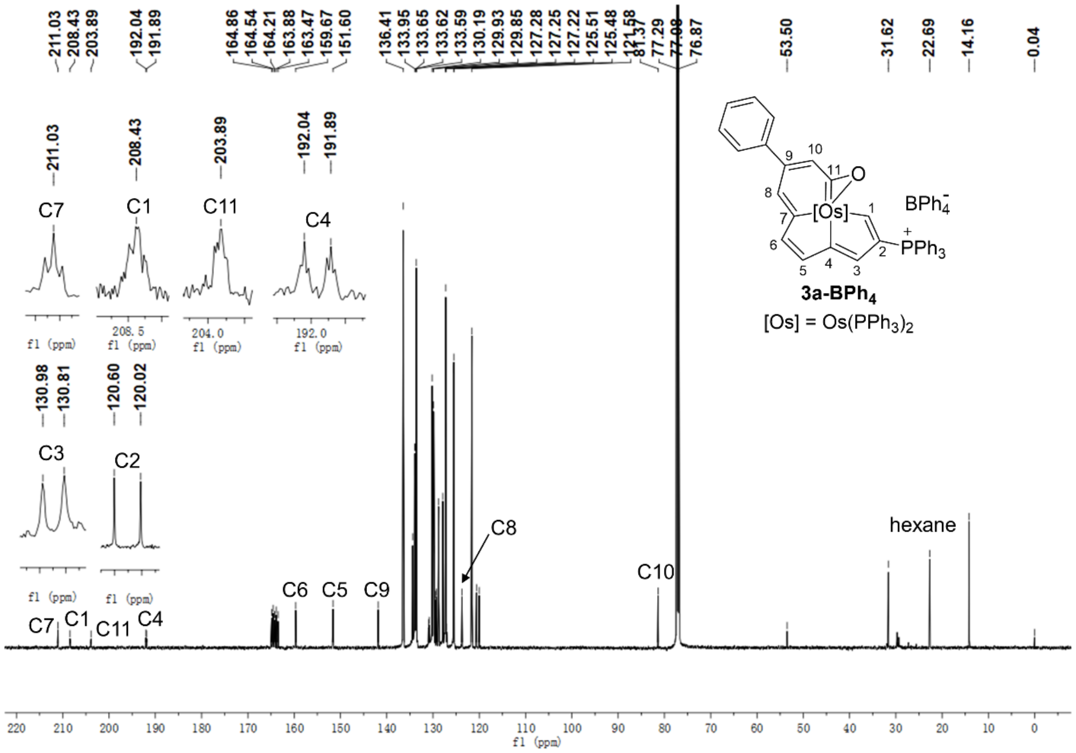

Supplementary Figure 46. The $^{13}C\{^{1}H\}$ NMR (151 MHz, $CDCl_3$) spectrum for

3a-BPh4.

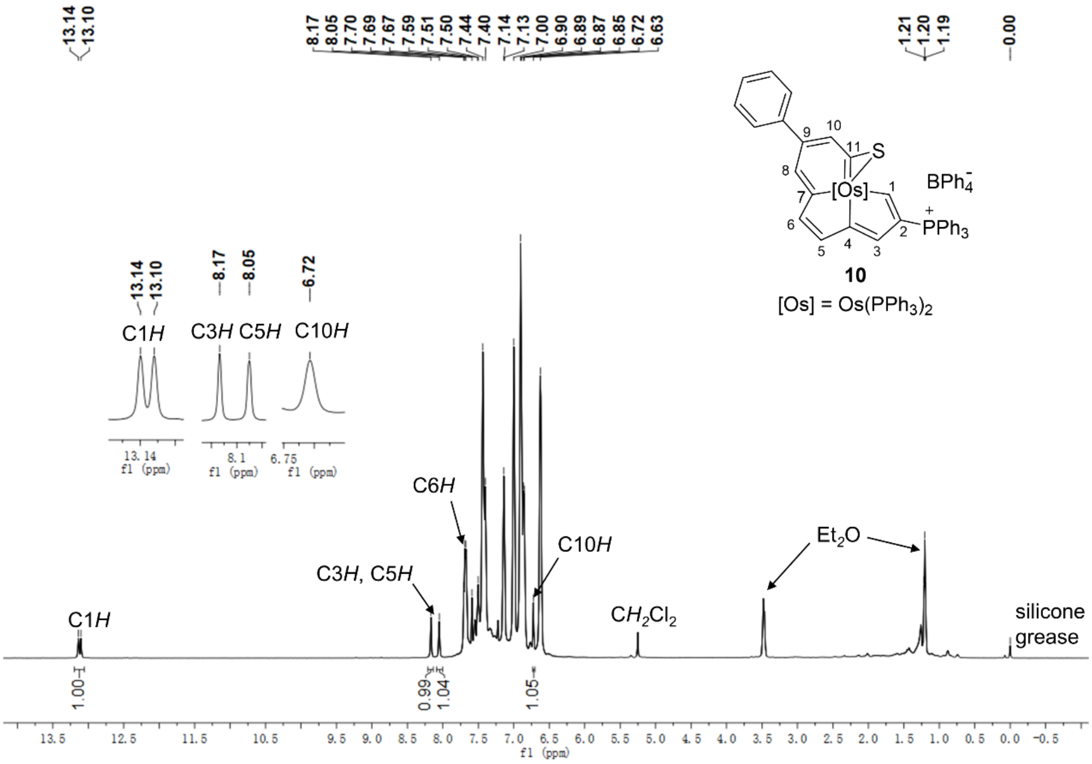

Supplementary Figure 47. The ^{1}H NMR (600 MHz, $CDCl_3$) spectrum for **10**.

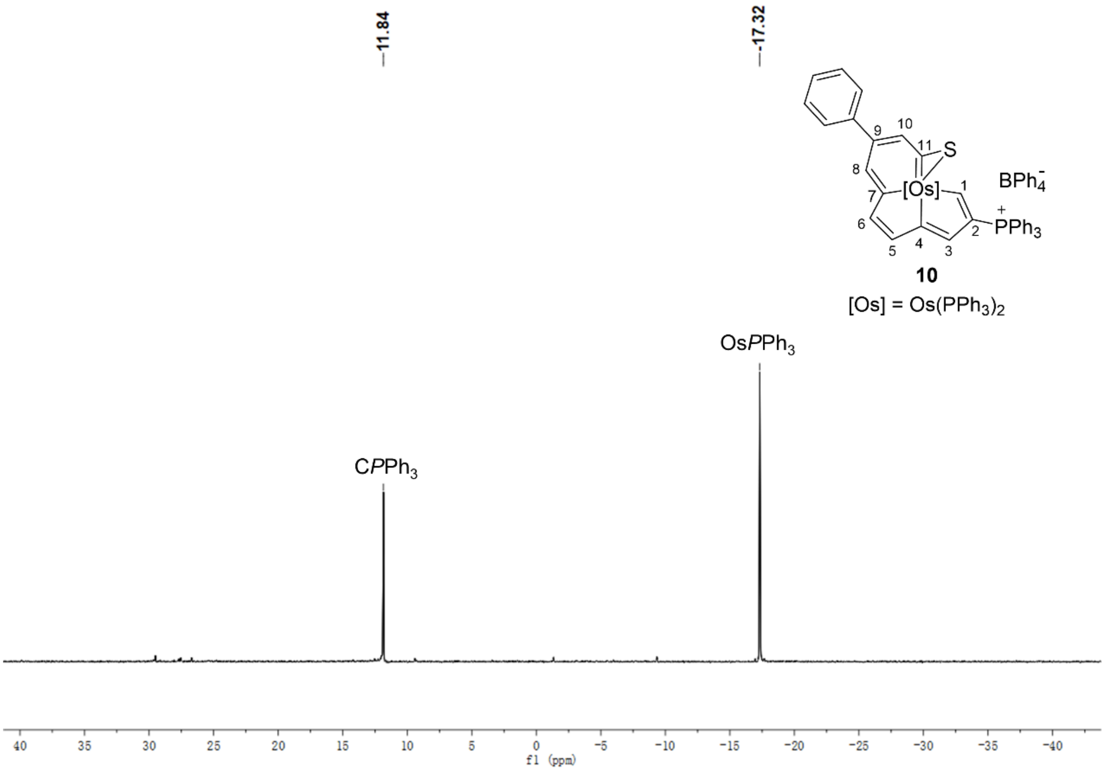

Supplementary Figure 48. The ^{31}P{^{1}H} NMR (243 MHz, $CDCl_3$) spectrum for **10**.

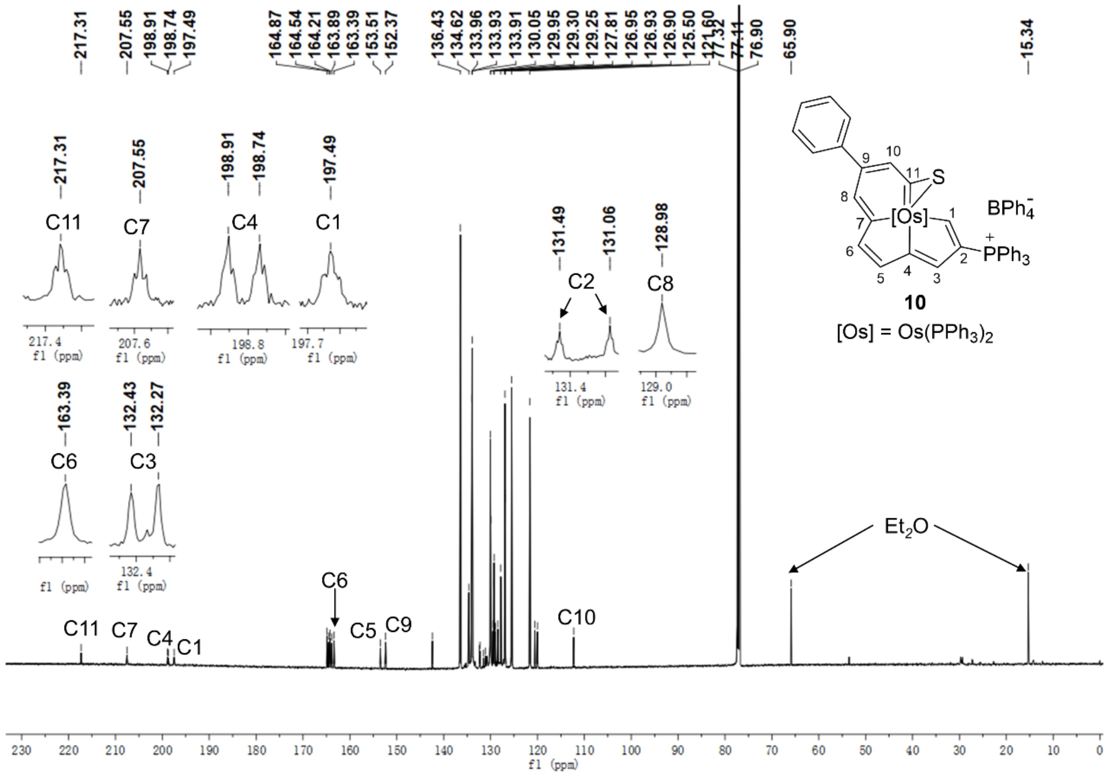

Supplementary Figure 49. The $^{13}C\{^{1}H\}$ NMR (151 MHz, $CDCl_3$) spectrum for **10**.

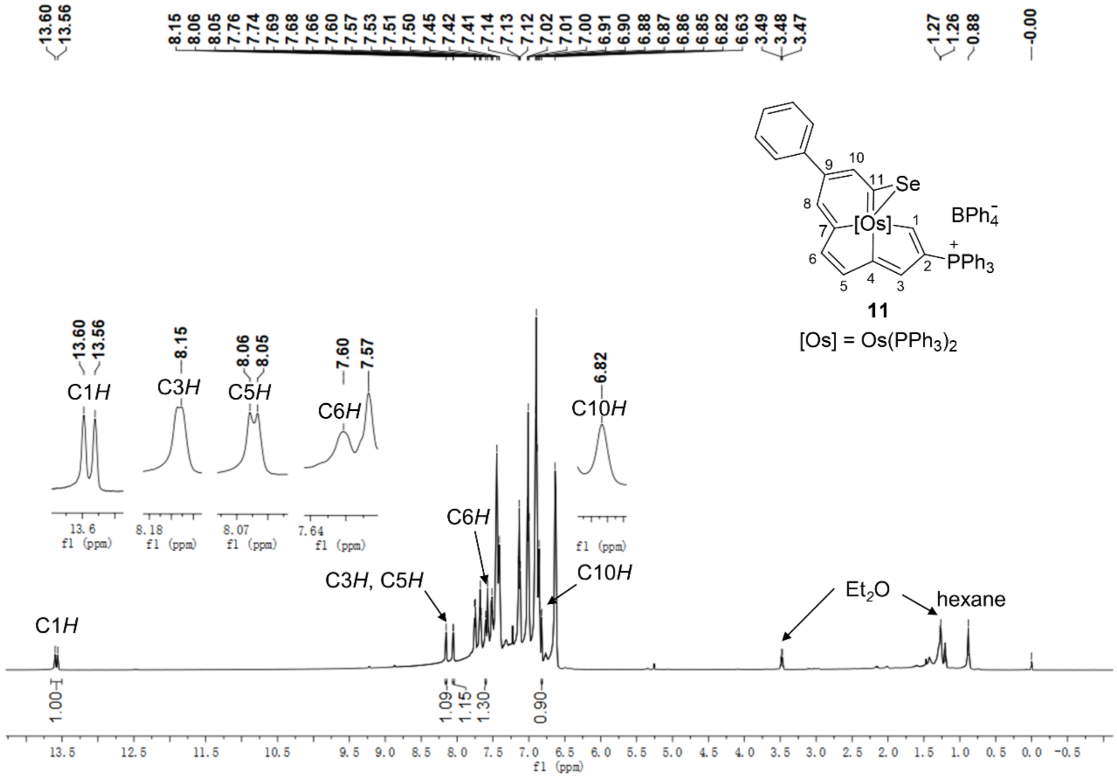

Supplementary Figure 50. The ^{1}H NMR (600 MHz, $CDCl_3$) spectrum for **11**.

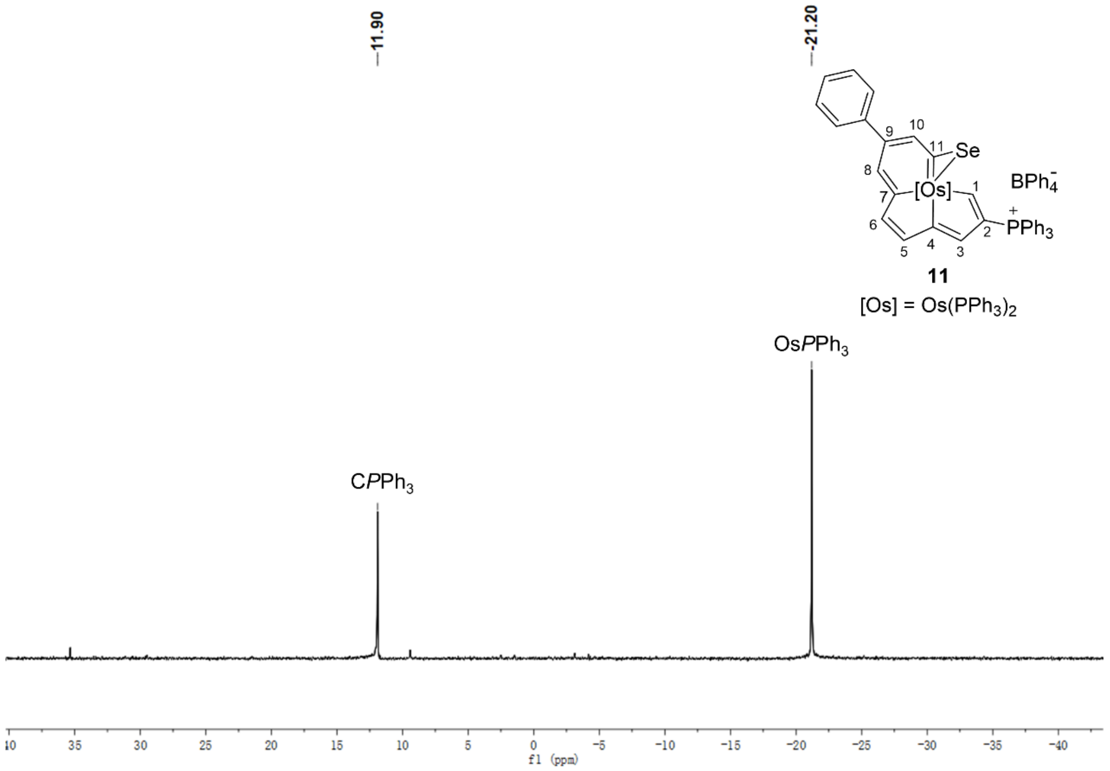

Supplementary Figure 51. The $^{31}P\{^{1}H\}$ NMR (243 MHz, $CDCl_3$) spectrum for **11**.

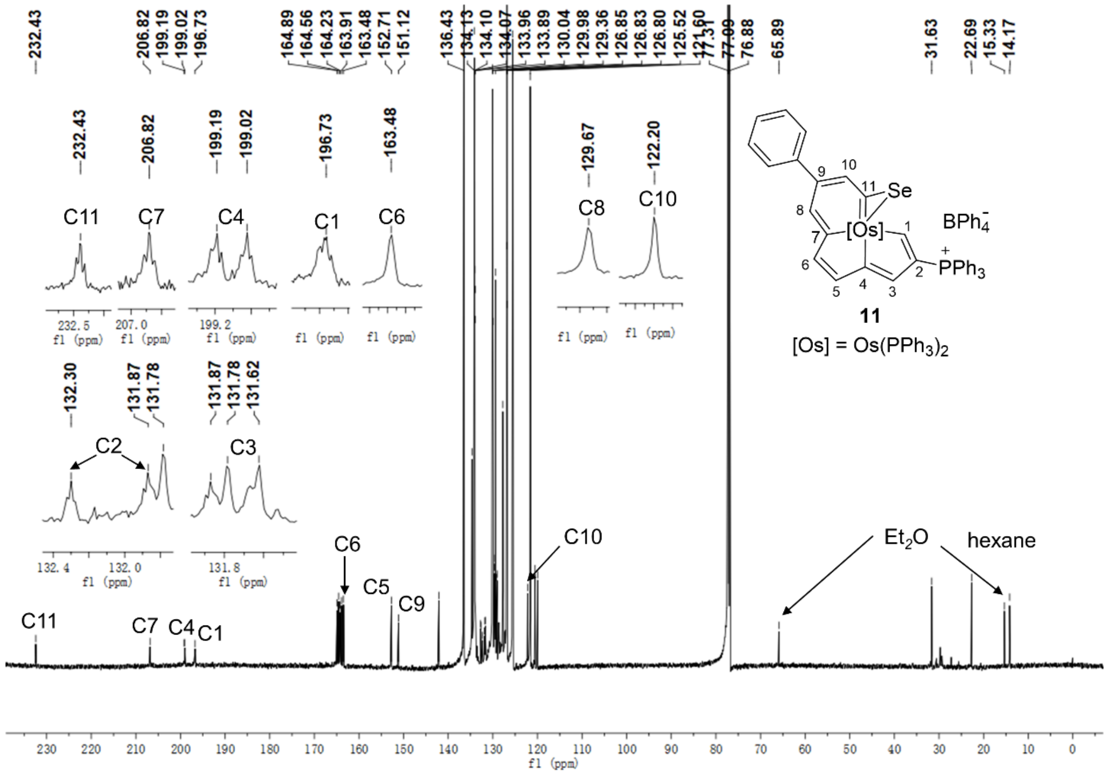

Supplementary Figure 52. The $^{13}C\{^{1}H\}$ NMR (151 MHz, $CDCl_3$) spectrum for **11**.

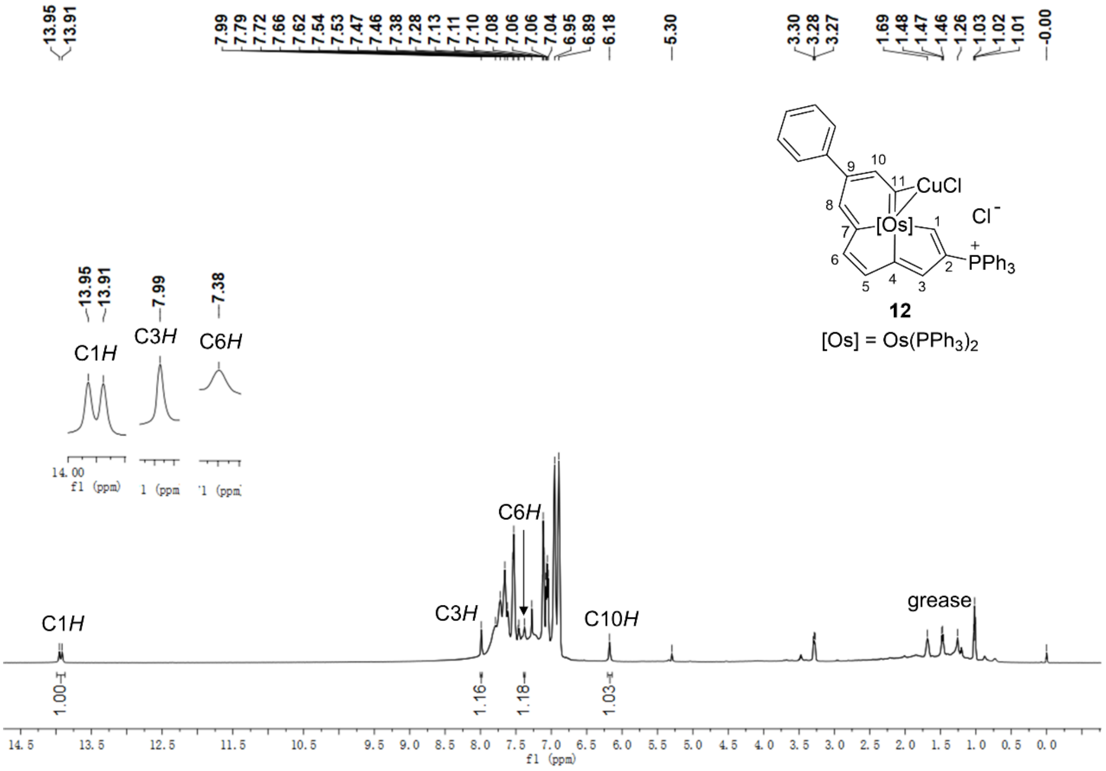

Supplementary Figure 53. The ^{1}H NMR (600 MHz, $CDCl_3$) spectrum for **12**.

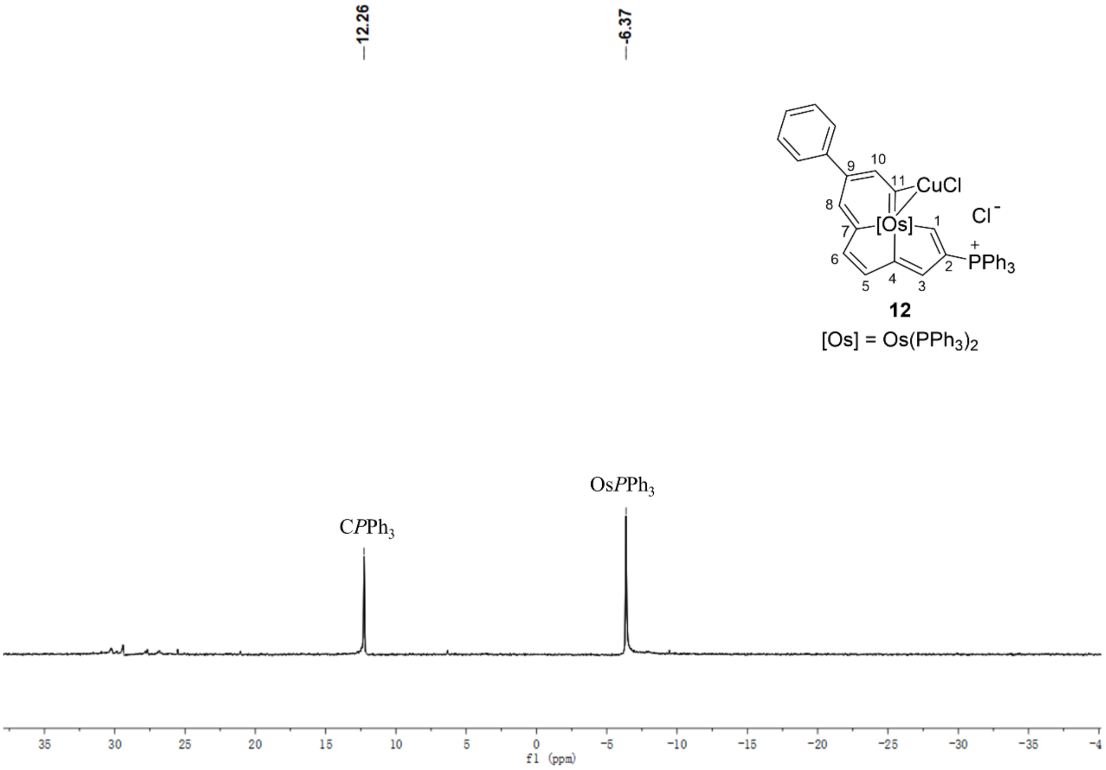

Supplementary Figure 54. The ^{31}P{^{1}H} NMR (243 MHz, $CDCl_3$) spectrum for **12**.

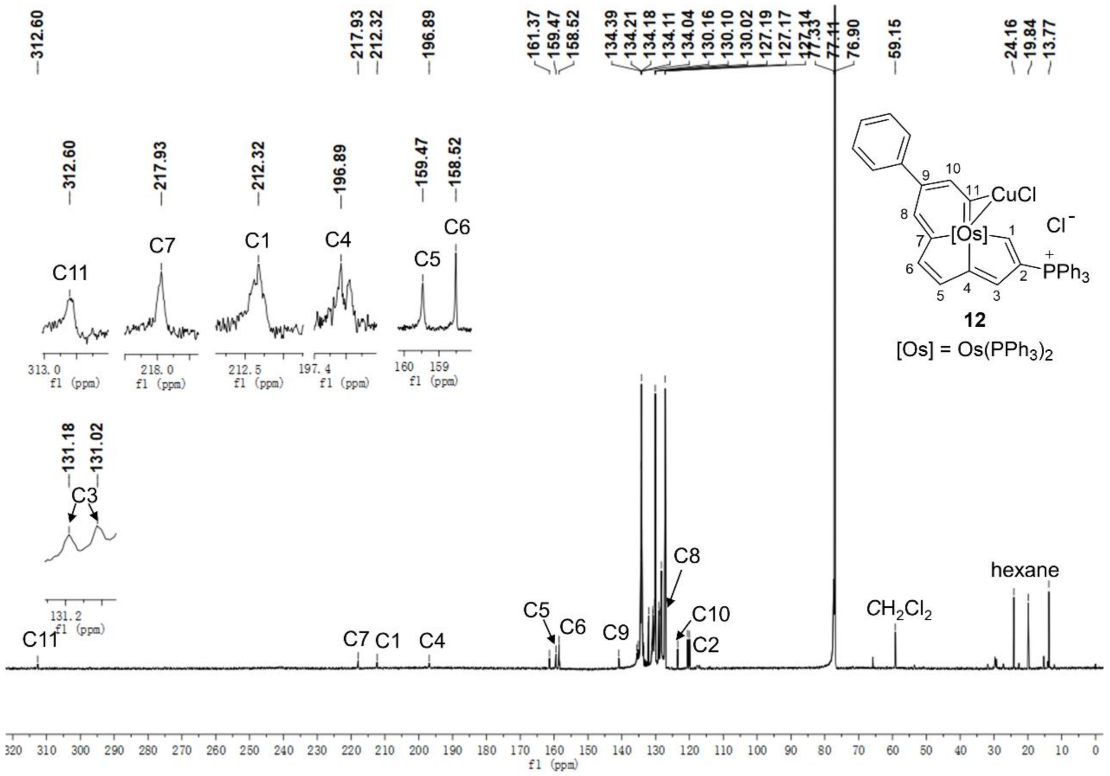

Supplementary Figure 55. The $^{13}C\{^{1}H\}$ NMR (151 MHz, $CDCl_3$) spectrum for **12**.

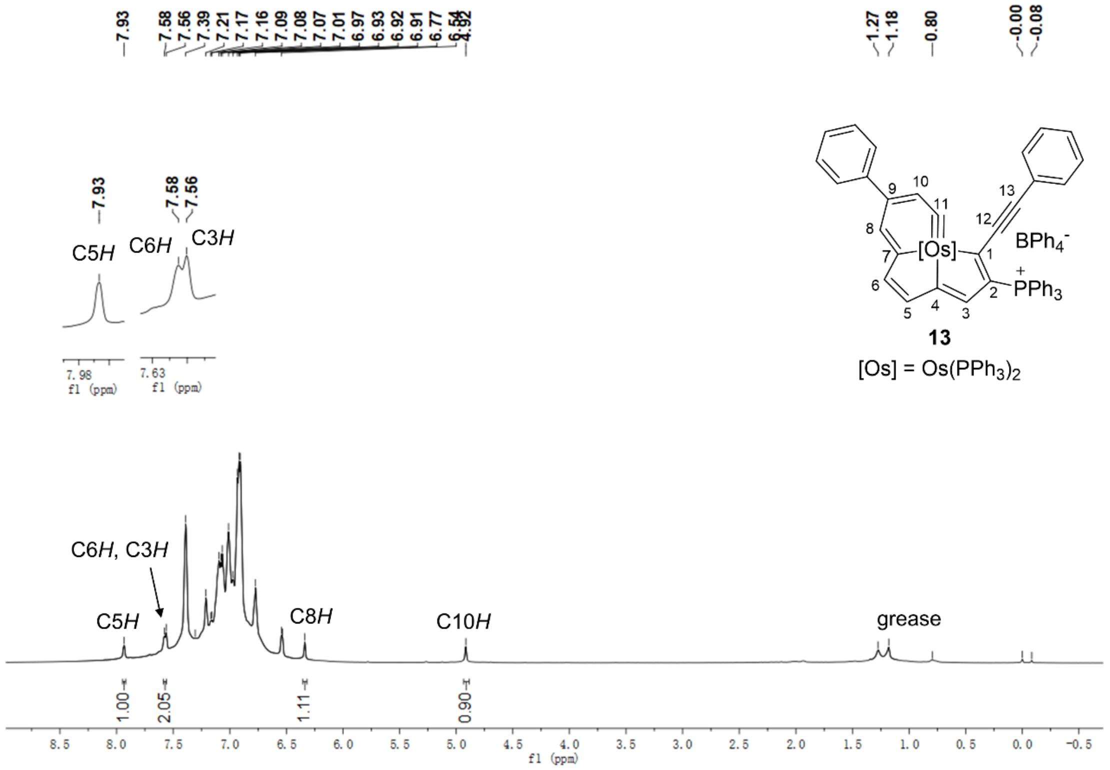

Supplementary Figure 56. The ^{1}H NMR (600 MHz, $CDCl_3$) spectrum for **13**.

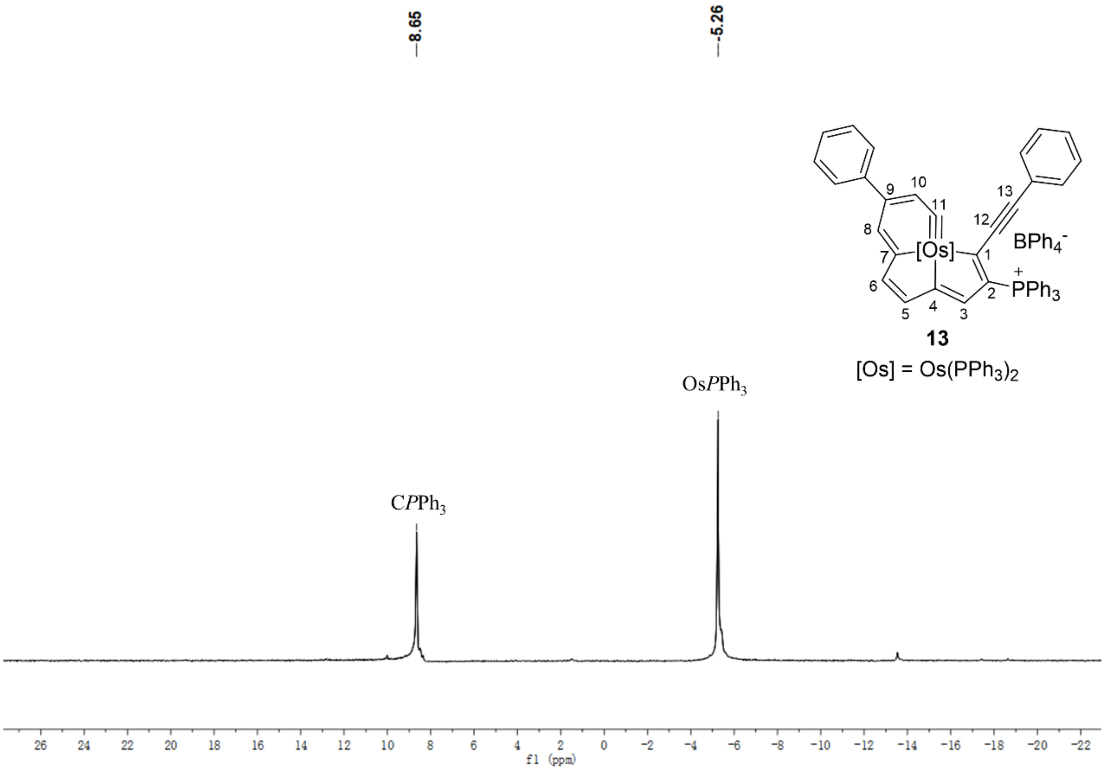

Supplementary Figure 57. The $^{31}P\{^{1}H\}$ NMR (243 MHz, $CDCl_3$) spectrum for **13**.

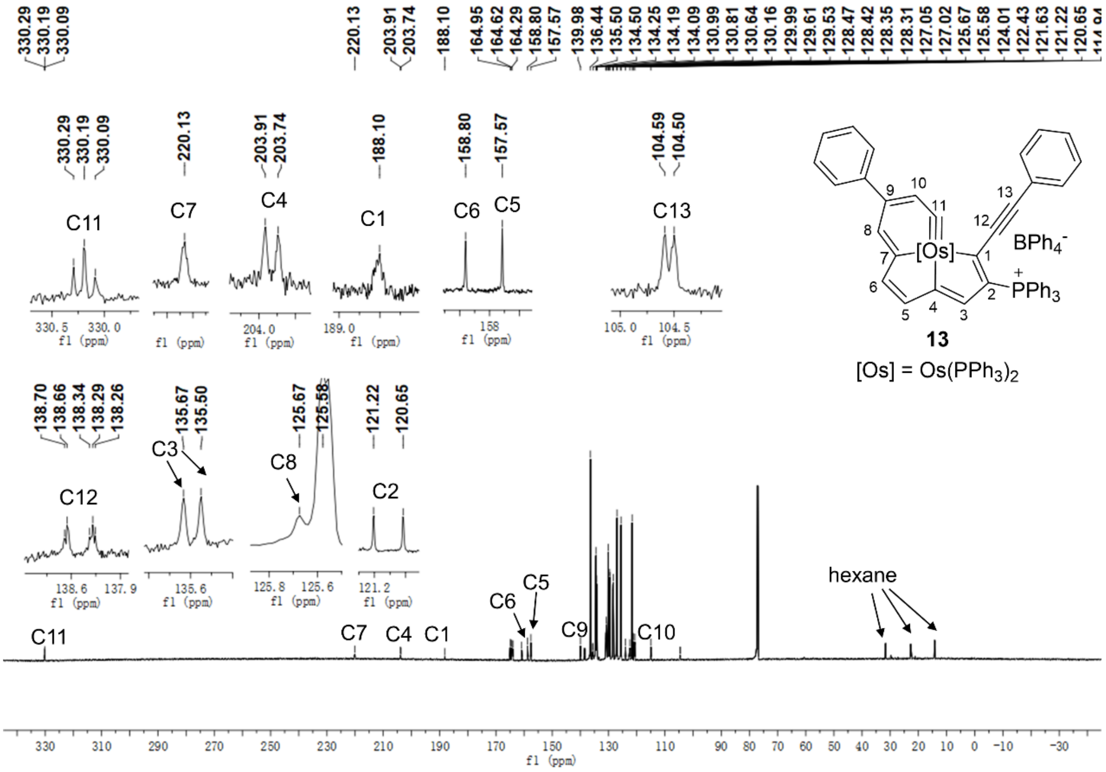

Supplementary Figure 58. The $^{13}C\{^{1}H\}$ NMR (151 MHz, $CDCl_3$) spectrum for **13**.

6. HRMS spectra

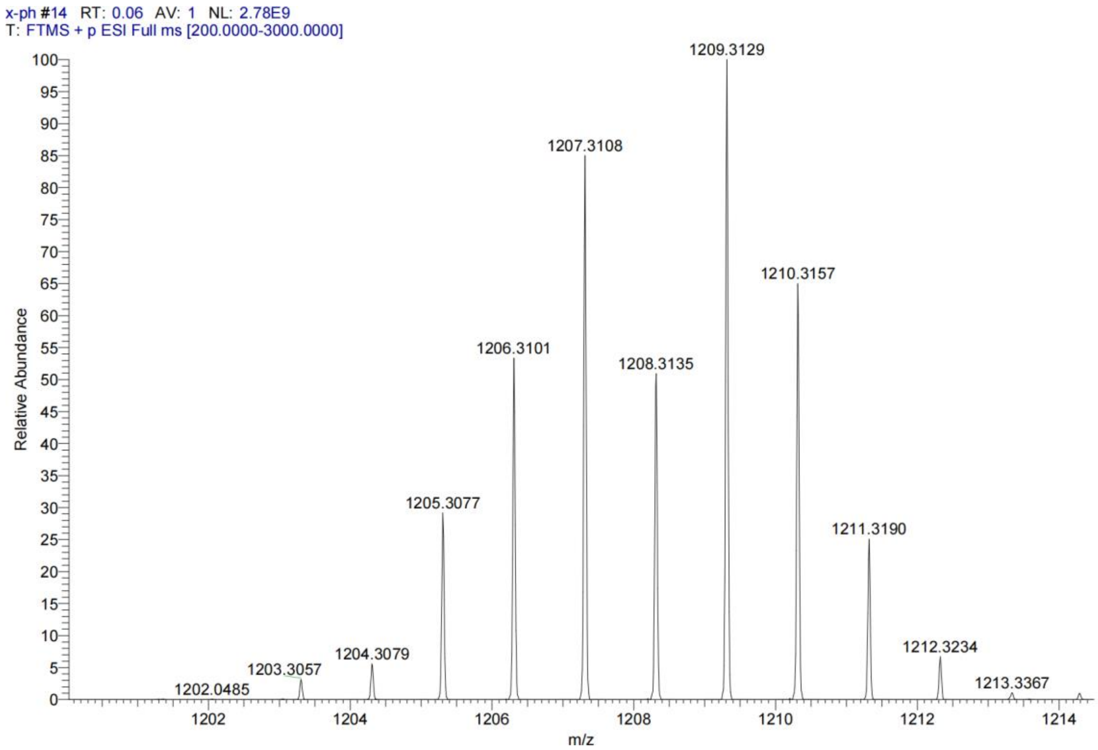

Supplementary Figure 59. Positive ion ESI-MS spectrum for complex **3a** $[C_{71}H_{56}OOsP_3]^+$ measured in dichloromethane.

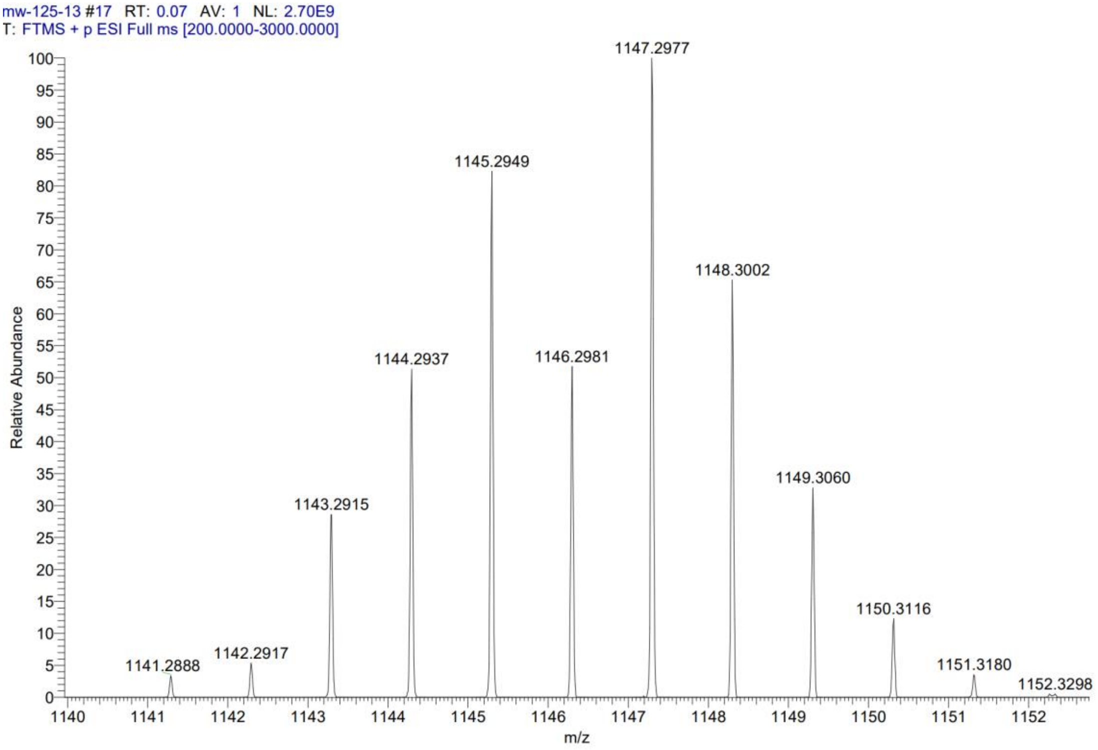

Supplementary Figure 60. Positive ion ESI-MS spectrum for complex **3b** $[C_{66}H_{54}OOsP_3]^+$ measured in dichloromethane.

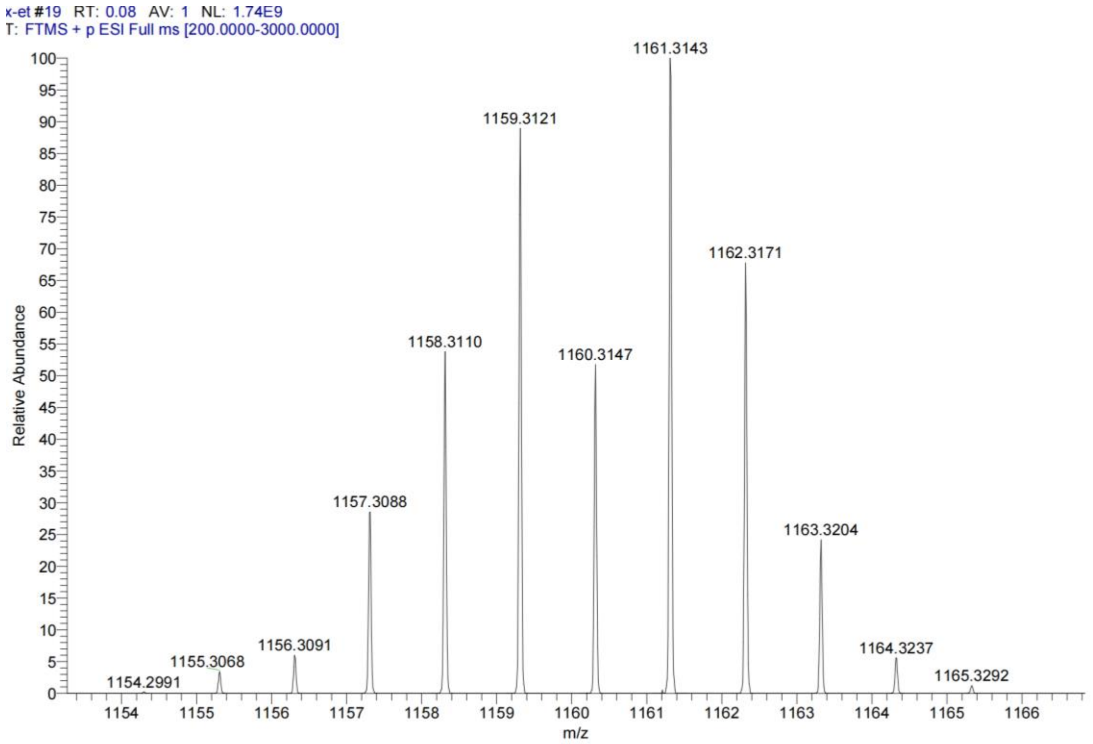

Supplementary Figure 61. Positive ion ESI-MS spectrum for complex **3c** $[C_{67}H_{56}OOsP_3]^+$ measured in dichloromethane.

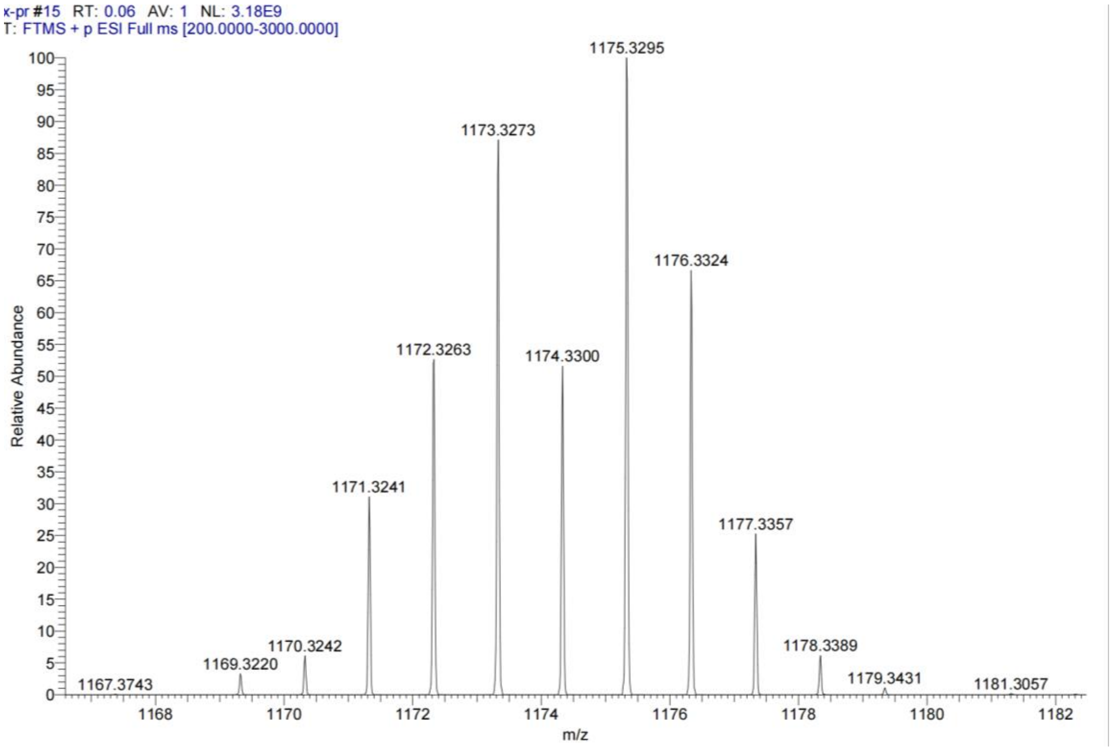

Supplementary Figure 62. Positive ion ESI-MS spectrum for complex **3d** $[C_{68}H_{58}OOsP_3]^+$ measured in dichloromethane.

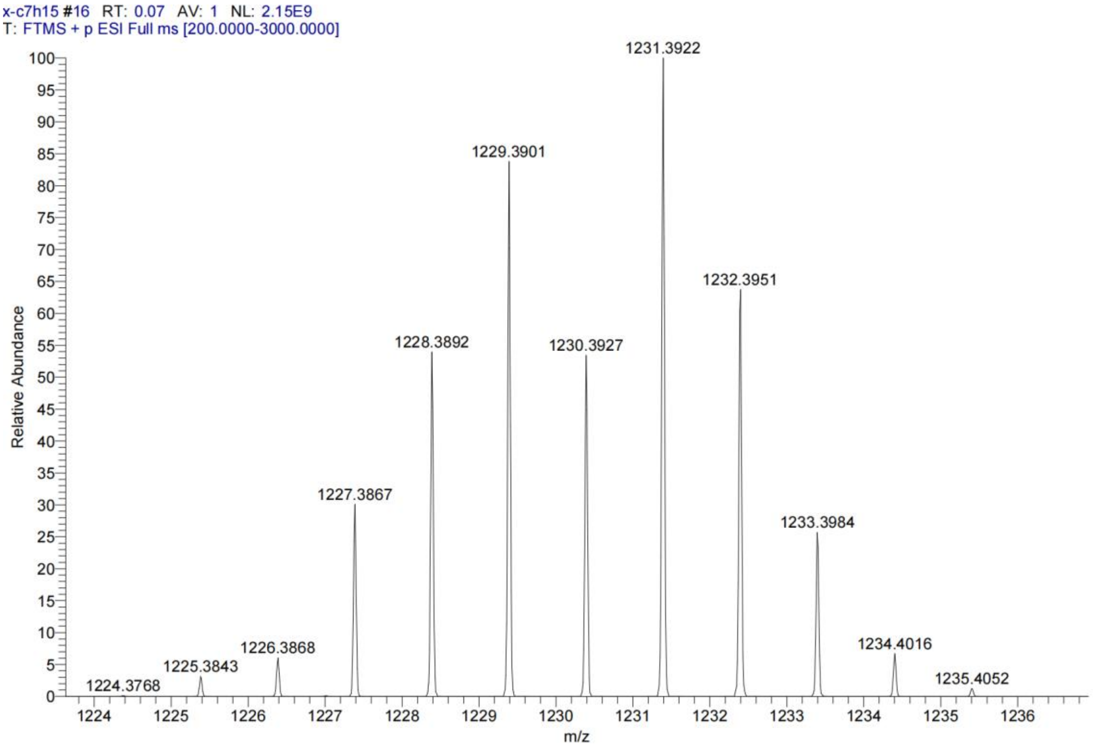

Supplementary Figure 63. Positive ion ESI-MS spectrum for complex **3e** $[C_{72}H_{66}OOsP_3]^+$ measured in dichloromethane.

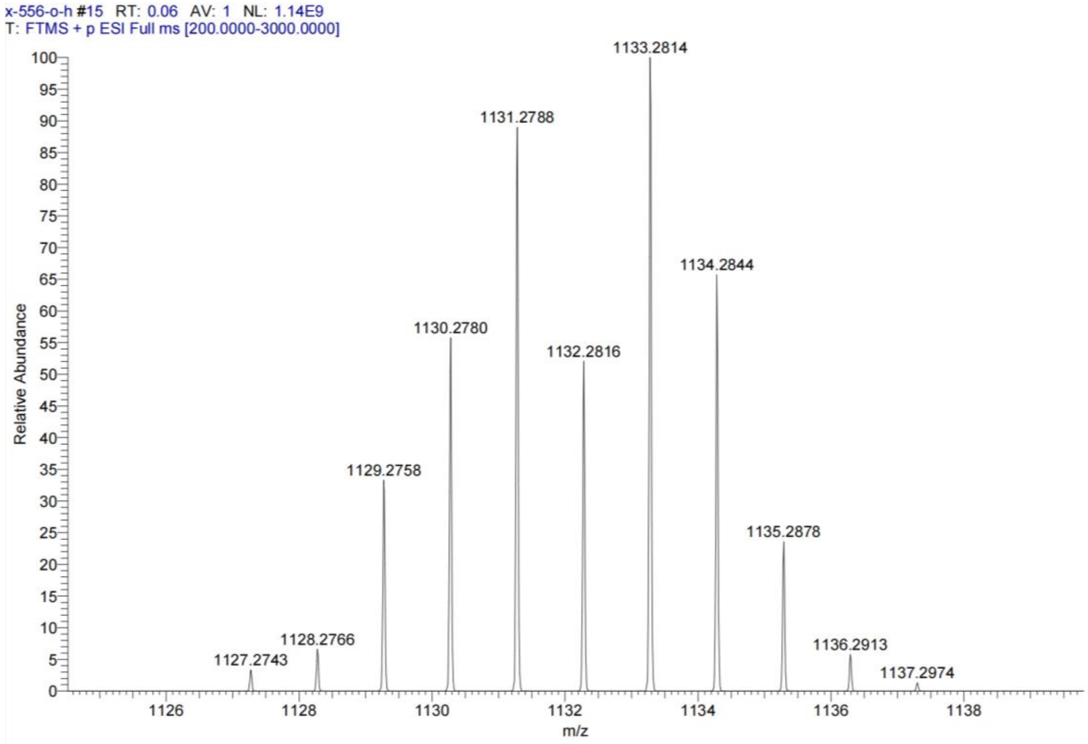

Supplementary Figure 64. Positive ion ESI-MS spectrum for complex **3f** $[C_{65}H_{52}OOsP_3]^+$ measured in dichloromethane.

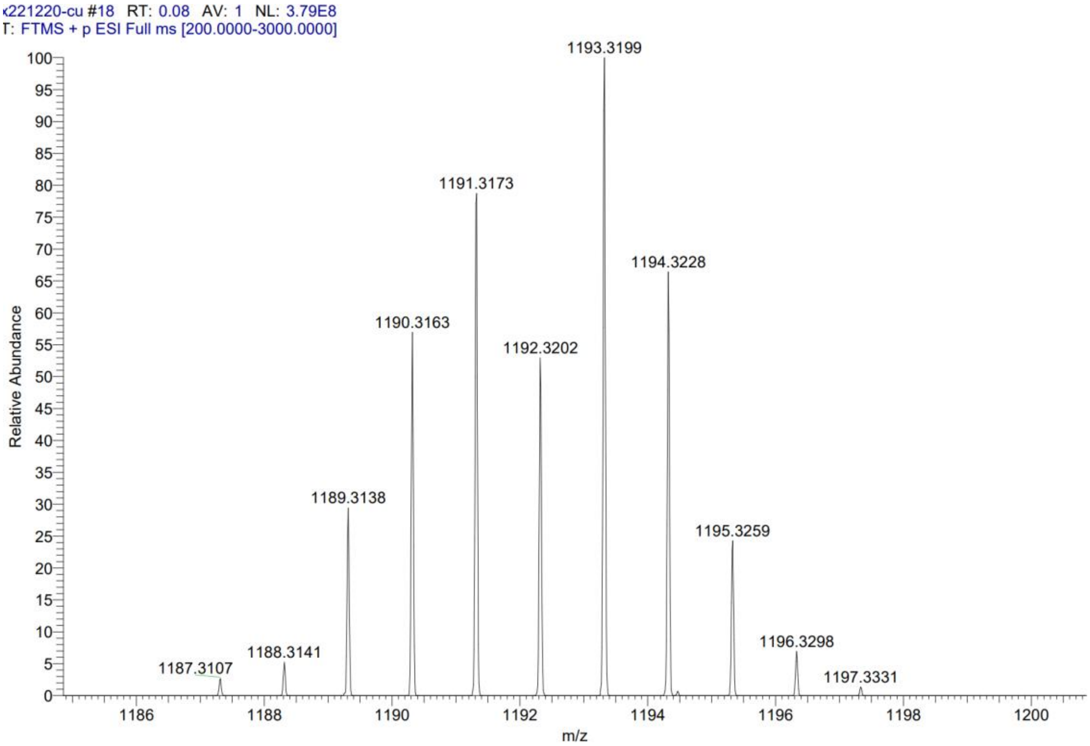

Supplementary Figure 65. Positive ion ESI-MS spectrum for complex **4a** $[C_{71}H_{56}OsP_3]^+$ measured in dichloromethane.

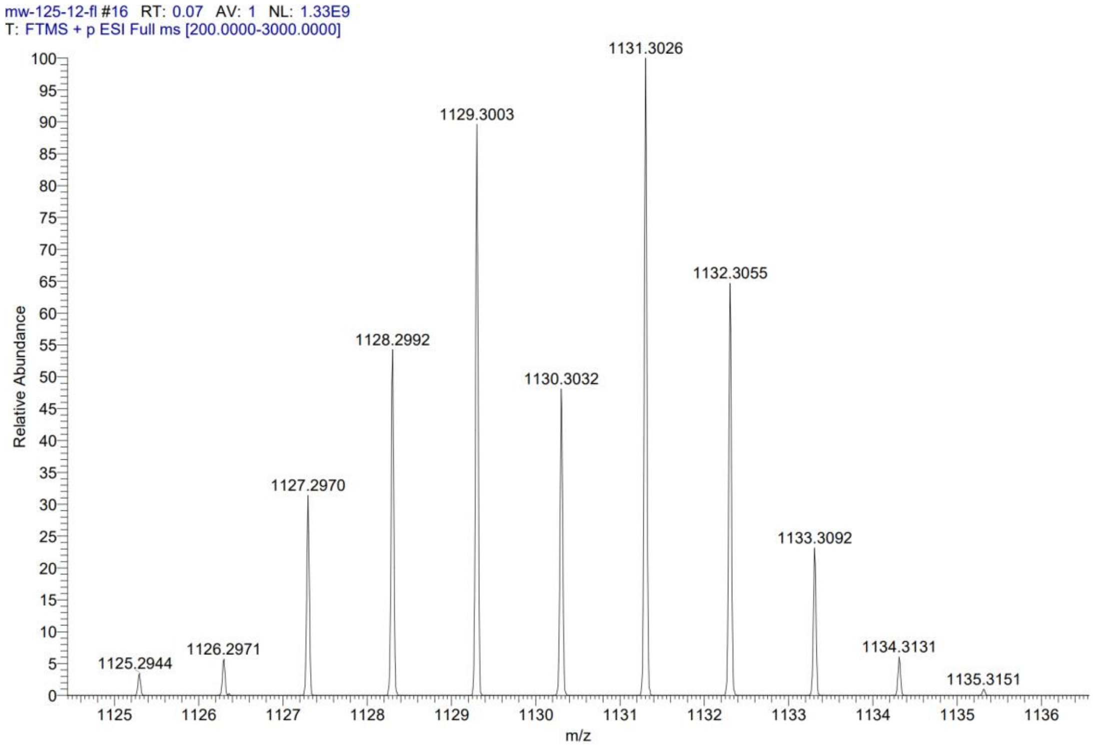

Supplementary Figure 66. Positive ion ESI-MS spectrum for complex **4b-BPh$_4$** $[C_{66}H_{54}OsP_3]^+$ measured in dichloromethane.

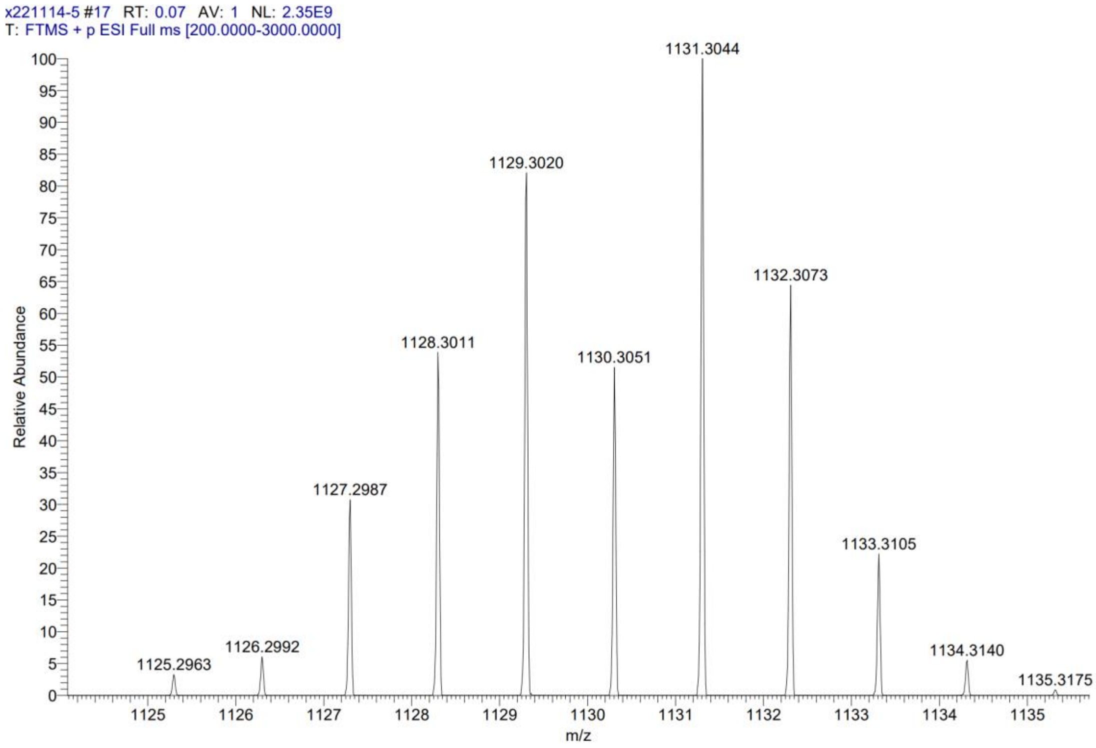

Supplementary Figure 67. Positive ion ESI-MS spectrum for complex **4b-Cl** $[C_{66}H_{54}OsP_3]^+$ measured in dichloromethane.

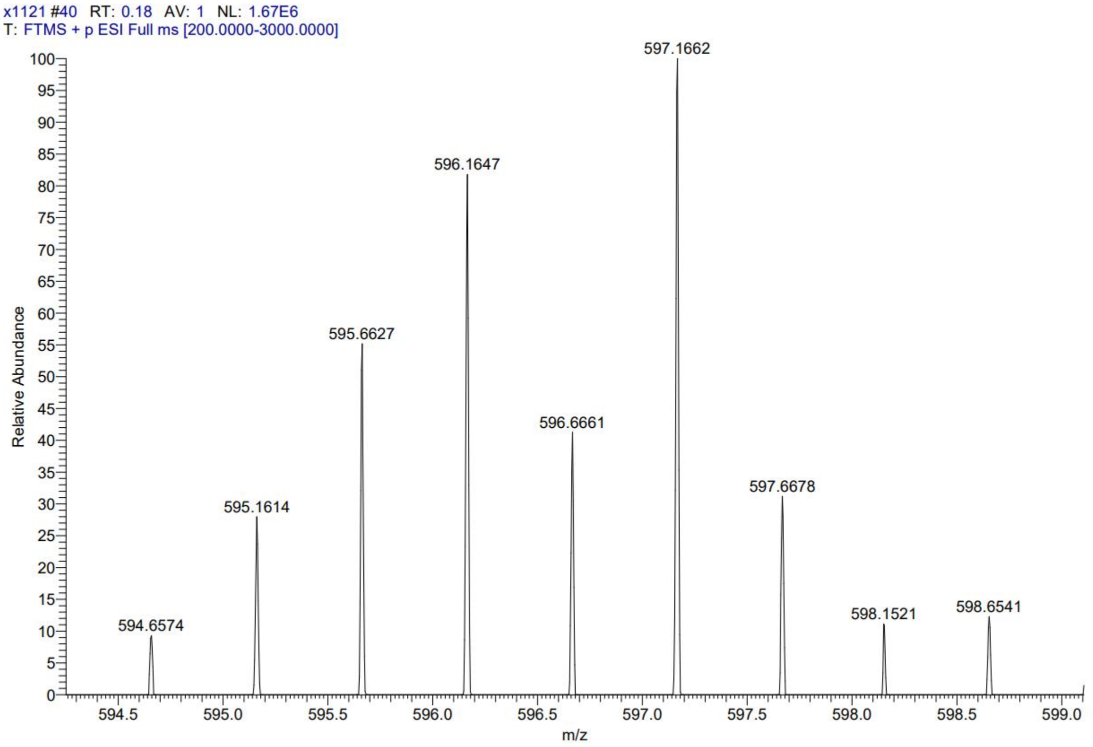

Supplementary Figure 68. Positive ion ESI-MS spectrum for complex **5** $[C_{71}H_{57}OsP_3]^{2+}$ measured in dichloromethane.

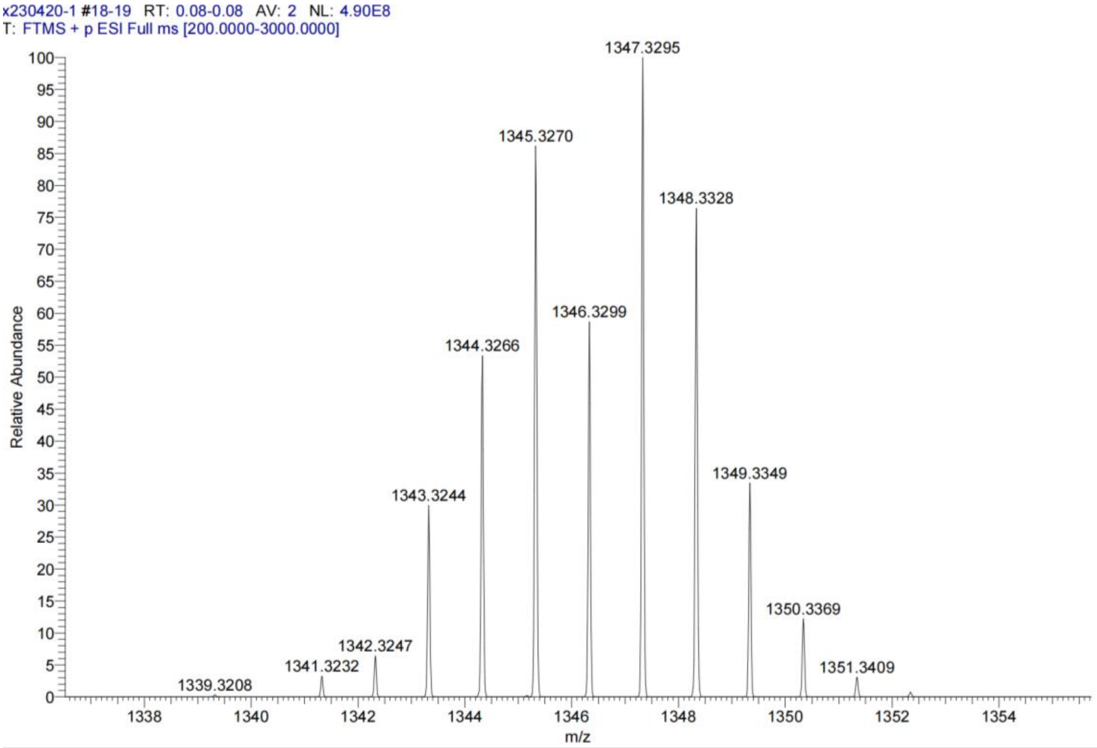

Supplementary Figure 69. Positive ion ESI-MS spectrum for complex **6** $[C_{78}H_{62}O_2OsP_3S]^+$ measured in dichloromethane.

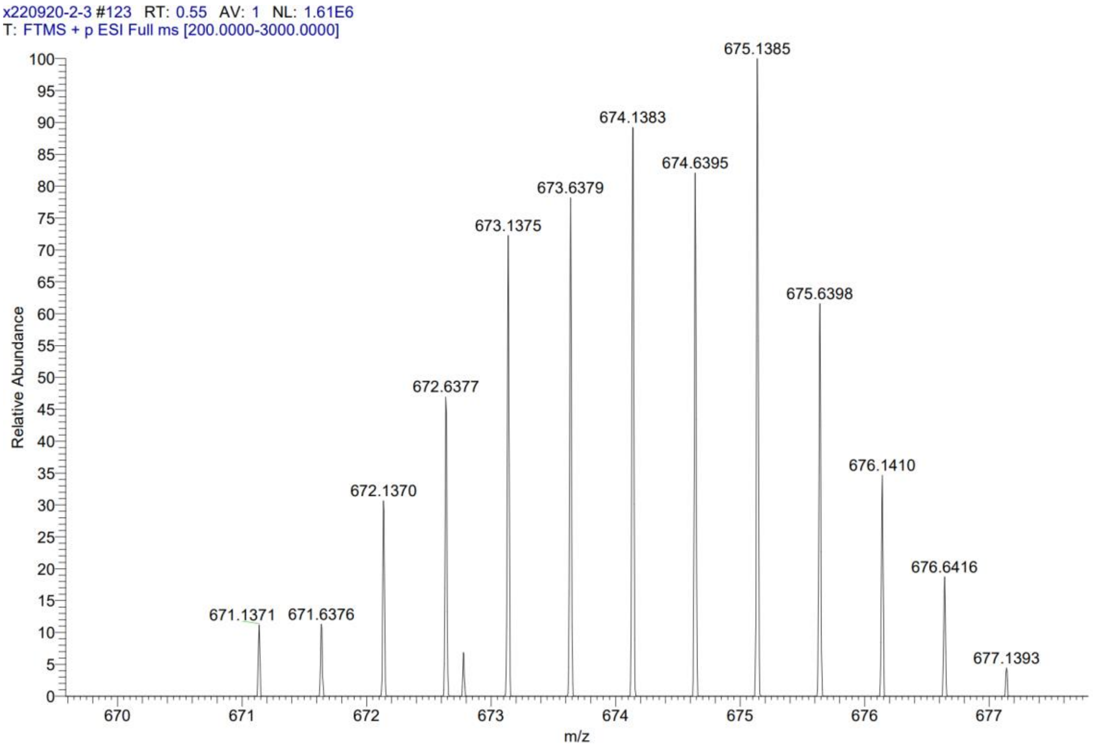

Supplementary Figure 70. Positive ion ESI-MS spectrum for complex **7** $[C_{77}H_{61}OsP_3Se]^{2+}$ measured in dichloromethane.

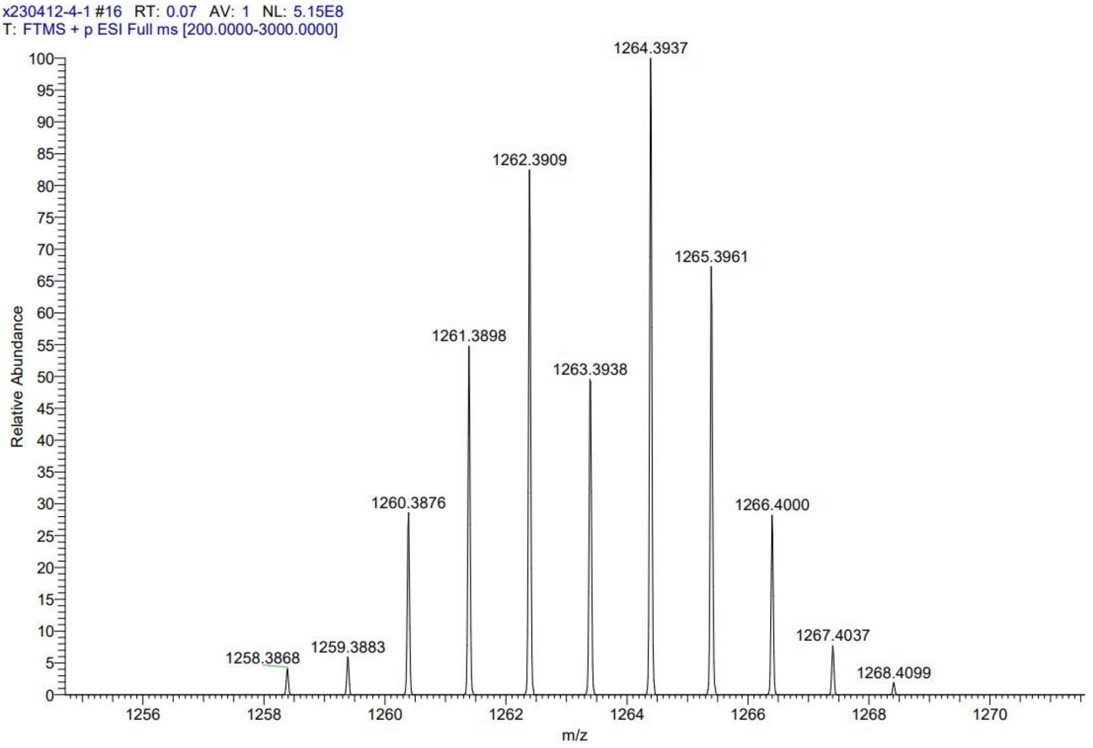

Supplementary Figure 71. Positive ion ESI-MS spectrum for complex **8** $[C_{75}H_{65}NOsP_3]^{+}$ measured in dichloromethane.

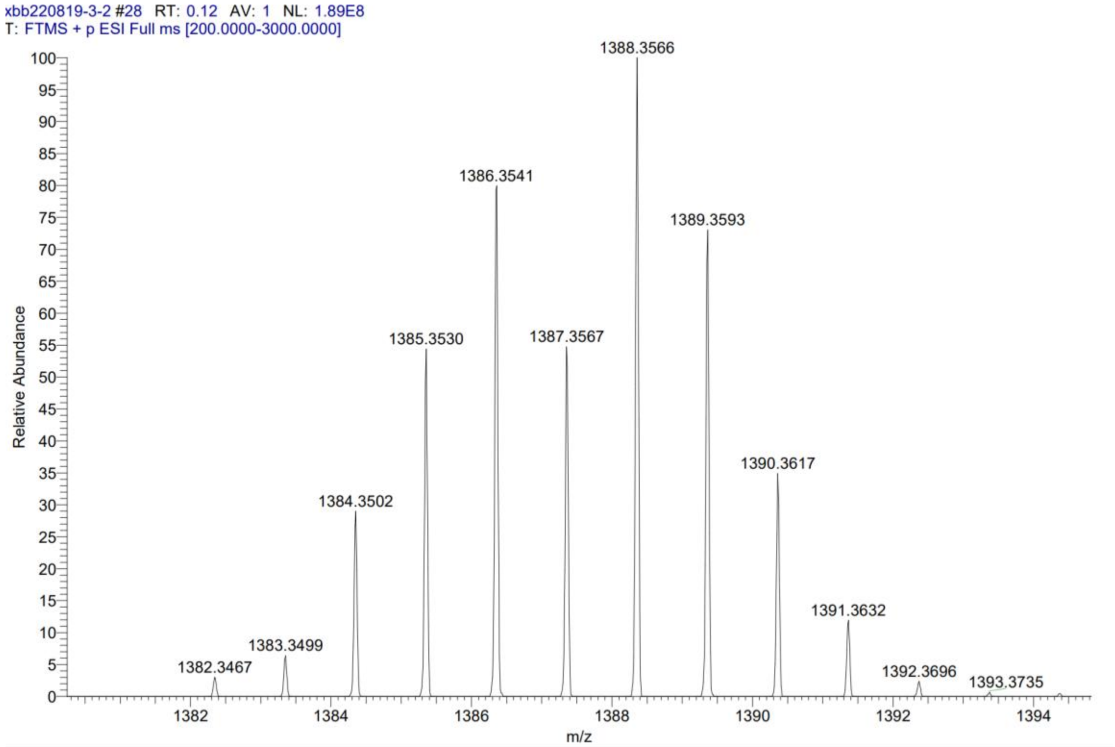

Supplementary Figure 72. Positive ion ESI-MS spectrum for complex **9** $[C_{80}H_{65}NO_2OsP_3S]^+$ measured in dichloromethane.

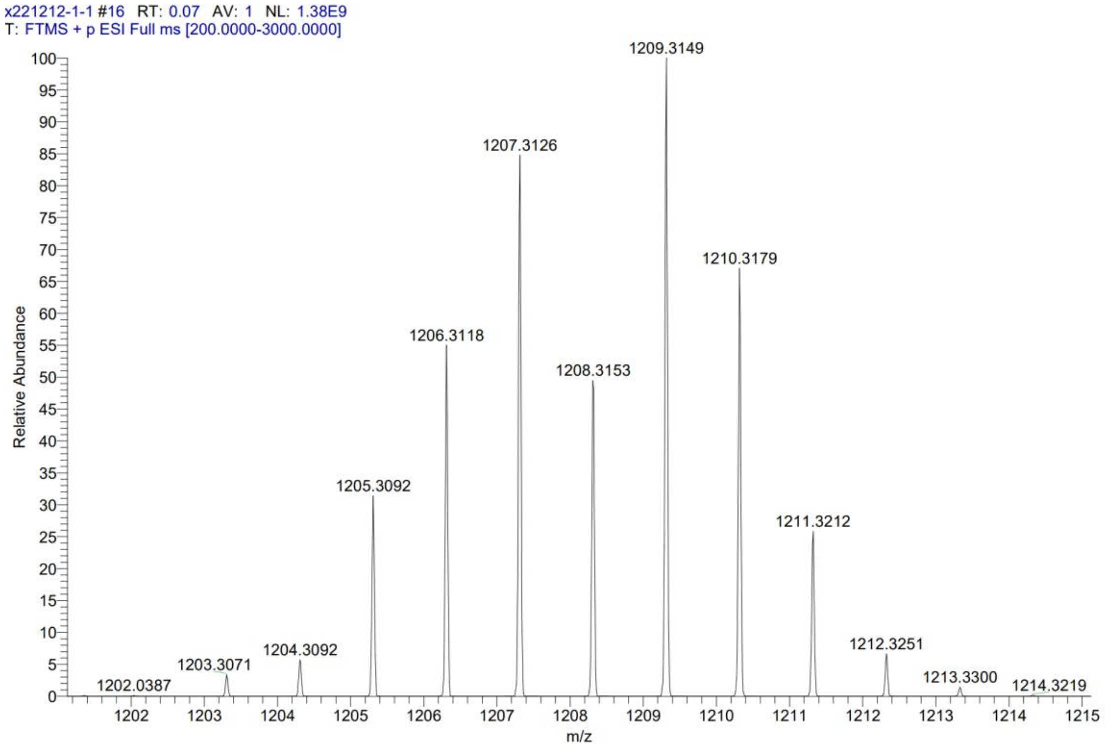

Supplementary Figure 73. Positive ion ESI-MS spectrum for complex **3a-BPh**$_4$ $[C_{71}H_{56}OOsP_3]^+$ measured in dichloromethane.

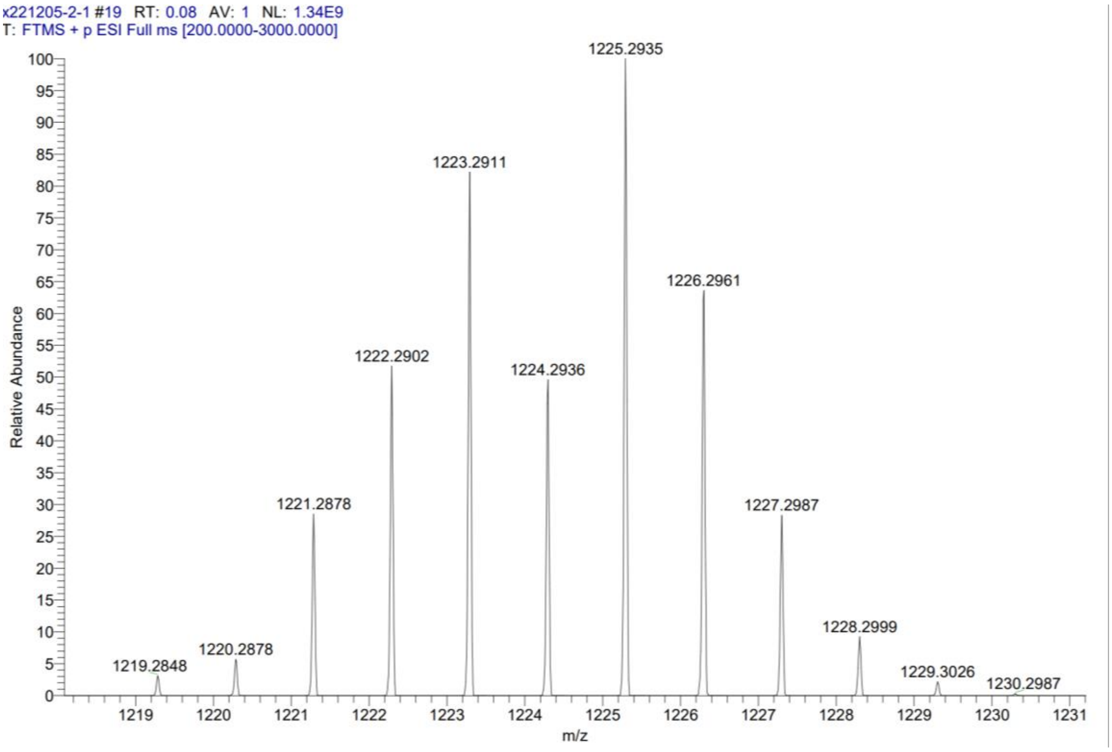

Supplementary Figure 74. Positive ion ESI-MS spectrum for complex **10** $[C_{71}H_{56}OsP_3S]^+$ measured in dichloromethane.

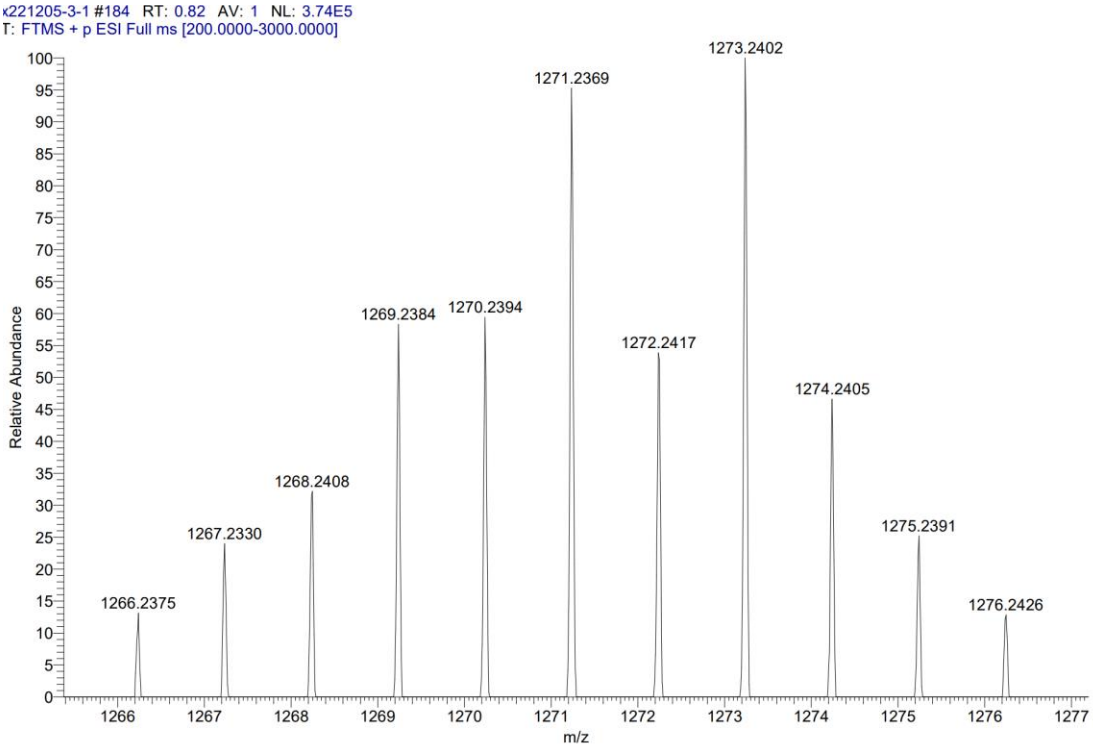

Supplementary Figure 75. Positive ion ESI-MS spectrum for complex **11** $[C_{71}H_{56}OsP_3Se]^+$ measured in dichloromethane.

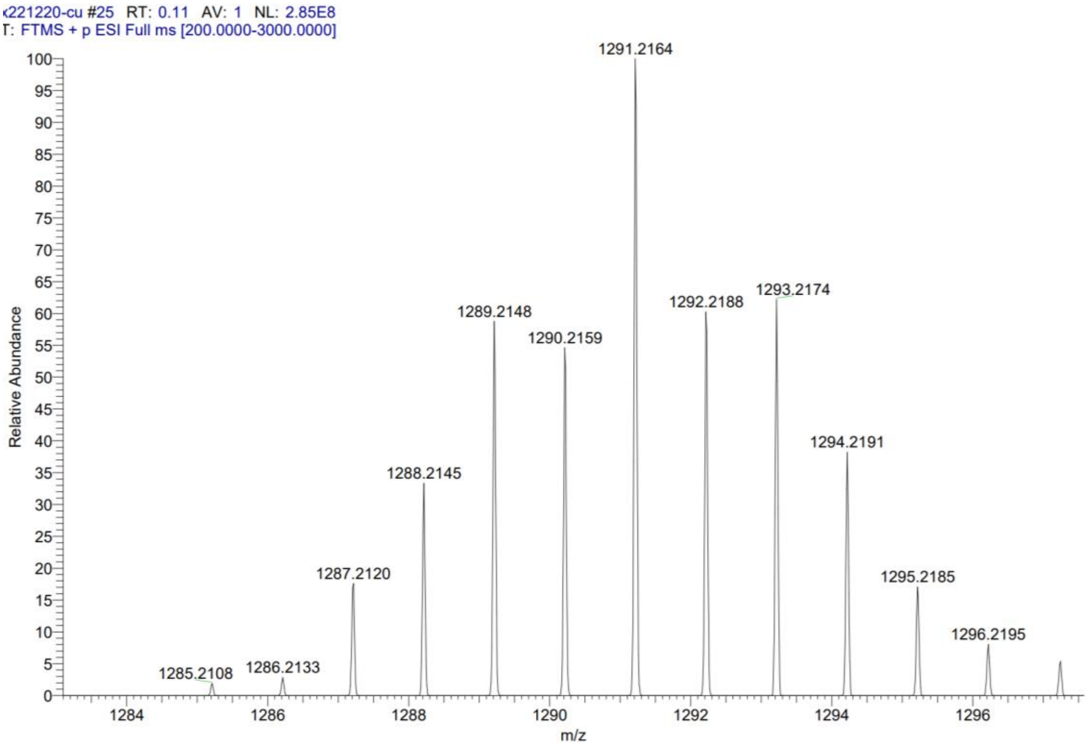

Supplementary Figure 76. Positive ion ESI-MS spectrum for complex **12** $[C_{71}H_{56}ClCuOsP_3]^+$ measured in dichloromethane.

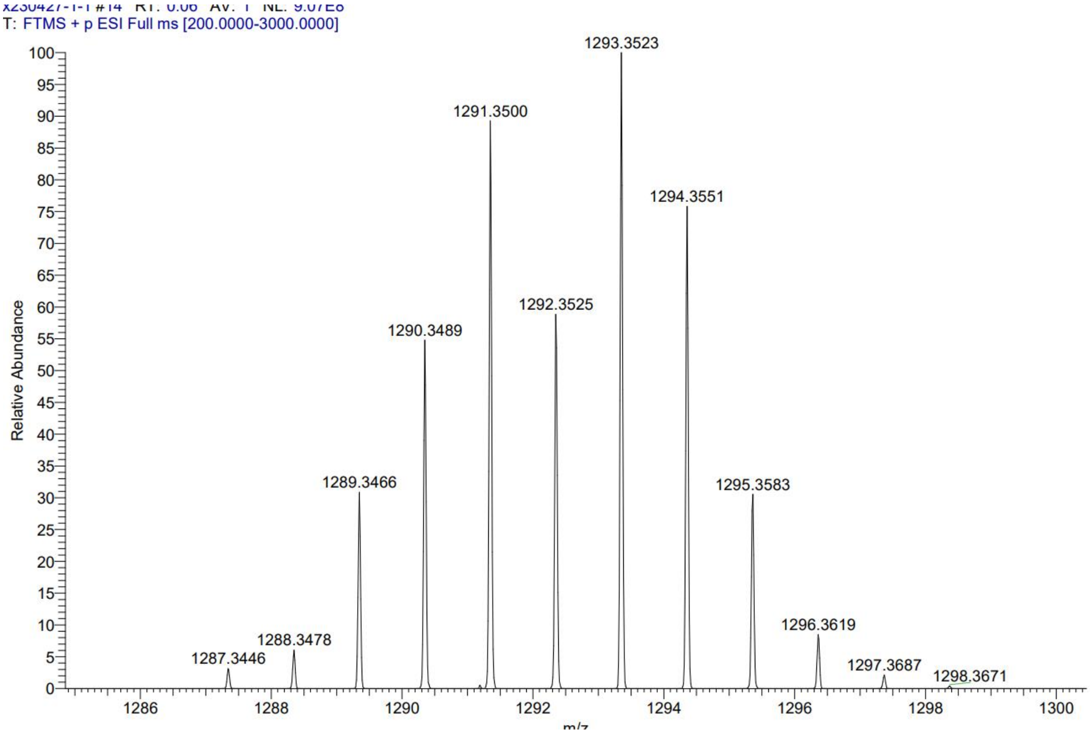

Supplementary Figure 77. Positive ion ESI-MS spectrum for complex **13** $[C_{79}H_{60}OsP_3]^+$ measured in dichloromethane.

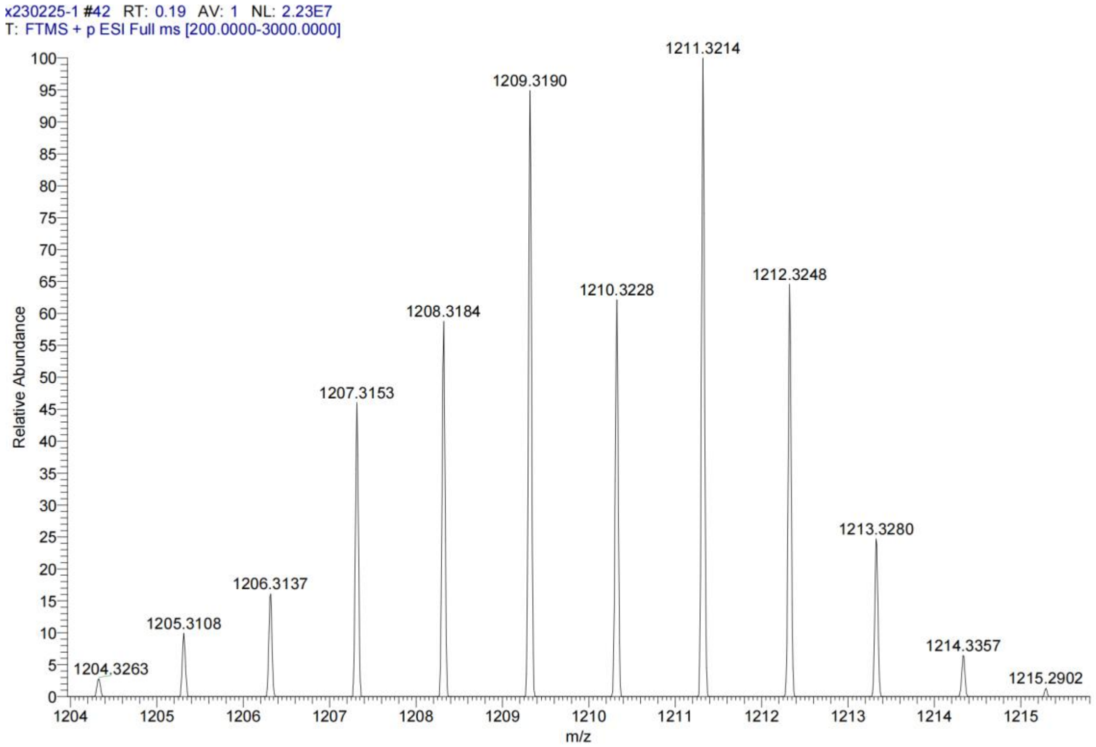

Supplementary Figure 78. Positive ion ESI-MS spectrum for complex **^{18}O-3a** $[C_{71}H_{56}{}^{18}OOsP_3]^+$ measured in dichloromethane.

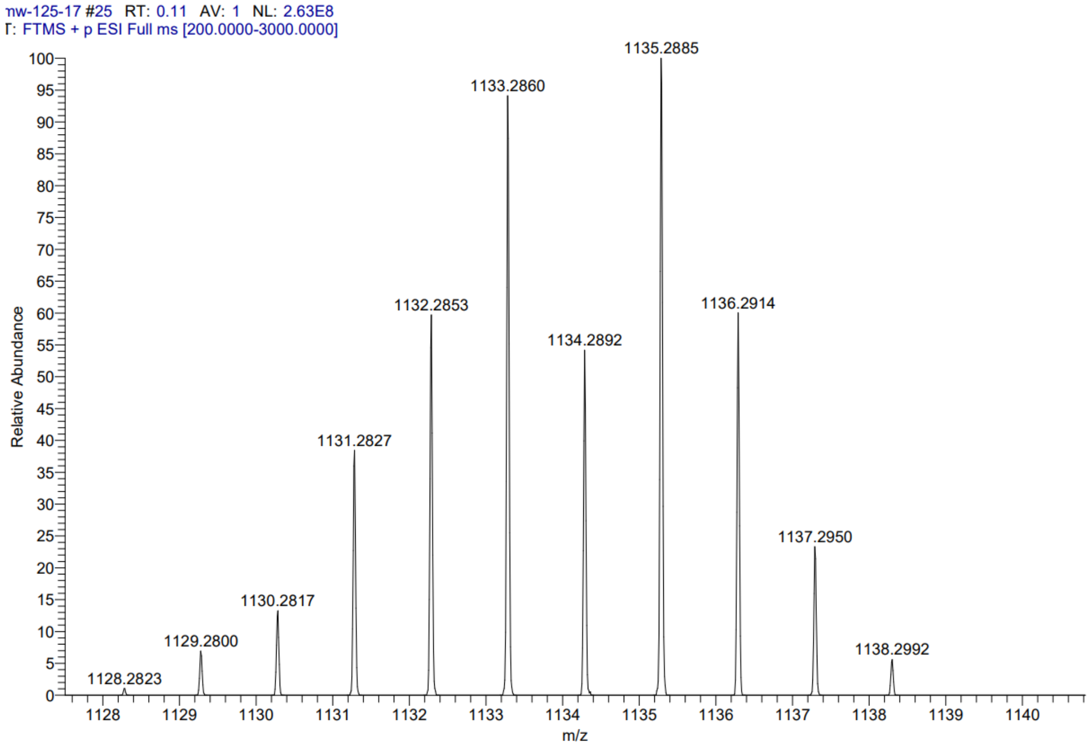

Supplementary Figure 79. Positive ion ESI-MS spectrum for complex **^{18}O-3f** $[C_{65}H_{52}{}^{18}OOsP_3]^+$ measured in dichloromethane.

7. Crystallographic details

Crystallographic details. Single-crystal X-ray diffraction data were collected on a Bruker CMOs area detector with monochromated Cu Kα radiation (λ = 1.54178 Å). All the Data were corrected for absorption effects using the multi-scan technique. Using Olex2[2], the structures were solved with the ShelXT[3] structure solution program using Charge Flipping and refined with the ShelXL[4] refinement package using Least Squares minimization. Non-H atoms were refined anisotropically unless otherwise stated. Hydrogen atoms were introduced at their geometric positions and refined as riding atoms unless otherwise stated. The contribution of disordered solvents was removed from the dataset using the SOLVENT MASK routine of Olex 2 which was reported in the CIF. The crystals suitable for X-ray diffraction were grown from the CH_2Cl_2 solution layered with *n*-hexane for complexes **3a**, **3c**, **4b-Cl**, **7** and **10**; and grown from the $CHCl_3$ solution for **6**, **11** and **12**. CCDC-2244936 (**3a**), CCDC-2244956 (**3c**), CCDC-2244958 (**4b-Cl**), CCDC-2260544 (**6**), CCDC-2244973 (**7**), CCDC- 2251588 (**10**), CCDC-2244957 (**11**), and CCDC-2244972 (**12**), contain the supplementary crystallographic data for this paper, and the data can be obtained free of charge from The Cambridge Crystallographic Data Centre via www.ccdc.cam.ac.uk/structures. For further details on the crystal data, data collection, and refinements, see **Supplementary Tables 1-12**.

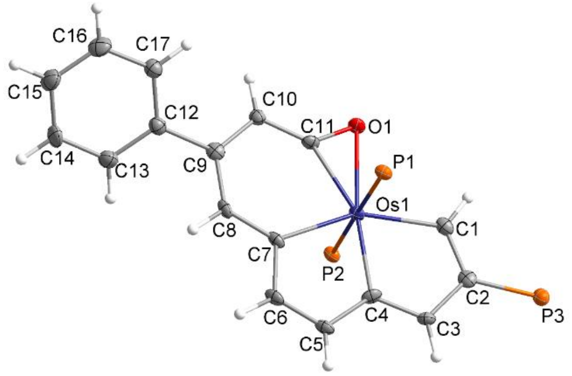

Supplementary Figure 80. X-ray molecular structure of the cation of complex **3a**. Thermal ellipsoids are set at 50% probability level. The counter ions and phenyl groups in PPh_3 moieties are omitted for clarity.

Supplementary Table 1. Selected bond lengths and bond angles for complex **3a**.

Bond Distances (Å)					
Os1–C1	2.091(4)	C3–C4	1.383(6)	C10–C11	1.393(5)
Os1–C4	2.079(4)	C4–C5	1.414(6)	C11–O1	1.249(5)
Os1–C7	2.110(4)	C5–C6	1.357(6)	C9–C12	1.495(6)
Os1–C11	2.001(4)	C6–C7	1.432(5)		
Os1–O1	2.215(3)	C7–C8	1.393(5)		
C1–C2	1.373(6)	C8–C9	1.403(6)		
C2–C3	1.442(6)	C9–C10	1.401(6)		
**Bond Angels (°)**					
Os1–C1–C2	117.2(3)	C5–C6–C7	116.9(3)	C9–C10–C11	117.4(3)
C1–C2–C3	114.9(3)	C6–C7–Os1	115.0(3)	Os1–C11–C10	142.4(3)
C2–C3–C4	113.6(3)	C7–Os1–C4	75.83(15)	C7–Os1–C11	80.09(15)
C3–C4–Os1	118.0(3)	C8–C7–Os1	129.6(3)	Os1–C11–O1	82.5(2)
C4–Os1–C1	76.24(16)	C7–C8–C9	127.4(4)	C11–O1–Os1	63.6(2)
Os1–C4–C5	118.5(3)	C8–C9–C10	122.7(3)	C11–Os1–O1	33.99(13)
C4–C5–C6	113.7(3)				

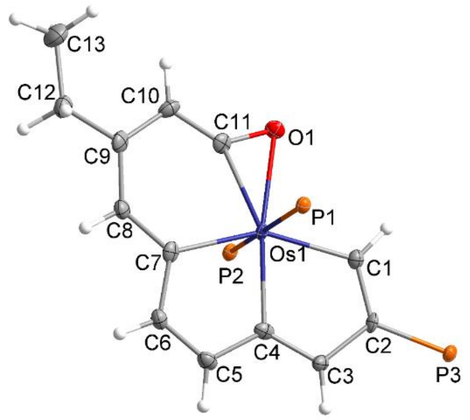

Supplementary Figure 81. X-ray molecular structure of the cation of complex **3c**. Thermal ellipsoids are set at 50% probability level. The counter ions and phenyl groups in PPh_3 moieties are omitted for clarity.

Supplementary Table 2. Selected bond lengths and bond angles for complex **3c**.

Bond Distances (Å)					
Os1–C1	2.081(4)	C3–C4	1.359(6)	C10–C11	1.375(6)
Os1–C4	2.089(4)	C4–C5	1.417(6)	C11–O1	1.256(5)
Os1–C7	2.109(4)	C5–C6	1.347(6)	C9–C12	1.502(6)
Os1–C11	2.009(4)	C6–C7	1.445(6)		
Os1–O1	2.217(3)	C7–C8	1.390(6)		
C1–C2	1.357(6)	C8–C9	1.404(6)		
C2–C3	1.437(5)	C9–C10	1.394(6)		
**Bond Angels (°)**					
Os1–C1–C2	118.2(3)	C5–C6–C7	116.5(4)	C9–C10–C11	118.0(4)
C1–C2–C3	114.3(3)	C6–C7–Os1	115.1(3)	Os1–C11–C10	142.4(3)
C2–C3–C4	114.1(3)	C7–Os1–C4	75.82(16)	C7–Os1–C11	79.99(16)
C3–C4–Os1	118.0(3)	C8–C7–Os1	128.8(3)	Os1–C11–O1	82.0(2)
C4–Os1–C1	75.39(15)	C7–C8–C9	128.2(4)	C11–O1–Os1	63.8(2)
Os1–C4–C5	117.9(3)	C8–C9–C10	122.0(4)	C11–Os1–O1	34.14(14)
C4–C5–C6	114.5(4)				

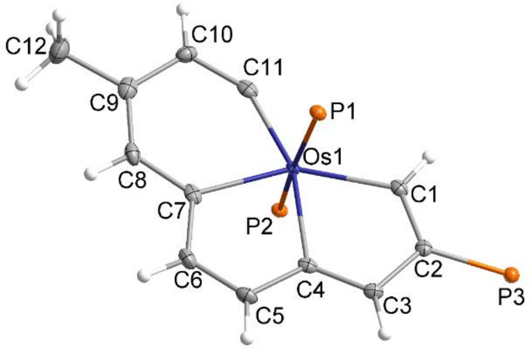

Supplementary Figure 82. X-ray molecular structure of the cation of complex **4b-Cl**. Thermal ellipsoids are set at 50% probability level. The counter ions and phenyl groups in PPh_3 moieties are omitted for clarity.

Supplementary Table 3. Selected bond lengths and bond angles for complex **4b-Cl**.

Bond Distances (Å)					
Os1–C1	2.0961(17)	C4–C5	1.422(2)	C9–C12	1.505(3)
Os1–C4	2.1342(17)	C5–C6	1.361(3)		
Os1–C7	2.0809(17)	C6–C7	1.439(3)		
Os1–C11	1.8503(18)	C7–C8	1.401(3)		
C1–C2	1.368(2)	C8–C9	1.388(3)		
C2–C3	1.450(2)	C9–C10	1.424(3)		
C3–C4	1.363(3)	C10–C11	1.356(3)		
Bond Angels (°)					
Os1–C1–C2	116.97(13)	C5–C6–C7	118.15(16)		
C1–C2–C3	116.56(16)	C6–C7–Os1	114.48(13)	C9–C10–C11	116.32(17)
C2–C3–C4	112.97(15)	C7–Os1–C4	77.47(7)	Os1–C11–C10	146.65(15)
C3–C4–Os1	117.85(13)	C8–C7–Os1	128.11(13)	C7–Os1–C11	81.04(7)
C4–Os1–C1	75.62(7)	C7–C8–C9	126.13(17)		
Os1–C4–C5	115.05(12)	C8–C9–C10	121.72(17)		
C4–C5–C6	114.82(16)				

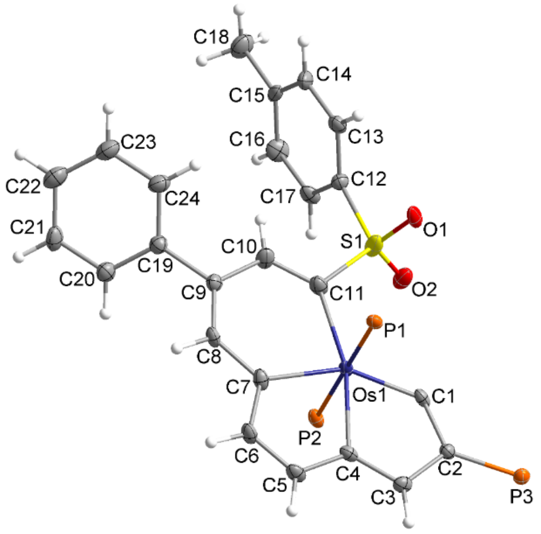

Supplementary Figure 83. X-ray molecular structure of the cation of complex **6**. Thermal ellipsoids are set at 50% probability level. The counter ions and phenyl groups in PPh_3 moieties are omitted for clarity.

Supplementary Table 4. Selected bond lengths and bond angles for complex **6**.

Bond Distances (Å)					
Os1–C1	1.848(3)	C4–C5	1.389(4)	C9–C19	1.522(4)
Os1–C4	2.131(3)	C5–C6	1.377(4)	C11–S1	1.771(3)
Os1–C7	2.071(3)	C6–C7	1.406(4)		
Os1–C11	2.099(3)	C7–C8	1.436(4)		
C1–C2	1.393(4)	C8–C9	1.359(4)		
C2–C3	1.428(4)	C9–C10	1.452(4)		
C3–C4	1.391(4)	C10–C11	1.353(4)		
**Bond Angles (°)**					
Os1–C1–C2	130.5(2)	C5–C6–C7	116.2(3)		
C1–C2–C3	108.2(2)	C6–C7–Os1	116.8(2)	C9–C10–C11	125.0(3)
C2–C3–C4	111.1(2)	C7–Os1–C4	76.00(11)	Os1–C11–C10	128.8(2)
C3–C4–Os1	117.7(2)	C8–C7–Os1	126.4(2)	C7–Os1–C11	88.02(12)
C4–Os1–C1	72.52(11)	C7–C8–C9	127.0(3)		
Os1–C4–C5	115.9(2)	C8–C9–C10	124.7(3)		
C4–C5–C6	115.1(3)				

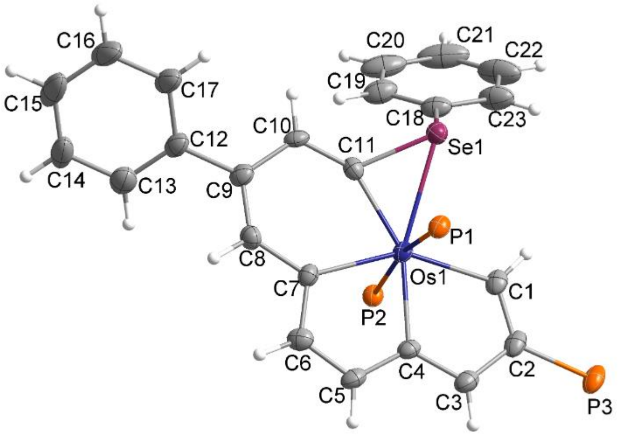

Supplementary Figure 84. X-ray molecular structure of the cation of complex **7**. Thermal ellipsoids are set at 50% probability level. The counter ions and phenyl groups in PPh_3 moieties are omitted for clarity.

Supplementary Table 5. Selected bond lengths and bond angles for complex **7**.

Bond Distances (Å)					
Os1–C1	2.084(4)	C3–C4	1.372(6)	C10–C11	1.329(6)
Os1–C4	2.120(4)	C4–C5	1.381(7)	C11–Se1	1.904(4)
Os1–C7	2.130(4)	C5–C6	1.368(6)	C9–C12	1.481(6)
Os1–C11	2.009(5)	C6–C7	1.407(7)	Se1–C18	1.940(5)
Os1–Se1	2.6093(5)	C7–C8	1.425(6)		
C1–C2	1.373(7)	C8–C9	1.367(7)		
C2–C3	1.416(7)	C9–C10	1.434(7)		
Bond Angels (°)					
Os1–C1–C2	119.2(4)	C5–C6–C7	117.0(4)	C9–C10–C11	118.2(4)
C1–C2–C3	114.9(4)	C6–C7–Os1	115.5(3)	Os1–C11–C10	147.0(4)
C2–C3–C4	113.0(4)	C7–Os1–C4	74.66(17)	C7–Os1–C11	76.48(17)
C3–C4–Os1	118.9(4)	C8–C7–Os1	130.6(3)	Os1–C11–Se1	83.59(17)
C4–Os1–C1	73.99(18)	C7–C8–C9	128.2(4)	C11–Se1–Os1	49.93(14)
Os1–C4–C5	118.4(3)	C8–C9–C10	119.2(4)	C11–Os1–Se1	46.47(13)
C4–C5–C6	114.4(4)				

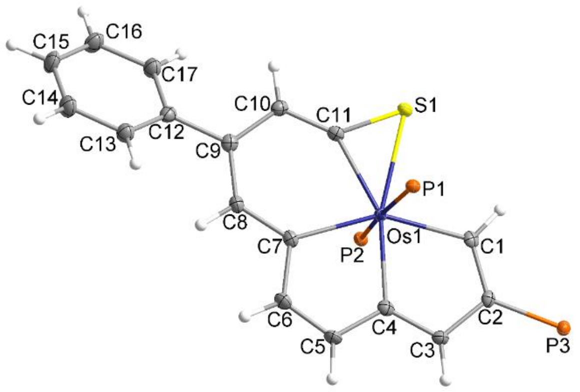

Supplementary Figure 85. X-ray molecular structure of the cation of complex **10**. Thermal ellipsoids are set at 50% probability level. The counter ions and phenyl groups in PPh_3 moieties are omitted for clarity.

Supplementary Table 6. Selected bond lengths and bond angles for complex **10**.

Bond Distances (Å)					
Os1–C1	2.087(2)	C3–C4	1.369(3)	C10–C11	1.357(3)
Os1–C4	2.101(2)	C4–C5	1.409(4)	C11–S1	1.667(3)
Os1–C7	2.128(2)	C5–C6	1.351(4)	C9–C12	1.487(3)
Os1–C11	2.046(2)	C6–C7	1.434(4)		
Os1–S1	2.4611(6)	C7–C8	1.406(3)		
C1–C2	1.371(3)	C8–C9	1.392(4)		
C2–C3	1.429(4)	C9–C10	1.405(4)		
**Bond Angels (°)**					
Os1–C1–C2	118.79(18)	C5–C6–C7	117.0(2)	C9–C10–C11	120.5(2)
C1–C2–C3	114.5(2)	C6–C7–Os1	114.93(17)	Os1–C11–C10	141.71(19)
C2–C3–C4	113.1(2)	C7–Os1–C4	75.37(9)	C7–Os1–C11	78.65(9)
C3–C4–Os1	119.09(19)	C8–C7–Os1	130.85(19)	Os1–C11–S1	82.36(10)
C4–Os1–C1	74.50(10)	C7–C8–C9	127.4(2)	C11–S1–Os1	55.47(8)
Os1–C4–C5	118.20(17)	C8–C9–C10	120.8(2)	C11–Os1–S1	42.16(7)
C4–C5–C6	114.4(2)				

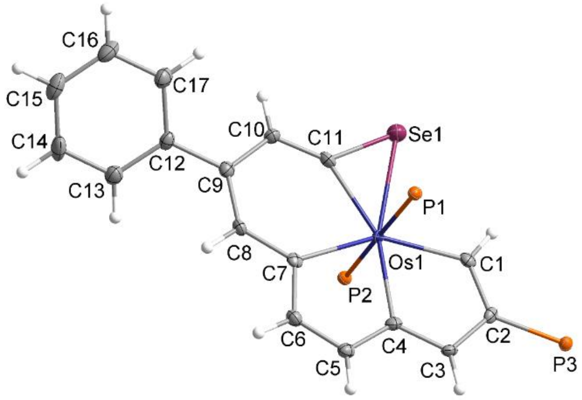

Supplementary Figure 86. X-ray molecular structure of the cation of complex **11**. Thermal ellipsoids are set at 50% probability level. The counter ions and phenyl groups in PPh_3 moieties are omitted for clarity.

Supplementary Table 7. Selected bond lengths and bond angles for complex **11**.

Bond Distances (Å)					
Os1–C1	2.099(4)	C3–C4	1.368(5)	C10–C11	1.340(5)
Os1–C4	2.107(4)	C4–C5	1.411(5)	C11–Se1	1.838(4)
Os1–C7	2.124(4)	C5–C6	1.348(5)	C9–C12	1.479(5)
Os1–C11	2.045(4)	C6–C7	1.432(5)		
Os1–Se1	2.5965(5)	C7–C8	1.413(5)		
C1–C2	1.376(5)	C8–C9	1.388(5)		
C2–C3	1.429(5)	C9–C10	1.419(5)		
Bond Angels (°)					
Os1–C1–C2	118.7(3)	C5–C6–C7	116.6(3)	C9–C10–C11	120.7(3)
C1–C2–C3	114.3(3)	C6–C7–Os1	115.6(3)	Os1–C11–C10	141.9(3)
C2–C3–C4	113.4(3)	C7–Os1–C4	75.03(14)	C7–Os1–C11	78.86(14)
C3–C4–Os1	119.2(3)	C8–C7–Os1	130.5(3)	Os1–C11–Se1	83.76(15)
C4–Os1–C1	74.28(14)	C7–C8–C9	127.5(3)	C11–Se1–Os1	51.53(12)
Os1–C4–C5	118.0(3)	C8–C9–C10	120.4(3)	C11–Os1–Se1	44.71(10)
C4–C5–C6	114.7(3)				

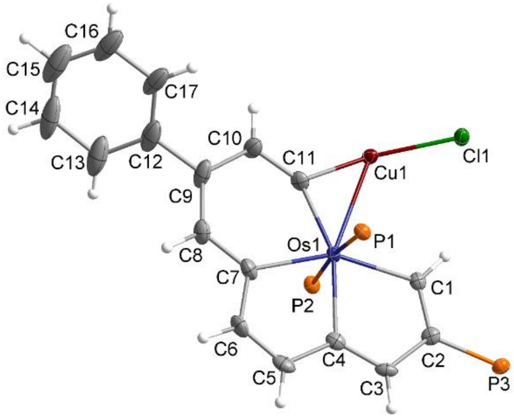

Supplementary Figure 87. X-ray molecular structure of the cation of complex **12**. Thermal ellipsoids are set at 50% probability level. The counter ions and phenyl groups in PPh_3 moieties are omitted for clarity.

Supplementary Table 8. Selected bond lengths and bond angles for complex **12**.

Bond Distances (Å)					
Os1–C1	2.105(5)	C3–C4	1.366(8)	C10–C11	1.351(8)
Os1–C4	2.119(5)	C4–C5	1.419(7)	C11–Cu1	1.891(5)
Os1–C7	2.120(5)	C5–C6	1.356(8)	C9–C12	1.494(9)
Os1–C11	1.902(5)	C6–C7	1.431(8)	Cu1–Cl1	2.2369(13)
Os1–Cu1	2.6339(7)	C7–C8	1.407(8)		
C1–C2	1.356(7)	C8–C9	1.407(9)		
C2–C3	1.436(7)	C9–C10	1.417(8)		
**Bond Angels (°)**					
Os1–C1–C2	116.7(4)	C5–C6–C7	118.5(5)	C9–C10–C11	118.5(5)
C1–C2–C3	116.9(5)	C6–C7–Os1	113.8(4)	Os1–C11–C10	146.6(4)
C2–C3–C4	113.0(5)	C7–Os1–C4	76.7(2)	C7–Os1–C11	78.8(2)
C3–C4–Os1	117.9(4)	C8–C7–Os1	130.1(4)	Os1–C11–Cu1	88.0(2)
C4–Os1–C1	75.4(2)	C7–C8–C9	125.5(5)	C11–Cu1–Os1	46.18(17)
Os1–C4–C5	116.2(4)	C8–C9–C10	120.3(5)	C11–Os1–Cu1	45.85(15)
C4–C5–C6	114.7(5)				

Supplementary Table 9. Crystal data and structure refinement for **3a•2CH$_2$Cl$_2$** and **3c**.

Identification code	**3a•2CH$_2$Cl$_2$**	**3c**
Empirical formula	$C_{73}H_{60}BCl_4F_4OOsP_3$	$C_{67}H_{56}BF_4OOsP_3$
Formula weight	1464.93	1247.03
Temperature/K	100.00	100.15
Crystal system	triclinic	monoclinic
Space group	P-1	P21/n
a/Å	12.0291(11)	10.1123(9)
b/Å	13.1920(11)	12.9394(13)
c/Å	20.1406(14)	40.798(4)
α/°	93.959(3)	90
β/°	92.995(4)	95.590(4)
γ/°	101.076(4)	90
Volume/Å^3	3122.0(4)	5312.9(9)
Z	2	4
ρ_{calc}g/cm^3	1.558	1.559
μ/mm^{-1}	6.633	5.875
F(000)	1472.0	2512.0
Crystal size/mm^3	0.5 × 0.5 × 0.5	0.1 × 0.1 × 0.1
Radiation	CuKα (λ = 1.54178)	CuKα (λ = 1.54178)
2Θ range/°	6.85 to 137.144	4.352 to 124.994
Reflections collected	54337	86864
Independent reflections	11473	8446
Data/restraints/parameters	11473/0/784	8446/0/695
Goodness-of-fit on F^2	1.036	1.189
Final R indexes [$I \geq 2\sigma (I)$]	R_1 = 0.0407, wR_2 = 0.1022	R_1 = 0.0356, wR_2 = 0.0863
Final R indexes [all data]	R_1 = 0.0476, wR_2 = 0.1065	R_1 = 0.0361, wR_2 = 0.0866

Largest diff. peak/hole / e Å^{-3}	1.57/-0.93	2.26/-1.06

Supplementary Table 10. Crystal data and structure refinement for **4b-Cl•2CH$_2$Cl$_2$** and **6**.

Identification code	**4b-Cl•2CH$_2$Cl$_2$**	**6**
Empirical formula	$C_{68}H_{58}Cl_5OsP_3$	$C_{102}H_{82}BO_2OsP_3S$
Formula weight	1335.50	1665.65
Temperature/K	100.0(2)	100.00
Crystal system	triclinic	monoclinic
Space group	P-1	P2$_1$/c
a/Å	11.9970(7)	9.8445(6)
b/Å	14.0672(6)	39.822(2)
c/Å	19.1528(9)	20.0501(14)
α/°	80.484(2)	90
β/°	88.647(2)	96.950(3)
γ/°	66.647(3)	90
Volume/Å^3	2923.5(3)	7802.4(8)
Z	2	4
ρ_{calc}g/cm^3	1.517	1.418
μ/mm^{-1}	1.517	4.331
F(000)	1344.0	3408.0
Crystal size/mm^3	0.03 × 0.02 × 0.02	0.1 × 0.1 × 0.1
Radiation	CuKα (λ = 1.54178)	CuKα (λ = 1.54178)
2Θ range/°	4.682 to 133.646	4.438 to 133.618
Reflections collected	41105	103095
Independent reflections	10337	13795
Data/restraints/parameters	10337/0/695	13795/167/1168

Goodness-of-fit on F^2	1.077	1.084
Final R indexes [$I \geq 2\sigma(I)$]	R_1 = 0.0169, wR_2 = 0.0382	R_1 = 0.0326, wR_2 = 0.0733
Final R indexes [all data]	R_1 = 0.0177, wR_2 = 0.0385	R_1 = 0.0366, wR_2 = 0.0754
Largest diff. peak/hole / e Å^{-3}	0.47/-0.45	1.05/-0.96

Supplementary Table 11. Crystal data and structure refinement for **7** and **10•2CH$_2$Cl$_2$**.

Identification code	**7**	**10•2CH$_2$Cl$_2$**
Empirical formula	$C_{77}H_{61}Cl_2OsP_3Se$	$C_{97}H_{80}BCl_4OsP_3S$
Formula weight	1419.22	1713.39
Temperature/K	100.0(2)	100.00
Crystal system	monoclinic	triclinic
Space group	C2/c	P-1
a/Å	39.390(3)	11.3266(10)
b/Å	13.3071(9)	17.2548(16)
c/Å	26.3902(16)	20.8175(17)
α/°	90	82.550(3)
β/°	99.336(7)	79.134(3)
γ/°	90	87.461(5)
Volume/Å^3	13649.7(16)	3961.0(6)
Z	8	2
ρ_{calc}g/cm^3	1.381	1.437
μ/mm^{-1}	5.836	5.473
F(000)	5696.0	1744.0
Crystal size/mm^3	0.12 × 0.11 × 0.1	0.11 × 0.11 × 0.1
Radiation	CuKα (λ = 1.54178)	CuKα (λ = 1.54178)
2Θ range/°	4.546 to 134.012	4.356 to 133.53

Reflections collected	84917	57611
Independent reflections	12121	13975
Data/restraints/parameters	12121/0/766	13975/0/964
Goodness-of-fit on F^2	1.032	1.050
Final *R* indexes [$I \geq 2\sigma(I)$]	R_1 = 0.0390, wR_2 = 0.0968	R_1 = 0.0260, wR_2 = 0.0606
Final *R* indexes [all data]	R_1 = 0.0532, wR_2 = 0.1036	R_1 = 0.0280, wR_2 = 0.0615
Largest diff. peak/hole / e Å^{-3}	1.13/-1.16	2.02/-1.01

Supplementary Table 12. Crystal data and structure refinement for **11•$CHCl_3$** and **12•3$CHCl_3$**.

Identification code	**11•$CHCl_3$**	**12•3$CHCl_3$**
Empirical formula	$C_{96}H_{77}BCl_3OsP_3Se$	$C_{74}H_{59}Cl_{11}CuOsP_3$
Formula weight	1709.80	1684.81
Temperature/K	100.00	100.00
Crystal system	monoclinic	triclinic
Space group	P21/c	P-1
a/Å	12.0301(6)	11.9220(4)
b/Å	18.7362(8)	17.5387(7)
c/Å	34.2125(13)	18.6382(7)
α/°	90	66.199(2)
β/°	94.216(3)	84.874(2)
γ/°	90	86.157(2)
Volume/Å^3	7690.6(6)	3549.4(2)
Z	4	2
ρ_{calc}g/cm^3	1.477	1.576
μ/mm^{-1}	5.596	8.477
F(000)	3456.0	1680.0

Crystal size/mm^3	0.22 × 0.2 × 0.2	0.1 × 0.1 × 0.08
Radiation	CuKα (λ = 1.54178)	CuKα (λ = 1.54178)
2Θ range/°	5.18 to 137.454	5.194 to 133.664
Reflections collected	114335	51917
Independent reflections	14213	12493
Data/restraints/parameters	14213/0/946	12493/118/903
Goodness-of-fit on F^2	1.037	1.042
Final R indexes [$I \geq 2\sigma(I)$]	R_1 = 0.0352, wR_2 = 0.0808	R_1 = 0.0442, wR_2 = 0.1042
Final R indexes [all data]	R_1 = 0.0451, wR_2 = 0.0848	R_1 = 0.0556, wR_2 = 0.1102
Largest diff. peak/hole / e $Å^{-3}$	1.71/-1.70	1.60/-1.27

8. Theoretical calculations

Computational Results

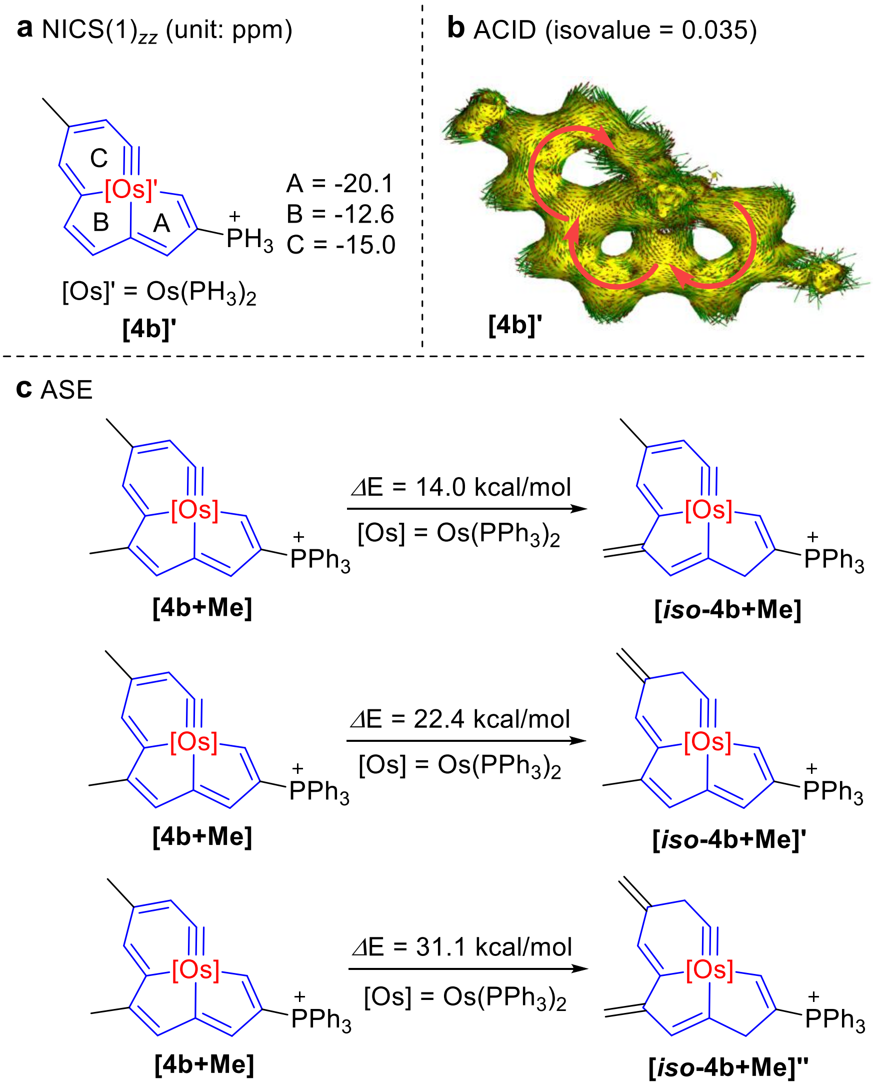

Supplementary Figure 88. Evaluation of aromaticity for complex **4b** by DFT calculations. (a) NICS(1)$_{zz}$ values of the model complex **[4b]'**; (b) ACID plot of **[4b]'** with an isosurface value of 0.035. The magnetic field vector is orthogonal to the ring plane and points upward (aromatic species exhibit clockwise diatropic circulations); (c) ASE values calculated on the model complex **[4b+Me]**.

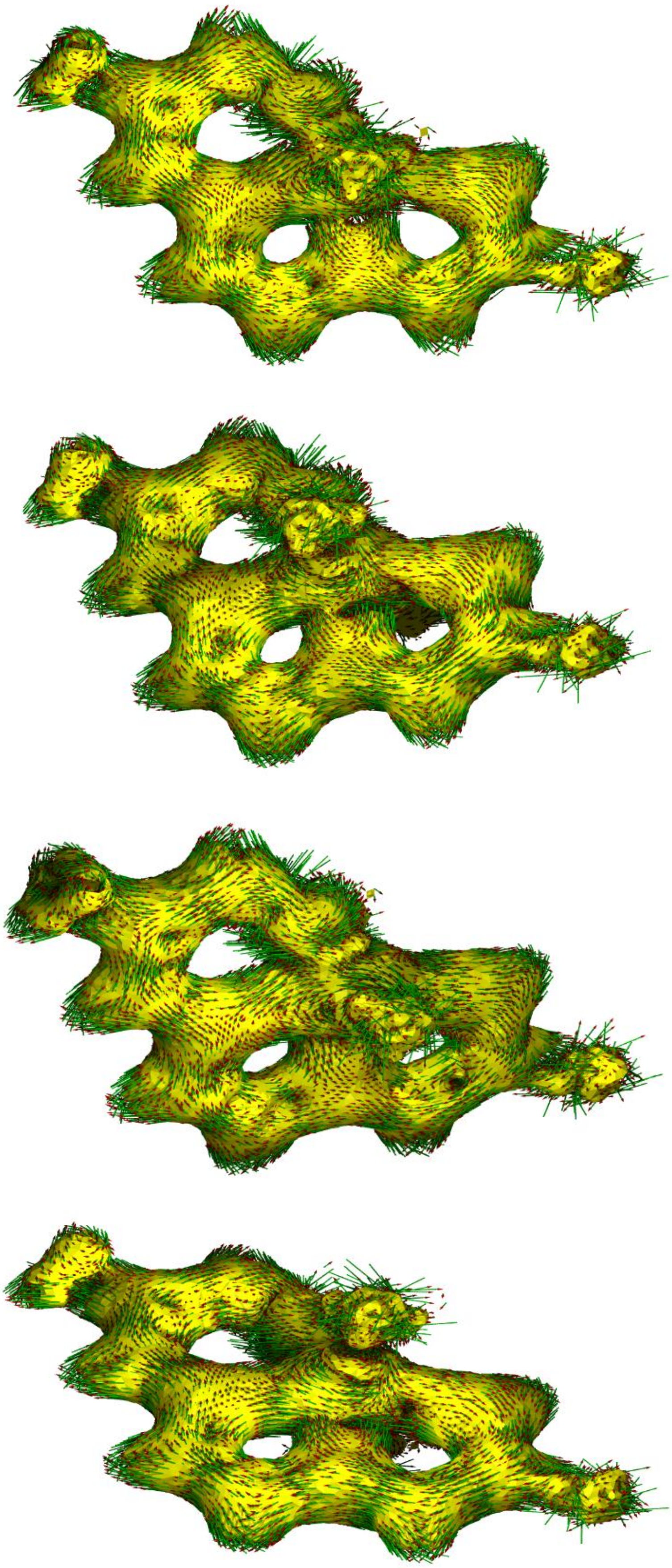

Supplementary Figure 89. Views for ACID plots of **[4b]'** in different directions.

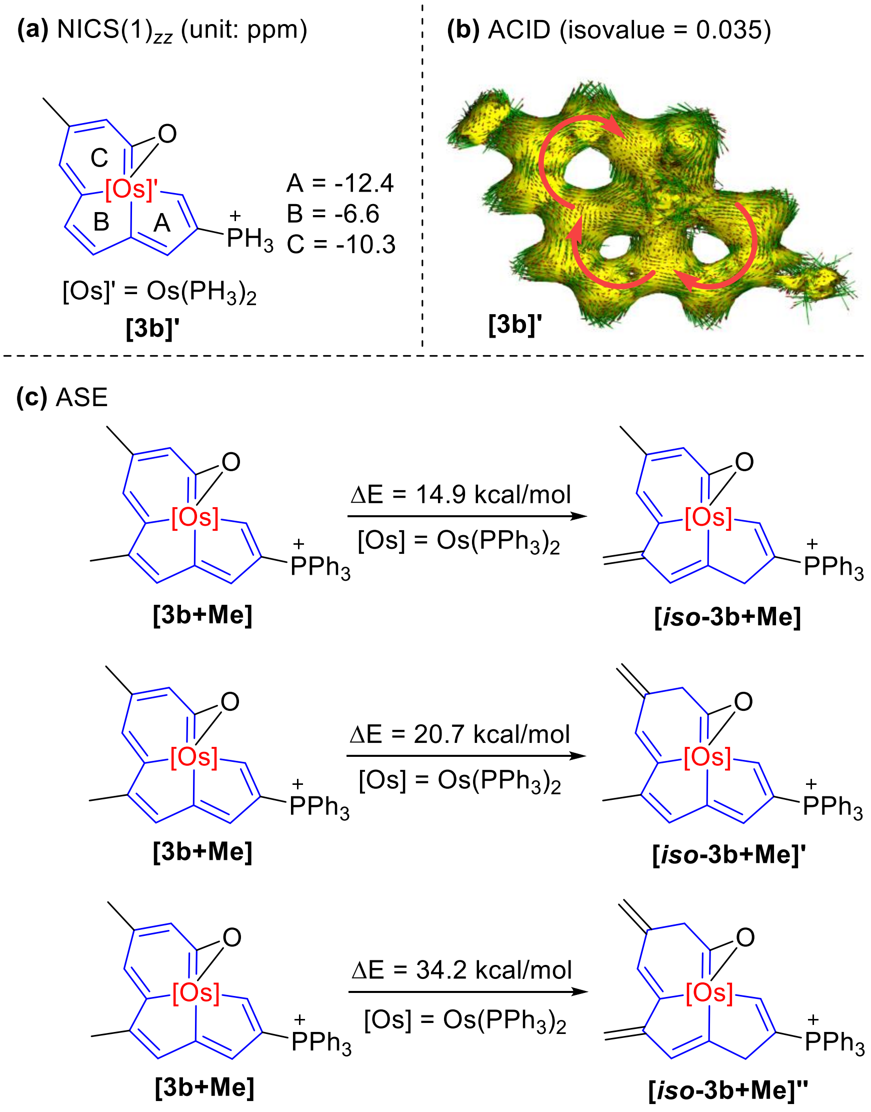

Supplementary Figure 90. Evaluation of aromaticity for complex **3b** by DFT calculations. (a) NICS(1)$_{zz}$ values of the model complex **[3b]'**; (b) ACID plot of **[3b]'** with an isosurface value of 0.035. The magnetic field vector is orthogonal to the ring plane and points upward (aromatic species exhibit clockwise diatropic circulations); (c) ASE values calculated on the model complex **[3b+Me]**.

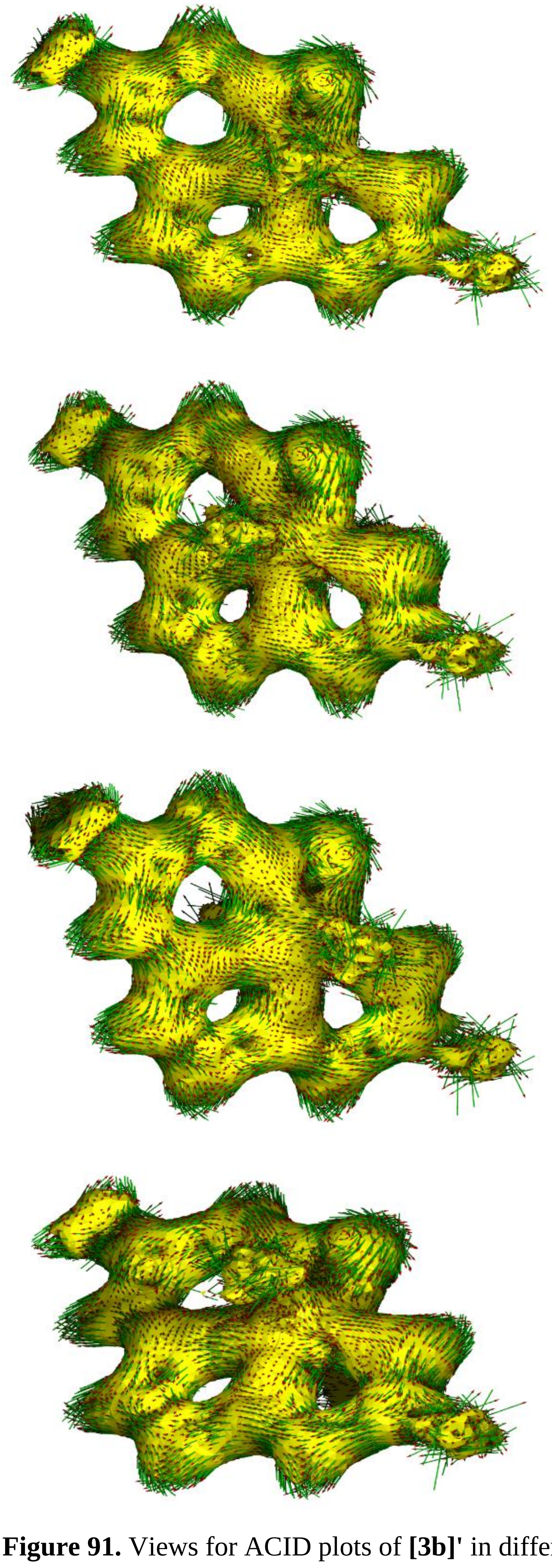

Supplementary Figure 91. Views for ACID plots of **[3b]'** in different directions.

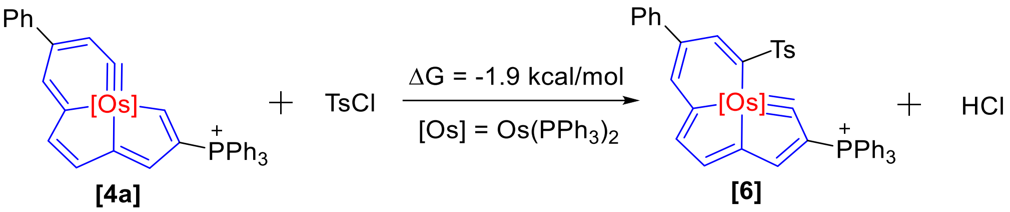

Supplementary Figure 92. Calculated energy for the reaction of **[4a]** with TsCl.

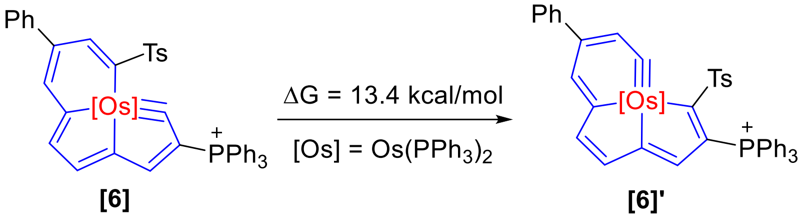

Supplementary Figure 93. Energy comparison of compounds **[6]** and **[6]'**.

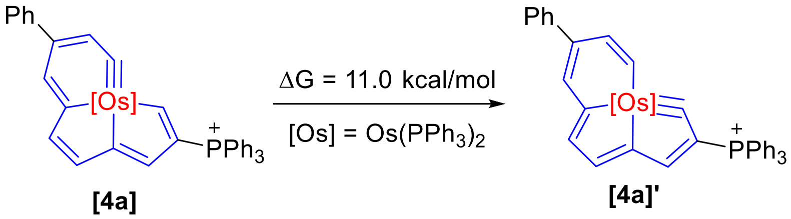

Supplementary Figure 94. Energy comparison of compounds **[4a]** and **[4a]'**.

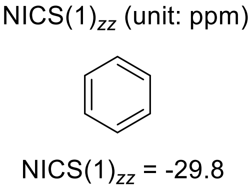

Supplementary Figure 95. NICS(1)$_{zz}$ values of benzene.

Computational Details

All structures were optimized at the B3LYP level of density functional theory[4-6]. Frequency calculations were performed to identify all the stationary points as minima. All these structures evaluated were optimized by B3LYP-D3BJ/Def2-TZVP[7]. As shown in Table S13-15, the structural parameters obtained from calculations with the B3LYP functional are consistent with the X-ray structure of **4b**, **3a** and **6**, with relative deviations (RD) of 0.33%, 0.62% and 0.55%, which indicates the outperformance of this method. Compounds **[4b]'** and **[3b]'** are simplified through the way that the PH_3 groups were used to replace the PPh_3 groups in **[4b]** and **[3b]**, the counter ions are omitted for clarity during calculations. NICS values were calculated at the B3LYP-GIAO/Def2-TZVP level and the ACID calculations were carried out at the same level with NICS with the ACID program[8,9].

The single-point energy calculations were performed on Gibbs free energy (ΔG) of the reactions using the B3LYP-D3BJ/Def2-TZVP method with the SMD solvation method[10,11]. All calculations were done in Gaussian 16 program package, Revision A.03[12].

Supplementary Table 13. Comparison of bond lengths in **4b** from the DFT calculated and experimental data.

**4b**							
Bond Distances (Å)	Os1–C1	Os1–C4	Os1–C7	Os1–C11	C1–C2	C2–C3	C3–C4
Crystallographic data	2.0961	2.1342	2.0809	1.8603	1.368	1.450	1.363
B3LYP-D3(BJ)/ def2-TZVP	2.108	2.1427	2.0976	1.8526	1.371	1.446	1.364
Relative deviations (%)	0.5677	0.3983	0.8025	0.4139	0.2193	0.2759	0.0734
Bond Distances (Å)	C4–C5	C5–C6	C6–C7	C7–C8	C8–C9	C9–C10	C10–C11
Crystallographic data	1.422	1.361	1.439	1.401	1.388	1.424	1.356
B3LYP-D3(BJ)/ def2-TZVP	1.418	1.365	1.437	1.403	1.389	1.421	1.346
Relative deviations (%)	0.2813	0.2939	0.1390	0.1428	0.0720	0.2107	0.7375
Mean relative deviations (%)	0.3306						

Supplementary Table 14. Comparison of bond lengths in **3a** from the DFT calculated and experimental data.

**3a**							
Bond Distances (Å)	Os1–C1	Os1–C4	Os1–C7	Os1–C11	C1–C2	C2–C3	C3–C4
Crystallographic data	2.091	2.079	2.110	2.001	1.373	1.442	1.383
B3LYP-D3(BJ)/ def2-TZVP	2.089	2.094	2.135	2.021	1.369	1.44	1.365
Relative deviations (%)	0.0956	0.7215	1.1848	0.9995	0.2913	0.1387	1.3015
Bond Distances (Å)	C4–C5	C5–C6	C6–C7	C7–C8	C8–C9	C9–C10	C10–C11
Crystallographic data	1.414	1.357	1.432	1.393	1.403	1.401	1.393
B3LYP-D3(BJ)/ def2-TZVP	1.421	1.354	1.439	1.387	1.405	1.394	1.37
Relative deviations (%)	0.4950	0.2211	0.4888	0.4307	0.1426	0.4996	1.6511
Mean relative deviations (%)	0.6187						

Supplementary Table 15. Comparison of bond lengths in **6** from the DFT calculated and experimental data.

**6**							
Bond Distances (Å)	Os1–C1	Os1–C4	Os1–C7	Os1–C11	C1–C2	C2–C3	C3–C4
Crystallographic data	1.848	2.131	2.071	2.099	1.393	1.428	1.391
B3LYP-D3(BJ)/ def2-TZVP	1.844	2.156	2.078	2.104	1.381	1.433	1.383
Relative deviations (%)	0.2164	1.1731	0.3380	0.2382	0.8615	0.3501	0.5751
Bond Distances (Å)	C4–C5	C5–C6	C6–C7	C7–C8	C8–C9	C9–C10	C10–C11
Crystallographic data	1.389	1.377	1.406	1.436	1.359	1.452	1.353
B3LYP-D3(BJ)/ def2-TZVP	1.397	1.382	1.409	1.423	1.373	1.446	1.347
Relative deviations (%)	0.5760	0.3631	0.2134	0.9053	1.0301	0.4132	0.4435
Mean relative deviations (%)	0.5498						

9. Photothermal performance

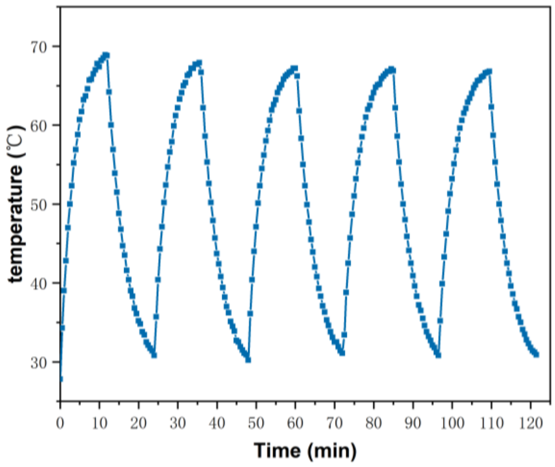

Supplementary Figure 96. Photothermal stability of **7** (0.1 mg/mL in DMSO) upon 808 nm (1 W/cm^{-2}) laser irradiation for five on/off cycles.

10. References

1. Zhu, C.; Zhou, X.; Xing, H.; An, K.; Zhu, J.; Xia, H. σ-Aromaticity in an Unsaturated Ring: Osmapentalene Derivatives Containing a Metallacyclopropene Unit. *Angew. Chem., Int. Ed.* **2015**, *54*, 3102-3106.

2. Dolomanov, O. V.; Bourhis, L. J.; Gildea, R. J.; Howard, J. A. K.; Puschmann, H. *Olex2*: a Complete Structure Solution, Refinement and Analysis Program. *J. Appl. Cryst.* **2009**, *42*, 339-341.

3. Sheldrick, G. M. *SHELXT* – Integrated Space-group and Crystal-structure Determination. *Acta. Cryst. Sect. A* **2015**, *71*, 3-8.

4. Sheldrick, G. M. Crystal Structure Refinement with *SHELXL*. *Acta. Cryst. Sect. C* **2015**, *71*, 3-8.

5. Lee, C.; Yang, W.; Parr, R. G. Development of the Colle-Salvetti Correlation-Energy Formula into a Functional of the Electron Density. *Phys. Rev. B* **1988**, *37*, 785–789.

6. Miehlich, B.; Savin, A.; Stoll, H.; Preuss, H. Results Obtained with the Correlation Energy Density Functionals of Becke and Lee, Yang and Parr. *Chemical Phys. Lett.* **1989**, *157*, 200–206.

7. Becke, A. D. Density-Functional Thermochemistry. III. The Role of Exact Exchange. *J. Chem. Phys.* **1993**, *98*, 5648–5652.

8. Herges, R.; Geuenich, D. Delocalization of Electrons in Molecules. *J. Phys. Chem. A* **2001**, *105*, 3214–3220.

9. Geuenich, D.; Hess, K.; Köhler, F.; Herges, R. Anisotropy of the Induced Current Density (ACID), a General Method To Quantify and Visualize Electronic Delocalization. *Chem. Rev.* **2005**, *105*, 3758–3772.

10. Weigend, F. & Ahlrichs, R. Balanced basis sets of split valence, triple zeta valence and quadruple zeta valence quality for H to Rn: Design and assessment of accuracy. *Phys. Chem. Chem. Phys.* **2005**, *7*, 3297-3305.

11. Huzinaga, S. Basis-Sets for Molecular Calculations. *Comput. Phys. Rep.* 1985, *2*, 281-339.

12. Gaussian 16, Revision A.03. M. J. Frisch, G. W. Trucks, H. B. Schlegel, G. E. Scuseria, M. A. Robb, J. R. Cheeseman, G. Scalmani, V. Barone, G. A. Petersson, H. Nakatsuji, X. Li, M. Caricato, A. V. Marenich, J. Bloino, B. G. Janesko, R. Gomperts, B. Mennucci, H. P. Hratchian, J. V. Ortiz, A. F. Izmaylov, J. L. Sonnenberg, D. Williams-Young, F. Ding, F. Lipparini, F. Egidi, J. Goings, B. Peng, A. Petrone, T. Henderson, D. Ranasinghe, V. G. Zakrzewski, J. Gao, N. Rega, G. Zheng, W. Liang, M. Hada, M. Ehara, K. Toyota, R. Fukuda, J. Hasegawa, M. Ishida, T. Nakajima, Y. Honda, O. Kitao, H. Nakai, T. Vreven, K. Throssell, J. A. Montgomery, Jr., J. E. Peralta, F. Ogliaro, M. J. Bearpark, J. J. Heyd, E. N. Brothers, K. N. Kudin, V. N. Staroverov, T. A. Keith, R. Kobayashi, J. Normand, K. Raghavachari, A. P. Rendell, J. C. Burant, S. S. Iyengar, J. Tomasi, M. Cossi, J. M. Millam, M. Klene, C. Adamo, R. Cammi, J. W. Ochterski, R. L. Martin, K. Morokuma, O. Farkas, J. B. Foresman, and D. J. Fox, Gaussian, Inc., Wallingford CT, **2016**.
